# Bayesian Sparse Gaussian Mixture Model for Clustering in High Dimensions

**Published:** 2025

**Authors:** Dapeng Yao, Fangzheng Xie, Yanxun Xu

**Affiliations:** Department of Applied Mathematics and Statistics, Johns Hopkins University, Baltimore, Maryland, U.S.A.; Department of Statistics, Indiana University, Bloomington, Indiana, U.S.A.; Department of Applied Mathematics and Statistics, Johns Hopkins University, Baltimore, Maryland, U.S.A.

**Keywords:** Clustering, High dimensions, Minimax estimation, Posterior contraction, Single-cell sequencing

## Abstract

We study the sparse high-dimensional Gaussian mixture model when the number of clusters is allowed to grow with the sample size. A minimax lower bound for parameter estimation is established, and we show that a constrained maximum likelihood estimator achieves the minimax lower bound. However, this optimization-based estimator is computationally intractable because the objective function is highly nonconvex and the feasible set involves discrete structures. To address the computational challenge, we propose a computationally tractable Bayesian approach to estimate high-dimensional Gaussian mixtures whose cluster centers exhibit sparsity using a continuous spike-and-slab prior. We further prove that the posterior contraction rate of the proposed Bayesian method is minimax optimal. The mis-clustering rate is obtained as a by-product using tools from matrix perturbation theory. The proposed Bayesian sparse Gaussian mixture model does not require pre-specifying the number of clusters, which can be adaptively estimated. The validity and usefulness of the proposed method is demonstrated through simulation studies and the analysis of a real-world single-cell RNA sequencing data set.

## 1. Introduction

Clustering is a powerful tool for detecting structures in heterogeneous data and identifying homogeneous subgroups with a wide range of applications, such as genomics (Gu and Liu, 2008), pattern recognition (Diday et al., 1981), and topic modeling (Blei et al., 2003). In many scientific domains, data are often high-dimensional, i.e., the dimension of observations can be larger than the sample size. For example, an important task in the analysis of single-cell RNA-sequencing data, where the number of genes (dimension) is usually larger than the number of cells (sample size), is to cluster cells and identify functional cell subpopulations (Cao et al., 2019). A principal challenge of extending the low-dimensional clustering techniques to high dimensions is the well-known “curse of dimensionality.” To overcome this issue, dimensionality reduction (Ding et al., 2002) or additional structural assumptions (Cai et al., 2019) are usually necessary in high dimensional clustering methods.

High dimensional clustering and mixture models have attracted attention recently from the frequentist perspective. When the dimension p has at most the same order as the sample size n, Doss et al. (2023) studied the optimal rate of estimation in a finite Gaussian location mixture model without a separation condition. Löffler et al. (2021) showed that spectral clustering is minimax optimal in the Gaussian mixture model with isotropic covariance matrix when p=o(nΔ), where Δ is the minimal distance among cluster centers. When p≫n, Azizyan et al. (2013) considered a simple case in which there are only two clusters with equal mixing weights and same isotropic covariance matrices. Jin and Wang (2016) and Jin et al. (2017) proposed influential features principal component analysis based on feature selection and principal component analysis. A phase transition phenomenon in high dimensional clustering problem was also investigated in Jin and Wang (2016) and Jin et al. (2017) across different sparsity and signal levels. Cai et al. (2019) proposed a modified Expectation-Maximization (EM) algorithm based on sparse discriminant vectors to obtain the minimax optimal convergence rate of the excess mis-clustering error. In terms of density estimation, Ashtiani et al. (2020) obtained a near-optimal convergence rate for high dimensional location-scale mixtures with respect to the total variation distance.

Despite these theoretical and computational developments in high dimensional clustering, most frequentist approaches dealing with finite mixtures assume that the number of clusters K is either known or needs to be estimated consistently using techniques such as cross-validation (Smyth, 2000) and the gap statistics (Tibshirani et al., 2001). In contrast, Bayesian methods treat K as an unknown parameter and put a prior on it. For example, Miller and Harrison (2018) proposed a mixture of finite mixtures model with a Gibbs sampler, and the posterior consistency of K was later studied in Miller (2023). Ohn and Lin (2022) established a near optimal rate for estimating finite Gaussian mixtures with respect to the Wasserstein distance when K is unknown and allowed to grow with n. In the context of Bayesian model-based clustering for high-dimensional data, Tadesse et al. (2005) proposed a method for uncovering cluster structure and identifying the discriminating variables simultaneously and exploited reversible jump Markov chain Monte Carlo (MCMC) for posterior sampling. Gao et al. (2020) proposed a Bayesian structured linear model, which encompasses the bi-clustering problem as a special case, using a subset-selection prior and establishing an optimal posterior contraction rate for parameter estimation. Chandra et al. (2022) proposed a Bayesian latent factor mixture model and investigated the behavior of the induced cluster memberships as p goes to infinity whereas n remains fixed. However, a general theoretical framework for Bayesian analysis of high-dimensional clustering in terms of both parameter estimation and mis-clustering error is yet to be established.

The Gaussian mixture model we consider lies in the regime of high dimensionality with sparsity structures. There has been a growing interest in Bayesian inference with sparsity-enforcing priors. One commonly-used prior is the spike-and-slab prior (Mitchell and Beauchamp, 1988), which is a mixture of a point mass at zero and a relatively “flat” absolutely continuous density. The spike-and-slab LASSO prior (Ročková and George, 2018) borrows the similarity between the LASSO and Laplace prior, and combines it with a continuous version of the spike-and-slab prior. Theoretical properties of the spike-and-slab LASSO prior were well studied in the context of regression models, graphical models, and Gaussian sequence models (see Bai et al., 2021 for a review). Another class of sparsity-enforcing priors is global-local shrinkage priors, such as the horseshoe prior (Carvalho et al., 2009) and the Dirichlet-Laplace prior (Bhattacharya et al., 2015). We refer the readers to Tadesse and Vannucci (2021) and references therein. However, when these types of priors are adapted to high-dimensional sparse clustering problems with an unknown number of clusters, their theoretical properties remain unclear.

This paper presents the Bayesian analysis of a high-dimensional sparse Gaussian mixture model using a spike-and-slab LASSO prior and establishes the optimality of the proposed estimation procedure. Our main contribution is threefold. First, we fully characterize the minimax rate for parameter estimation in the high-dimensional sparse Gaussian mixture model, in which the number of clusters is allowed to grow with sample size. A frequentist constrained maximum likelihood estimator (MLE) is proved to achieve minimax optimality. Second, since the frequentist optimization-based estimator is computationally intractable, we propose a Bayesian sparse Gaussian mixture model to address this computational challenge, and establish its theoretical properties by showing that the posterior contraction rate for parameter estimation is minimax optimal. Third, we obtain a posterior contraction rate for the mis-clustering error using tools from matrix perturbation theory (Yu et al., 2014). To the best of our knowledge, this paper represents the first effort in establishing the theoretical results of parameter estimation and clustering recovery in high-dimensional Bayesian sparse Gaussian mixture model with a growing number of clusters.

The rest of this paper is organized as follows. In Section 2, we introduce the high-dimensional clustering problem and our model, establish the minimax lower bound for parameter estimation, and propose a frequentist constrained MLE that achieves the minimax lower bound. Section 3 elaborates on the main theoretical results, including the optimal posterior contraction rate and the mis-clustering error. We demonstrate the practical performance of the proposed method through simulation studies in Section 4 and a real-world application to clustering single-cell RNA sequencing data in Section 5.

**Notations:** Let |S| denote the cardinality of S if the set S is finite or the volume (Lebesgue measure) of S if S is a Lebesgue-measurable infinite subset in Euclidean space. Denote [n] as the set of all consecutive integers {1,…,n}. We use ≲ and ≳ to denote the inequality up to a constant. In other words, a≲ (≳, resp.) b if a≤ (≥, resp.) Cb for some constant C>0. We write a≍b if a≲b and b≲a. We use ⌊x⌋ to denote the greatest integer less than or equal to the real number x and ⌈x⌉ to denote the smallest integer greater than or equal to the real number x. For a p-dimensional vector $x\in R^{p}$, we denote $x_{i}$ as the ith coordinate of x. Also, we denote $\|x{\|}_{1}=\sum_{i=1}^{p}\left|x_{i}\right|$ as the $\ell_{1}$-norm, $\|x{\|}_{2}=\sum_{i=1}^{p}{\left|x_{i}\right|}^{2}$ as the $\ell_{2}$-norm, $\|x{\|}_{\infty }={\text{max}}_{i\in [p]}\left|x_{i}\right|$ as the $\ell_{\infty }$-norm, and $\|x{\|}_{0}=\sum_{i=1}^{p}1\left(x_{i}\neq 0\right)$. For any matrix $A\in R^{n\times m}$, let $A_{ij}$ denote the (i,j)-entry of A and let $A_{i}$. and $A_{\cdot j}$ be the ith row and jth column of A, respectively. We denote $\|A{\|}_{F}=\sqrt{\sum_{i=1}^{n}\sum_{j=1}^{m}{\left|A_{ij}\right|}^{2}}$ to be the Frobenius norm of A and $\|A{\|}_{2}$ to be the spectral norm of A. We denote A≻0 if A is a positive definite matrix. The prior and posterior probability distributions are denoted as Π and the corresponding densities with respect to the underlying σ-finite measure (whenever it exists) are denoted as π. We denote $D_{KL}(P\|Q)$ the Kullback–Leibler divergence between any probability measures P and Q. The ϵ-packing number of a metric space Θ with respect to the metric d, which is the maximum number of pairwise disjoint balls contained in Θ with radii ϵ, is denoted as M(ϵ,Θ,d). In the rest of the paper, we will use an asterisk to represent the ground true values of the parameters that give rise to the data distribution.

## 2. Model

This section introduces a Gaussian mixture model for high-dimensional clustering, establishing a minimax lower bound and proposing a Bayesian approach for tractable inference.

### 2.1 Gaussian mixture model and clustering

Let $Y=\left[Y_{1},\ldots ,Y_{n}\right]$ be a p×n data matrix, where rows represent variables or features, and columns represent observations. We assume that the data exhibits a clustering structure that can be described through a Gaussian mixture model as follows. Let $\mu_{1},\ldots ,\mu_{K}\in R^{p}$ be the cluster centroids of the respective clusters, where K≥1 is the number of clusters. Let $z={\left(z_{1},\ldots ,z_{n}\right)}^{T}\in [K{]}^{n}$ be the cluster membership vector for observations, with $z_{i}=k$ indicating that $Y_{i}$ belongs to the kth cluster. The distribution of $Y_{i}$ is given by

(1) $$Y_{i}=\mu_{z_{i}}+\epsilon_{i},$$

where $\epsilon_{i}\sim N_{p}(0,\Sigma )$ independently for i∈[n]. The goal is to estimate the cluster centroids $\mu_{1},\ldots ,\mu_{K}$ as well as to recover the latent cluster membership vector z.

This paper considers the asymptotic regime where both p and n go to infinity and p/n→∞. When p does not exceed n, Azizyan et al. (2013) proved that the expected clustering accuracy (which will be defined formally later) depends on the dimension p through the rate $\sqrt{p/n}$ in the two-cluster problem without additional structural assumptions. Under the regime that p/n→∞ considered in our framework, we posit the following sparse structure on the cluster mean vectors $\mu_{1},\ldots ,\mu_{K}$. Denote $\mu =\left[\mu_{1},\ldots ,\mu_{K}\right]$ as the matrix concatenated by the mean vectors of all clusters and define the support of μ as $\text{supp}(\mu )=\left\{j\in [p]:{\left(\mu_{j}.\right)}^{T}\neq 0\right\}$. We say that μ is jointly s-sparse if |supp(μ)|≤s. Moreover, we require that not only each $\mu_{k}$ has at most $s_{n}$ non-zero coordinates, namely, $\sum_{j=1}^{p}1(\mu_{kj}\neq 0)\leq s_{n}$ for all k∈[K], but also that μ is jointly $s_{n}$-sparse. We assume $s_{n}\to \infty$ as n→∞. In the sequel, we will drop the subscript n from $s_{n}$ and write $s=s_{n}$ for notation simplicity, but the readers should be reminded that s depends on n implicitly.

Denote $e_{i}\in R^{K}$ the unit vector that has value 1 at the ith coordinate and 0 elsewhere. Let $L={\left[l_{1},\ldots ,l_{n}\right]}^{T}\in R^{n\times K}$ where $l_{i}=e_{z_{i}}$. Then L is the matrix whose rows represent cluster memberships of the n observations. It follows immediately that the expected data matrix can be written as $E(Y)=\mu L^{T}$. Namely, our model can be represented as a signal-plus-noise model matrix $Y=\mu L^{T}+E$, where $E=\left[\epsilon_{1},\ldots ,\epsilon_{n}\right]$ is the mean-zero noise matrix where $\epsilon_{i}$’s are independent normal random vectors with mean zero and covariance matrix Σ. As K is typically much smaller than n, the above representation of the model is similar to those in Cape et al. (2018) and Agterberg et al. (2022) because the data matrix has a low expected rank. Nevertheless, the sparse Gaussian mixture model differs from Cape et al. (2018) and Agterberg et al. (2022) in that the columns of the expected data matrix have the clustering structure and the rows have the sparsity structure. Following the previous convention of using asterisk to indicate true parameter values, we denote $\left(\mu^{*},L^{*},\Sigma^{*}\right)$ the underlying truth of (μ,L,Σ) throughout the rest of the paper.

### 2.2 Minimax lower bound

One of the major theoretical contributions of this paper is to study the estimation error of the mean matrix $\left(\mu^{*}\right){\left(L^{*}\right)}^{T}$ in the proposed sparse Gaussian mixture model. This differs from most existing minimax results in the clustering literature, which predominantly focus on evaluating the mis-clustering error. As the first step towards the complete theory, we establish the minimax lower bound. Formally, consider the following parameter space

$$\Theta_{K}^{*}=\left\{(\mu ,L,\Sigma ):\mu \in R^{p\times K},L\in {ℒ}_{K},|\text{supp}(\mu )|\leq s,{\left\|\mu L^{T}\right\|}_{F}^{2}=O(sn),\right.\left.\Sigma ≻0,0<m_{\Sigma }\leq \lambda_{\text{min}}(\Sigma )\leq \lambda_{\text{max}}(\Sigma )\leq M_{\Sigma }<\infty \right\},$$

where ${ℒ}_{K}=\{L\in R^{n\times K}:L={\left[l_{1},\ldots ,l_{n}\right]}^{T},l_{i}\in \{0,1{\}}^{K},{\left\|l_{i}\right\|}_{0}=1\;\text{for}\;\text{all}\;i\in [n]\}$ is the set of cluster assignment matrices, supp(μ) is the set of indices of the non-zero rows of μ, and $\lambda_{\text{min}}(\Sigma )$ and $\lambda_{\text{max}}(\Sigma )$ represents the smallest and largest singular value of Σ respectively. We also denote $\Delta ={\text{min}}_{k_{1}\neq k_{2}}{\left\|\mu_{k_{1}}^{*}-\mu_{k_{2}}^{*}\right\|}_{2}$ as the minimum separation of the cluster centers.

We next present a collection of assumptions that are necessary in theoretical analyses.

**Assumption 1** *(Low rank)* Klogn≲logp,K≤s.

**Assumption 2** *(Minimum separation)* $\Delta \geq 1/n^{q}$ *for some constant* q>0.

**Assumption 3** *(High dimensionality)* p/n→∞.

Assumption 1 is a mild low-rank assumption and can be satisfied even with increasing K. Assumption 2 requires that the centers of different clusters are well separated and is common in high-dimensional clustering problems. It also guarantees the identifiability of (μ,L) up to a permutation. Assumption 3 requires p/n→∞ and it describes the high-dimensional nature of the problem. Below, Theorem 1 establishes the minimax lower bound for estimating the mean matrix with regard to the Frobenius norm.

**Theorem 1** *Let* $Y=\left(\mu^{*}\right){\left(L^{*}\right)}^{T}+E$ *where each column of* E *is normal random vector with mean zero and covariance matrix* $\Sigma^{*}$. *Assume Assumptions 1–3 hold. Then there exists a constant* C>0 *such that*

$$\underset{\hat{\mu },\hat{L}}{\text{inf}}\underset{\left(\mu^{*},L^{*},\Sigma^{*}\right)\in \Theta_{K}^{*}}{\text{sup}}E_{*}\left\{{\left\|\hat{\mu }{\hat{L}}^{T}-\left(\mu^{*}\right){\left(L^{*}\right)}^{T}\right\|}_{F}^{2}\right\}\geq C(s\;\text{log}\;p+n\;\text{log}\;K)$$

*for sufficiently large* n, *where* $E_{*}$ *denotes the expected value with respect to* $\left(\mu^{*},L^{*},\Sigma^{*}\right)$.

The key challenge in the proof of Theorem 1 lies in designing suitable subsets of the parameter space for (μ,L). We construct three parameter subspaces, each essentially fixing $(\mu {)}_{S},S$, and L, respectively. By controlling the Kullback–Leibler diameter and entropy of each subspace, we apply Fano’s lemma to derive minimax lower bounds on the convergence rate in each subspace, integrating them to obtain the final minimax lower bound.

The minimax lower bound consists of two parts: slogp and nlogK. The slogp term describes the logarithmic complexity of selecting s non-zero coordinates among p variables. It appears repeatedly in the minimax rates for high-dimensional problems where sparsity plays an important role, including the sparse normal means problem (Castillo and van der Vaart, 2012) and the sparse linear regression (Castillo et al., 2015). The term nlogK comes from the logarithmic complexity of assigning n points into K clusters and also appears in the minimax risk for parameter estimation in stochastic block models (Ghosh et al., 2020).

**Remark 2** *This result fills the gap in the literature of high dimensional low-rank matrix estimation, particularly in scenarios where both sparsity and clustering structures exist. When* Y *is a* p×n *random matrix that can be written as* Y=X+E, *where* E *is a* p×n *noise matrix whose entries are independent standard normal random variables and* X *is a* p×n *rank-K matrix,* Yang et al. (2016) *showed that, if* X *not only is low rank but also has only an* s×l *non-zero submatrix, then the minimax lower bound is* $K\left(s+l\right)+s\;\text{log}\;(ep/s)+l\;\text{log}\;(en/l)$. *Our minimax lower bound is sharper than the above bound because the right singular subspace induced by* L *contains a clustering structure, whereas the matrix* X *considered in* Yang et al. (2016) *does not have a structured right singular subspace*.

### 2.3 Minimax upper bound and a constrained maximum likelihood estimator

From the frequentist perspective, an ideal method for parameter estimation in a well-specified statistical model is the maximum likelihood estimator (MLE). In this subsection, we propose a constrained MLE for estimating the mean matrix $\left(\mu^{*}\right){\left(L^{*}\right)}^{T}$. We prove that the risk bound of this estimator achieves the minimax lower bound, thereby showing that the minimax lower bound coincides with the minimax risk modulus a multiplicative constant.

Assuming the number of clusters K is known, we consider the parameter space $\Theta_{K}=\left\{(\mu ,L):\mu \in R^{p\times K},L\in {ℒ}_{K},|\text{supp}(\mu )|\leq s\right\}$ and define the following constrained MLE

(2) $$(\hat{\mu },\hat{L})=\underset{(\mu ,L)\in \Theta_{K}}{\text{arg}\;\text{min}}{\left\|Y-\mu L^{T}\right\|}_{F}^{2}.$$

It is worth noting that the parameter space in the constrained MLE is not necessarily compact. However, by characterizing a compact neighbor of $\left(\mu^{*}\right){\left(L^{*}\right)}^{T}$ and controlling the complexity inside and outside this neighbor separately, we can establish the risk bound of the constrained MLE and show that it achieves the minimax lower bound in Theorem 1.

**Theorem 3** *Suppose that* $\left(\mu^{*},L^{*},\Sigma^{*}\right)\in \Theta_{K}^{*}$ *and* $\left(\hat{\mu },\hat{L}\right)$ *is defined as in* (2) *and Assumptions 1–3 hold. Then there exists some constant* c>0 *such that*

$$\underset{\left(\mu^{*},L^{*},\Sigma^{*}\right)\in \Theta_{K}^{*}}{\text{sup}}E_{*}\left\{{\left\|\left(\mu^{*}\right){\left(L^{*}\right)}^{T}-\hat{\mu }{\hat{L}}^{T}\right\|}_{F}^{2}\right\}\leq c\;(n\;\text{log}\;K+s\;\text{log}\;p),$$

*where* $E_{*}$ *denotes the expected value with respect to* $\left(\mu^{*},L^{*},\Sigma^{*}\right)$.

The proof of Theorem 3 relies on transforming the upper bound of the convergence rate into an empirical process on ${\tilde{\Theta }}_{K}$, which is the normalization of the feasible set of the estimator $\Theta_{K}$. This poses challenges due to the infinite entropy of $\Theta_{K}$. To overcome this, we decompose ${\tilde{\Theta }}_{K}$ into layers ${\left({ℰ}_{j}^{K}\right)}_{j\in Z}$ with manageable entropy. We classify these layers into three scenarios: small volume, large volume, and intermediate volume. For the small volume case, we establish that L equals $L^{*}$ up to a permutation within ${ℰ}_{j}^{K}$, simplifying the complexity to focus on μ alone. In the large volume case, we control the entropy separately for the spaces of μ and L. Lastly, in the intermediate volume case, we leverage the ellipsoidal nature of ${ℰ}_{j}^{K}$ and its packing to bound the covering numbers efficiently.

Despite the theoretical optimality of the constrained MLE, it is computationally intractable in general since the feasible set $\Theta_{K}$ is nonconvex and involves discrete structures. In addition, the implementation of the constrained MLE requires to pre-specify the sparsity level s and the number of clusters K, which are usually unknown in practice. These computational challenges motivate us to develop a Bayesian method that can be implemented conveniently using an MCMC sampler without specifying s and K *a priori*.

### 2.4 Bayesian sparse high-dimensional Gaussian mixture model

As described in the previous subsection, the optimization-based constrained MLE is computationally intractable due to the non-convexity and discrete structure of the problem. One may apply the EM algorithm, which iterates between a clustering step given the recently updated parameter values and a parameter estimation step given the recently updated cluster memberships, to address this issue. For example, Cai et al. (2019) proposed an approach that estimates the sparse discriminant vector and obtains the clustering memberships in the Expectation step to avoid the singularities of sample covariance matrices in high dimensions. Another approach is spectral clustering (Luxburg, 2004). However, the optimality of spectral clustering is only established when p=o(nΔ) without sparsity structure (Löffler et al., 2021). In this subsection, we propose a Bayesian approach to estimate the high-dimensional sparse Gaussian mixture model. As will be seen later, the proposed Bayesian method has a minimax-optimal posterior contraction rate.

We deliberately consider a misspecified sampling model: $Y_{i}=\mu_{z_{i}}+\epsilon_{i}$, where the error term $\epsilon_{i}$ follows a multivariate normal distribution with mean vector zero and identity covariance matrix $I_{p}$. This intentional simplification is motivated by theoretical convenience, since our primary focus lies in the mean matrix $\mu L^{T}$. As we will prove later, as long as the spectrum of the true covariance matrix $\Sigma^{*}$ is bounded, the posterior distribution of μL concentrates on the true parameter $\left(\mu^{*}\right){\left(L^{*}\right)}^{T}$ at a minimax-optimal rate.

To promote sparsity, we use the spike-and-slab LASSO prior (Ročková and George, 2018) for the mean vectors of clusters. The spike-and-slab LASSO prior can be viewed as a continuous relaxation of the spike-and-slab prior (Mitchell and Beauchamp, 1988), which is a mixture of a point mass at zero (referred to as the “spike” distribution) and an absolutely continuous distribution (referred to as the “slab” distribution). Formally, for $x\in R^{p}$, the spike-and-slab LASSO prior is defined as follows: for j∈[p], $\pi \left(x_{j}|\lambda_{0},\lambda_{1},\xi_{j}\right)=(1-\left.\xi_{j}\right)\psi \left(x_{j}|\lambda_{0}\right)+\xi_{j}\psi \left(x_{j}|\lambda_{1}\right)$ and $\left(\xi_{j}|\theta \right)\sim \text{Bernoulli}(\theta )$, where ψ(x∣λ)=(λ/2)exp(-λ|x|) is the density of Laplace distribution with mean 0 and variance $2/\lambda^{2}$. By assuming $\lambda_{0}\gg \lambda_{1},\psi \left(x_{j}|\lambda_{0}\right)$ closely resembles the “spike” distribution in the spike-and-slab prior since it is highly concentrated at 0, whereas $\psi \left(x_{j}|\lambda_{1}\right)$ plays the role of the “slab” distribution. We follow the notation in Ročková and George (2018) and use $\text{SSL}\left(\lambda_{0},\lambda_{1},\theta \right)$ to denote this prior model. In the context of our proposed sparse Gaussian mixture model, we define the $\text{joint-SSL}\left(\lambda_{0},\lambda_{1},\theta \right)$ as follows to further incorporate the case where the mean vectors $\mu_{1},\ldots ,\mu_{K}\in R^{p}$ share the same sparsity pattern: given K, for j∈[p],

$$\pi \left(\mu_{1j},\cdots ,\mu_{Kj}|\lambda_{0},\lambda_{1},\xi_{j}\right)=\prod_{k=1}^{K}\left(\left(1-\xi_{j}\right)\psi \left(\mu_{kj}|\lambda_{0}\right)+\xi_{j}\psi \left(\mu_{kj}|\lambda_{1}\right)\right),\left(\xi_{j}|\theta \right)\sim \text{Bernoulli}\left(\theta \right).$$

Under this prior distribution, the random vectors $\mu_{1},\ldots ,\mu_{K}$ are conditionally independent given K and a sparsity indicator vector $\xi \in \{0,1{\}}^{p}$ which controls the common sparsity structure. We further assume that $\theta \sim \text{Beta}\left(1,\beta_{\theta }\right)$, where $\beta_{\theta }=p^{1+\kappa }\;\text{log}\;p$ for some constant κ>0. The choice of the hyperparameter $\beta_{\theta }$ is selected for technical reasons.

We now specify the sparse Gaussian mixture model. Given K, for cluster membership indicators $z_{1},\ldots ,z_{n}$, we assign a categorical prior with a K-dimensional probability vector $w={\left(w_{1},\ldots ,w_{K}\right)}^{T}$, whose hyperprior distribution is a K-dimensional symmetric Dirichlet distribution with the shape parameter α>0. We assign a joint-SSL prior for the mean vectors ${\left\{\mu_{k}\right\}}_{k=1}^{K}$ to adapt to the joint sparsity. To allow for an unknown K, we further assign a truncated Poisson distribution to K by letting $\pi (K)\propto e^{-\lambda }\lambda^{K}/K!$, $K\in \left[K_{\text{max}}\right]$, where $K_{\text{max}}$ is a conservative upper bound for K and should be large enough in practice. Thus, the proposed Bayesian sparse Gaussian mixture model can be expressed as follows:

(3) $$\left(Y_{1},\ldots ,Y_{n}|z,\mu \right)\sim N_{p}\left(\mu_{k},I_{p}\right)\;\text{independently},$$

(4) $$\left(\mu_{1},\ldots ,\mu_{K}|K,\theta \right)\sim \text{joint-SSL}\left(\lambda_{0},\lambda_{1},\theta \right),$$

(5) $$\left(z_{1},\ldots ,z_{n}|w,K\right)\sim \text{Categorical}(w)\;\text{independently},$$

(6) $$(w|K)\sim {\text{Dirichlet}}_{K}(\alpha ),$$

(7) $$\pi \left(K\right)\propto \frac{e^{-\lambda }\lambda^{k}}{k!},\;K\in \left[K_{\text{max}}\right]$$

(8) $$\theta \sim \text{Beta}\left(1,\beta_{\theta }\right)\;\text{where}\;\beta_{\theta }=p^{1+\kappa }\;\text{log}\;p.$$

The use of sparsity-enforcing priors in Gaussian mixture models has been widely explored in Bayesian literature. For example, Tadesse et al. (2005) and Gao et al. (2020) proposed a discrete subset-selection prior for clustering high-dimensional data. In this paper, we employ a continuous spike-and-slab shrinkage prior. While Tadesse et al. (2005) focused solely on computational algorithms without theoretical analysis, and Gao et al. (2020) primarily investigated the theoretical results of parameter estimation, our main contribution lies in establishing the theoretical properties of the proposed model concerning both parameter estimation and mis-clustering error. This represents the first effort in developing a general theoretical framework for Bayesian analyses of high-dimensional clustering.

## 3. Theoretical Properties

In this section, we establish the theoretical guarantees of the proposed Bayesian sparse Gaussian mixture model.

### 3.1 Posterior contraction rate

In this subsection, we show that the posterior contraction rate with respect to the Frobenius norm metric is minimax optimal under the propose Bayesian sparse Gaussian mixture model. All the proofs are deferred to the Appendix.

By the Bayes formula, the posterior distribution of μ and L can be written as

$$\Pi \{(\mu ,L)\in ℰ|Y)=\frac{\int_{ℰ}p_{n}(Y|\mu ,L)/p_{n}\left(Y|\mu^{*},L^{*}\right)\Pi (d\mu dL)}{\int_{\Theta }p_{n}(Y|\mu ,L)/p_{n}\left(Y|\mu^{*},L^{*}\right)\Pi (d\mu dL)},$$

where $p_{n}(Y|\mu ,L)=(2\pi {)}^{-np/2}\text{exp}\left(-{\left\|Y-\mu L^{T}\right\|}_{F}^{2}/2\right)$ is the likelihood of the data matrix Y with identity covariance matrix and ℰ is any measurable subset of $\Theta =\overset{K_{\text{max}}}{\underset{K=1}{⋃}}R^{p\times K}\times {ℒ}_{K}$. In Theorem 4, we derive the posterior contraction rate under the proposed Bayesian model.

**Theorem 4** *Let* Y *be generated from a mixture of* $K^{*}$ *Gaussian distributionsas in* (1) *with the true mean vectors* $\mu^{*}=\left[\mu_{1}^{*},\ldots ,\mu_{K^{*}}^{*}\right]$ *and the true cluster membership matrix* $L^{*}$, *where* $\left|\text{supp}\left(\mu^{*}\right)\right|\leq s$. *Suppose Assumptions 1 – 3 hold. Let* μ *and* L *follow the prior specification in* (3)–(8) *with some hyperparameters* $\kappa >0,\alpha \geq 1,\lambda_{0}\geq 2\;\text{log}(p/s)\sqrt{np/(s\;\text{log}\;p)}$ *and* $1/n^{\gamma }≲\lambda_{1}≲\sqrt{s\;\text{log}\;p/{\left\|\mu^{*}\right\|}_{F}}$ *for some constants* γ>0. *Then, we have*

$$\Pi \left\{(\mu ,L):{\left\|\mu L^{T}-\left(\mu^{*}\right){\left(L^{*}\right)}^{T}\right\|}_{F}^{2}\geq M\left(s\;\text{log}\;p+n\;\text{log}\;K_{\text{max}}\right)|Y\right\}\to 0$$

*in* $P_{\left(\mu^{*},L^{*},\Sigma^{*}\right)}$- *probability, for every large constant* M *and* $\left(\mu^{*},L^{*},\Sigma^{*}\right)\in \Theta_{K}^{*}$.

The proof of Theorem 4 adopts a modified “testing-and-prior-concentration” approach (Ghosal et al., 2000), tailored to address the unique challenges arising from model misspecification in our setting. We rigorously prove the three conditions: (1) The prior distribution puts a sufficient mass on the neighbourhood of the true parameter $\left(\mu^{*}\right){\left(L^{*}\right)}^{T}$; (2) There exists a test function which can distinguish $\left(\mu^{*}\right){\left(L^{*}\right)}^{T}$ from the complement of its neighbourhood in a subset of the parameter space; (3) The prior puts almost all mass on the subset of parameter space in condition (2).

**Remark 5** *Recall from* Section 2 *that the minimax lower bound contains the true number of clusters* $K^{*}$, *which is unknown in many applications. The posterior contraction rate obtained in Theorem 4 contains a logarithmic factor of the upper bound* $K_{\text{max}}$ *for* $K^{*}$. *If we further assume that* $K_{\text{max}}≍{\left(K^{*}\right)}^{q}$ *for some constant* q≥1, *the posterior contraction rate matches the minimax lower bound in Theorem 1 and is optimal thereafter. For* $\lambda_{1}$ *in the joint-SSL prior, if we further assume* ${\left\|\mu_{k}^{*}\right\|}_{2}^{2}=O\left(s\right)$ *for any* $k\in \left[K^{*}\right]$, *then the upper bound of* $\lambda_{1}$ *can be relaxed to* $\lambda_{1}≲\sqrt{\text{log}\;p/K_{\text{max}}}$, *which is a mild condition and can be easily satisfied in practice*.

We assume that the cluster mean vectors are jointly sparse. However, Theorem 4 can be easily generalized to the case where the cluster centers do not share the common sparsity. Specifically, each mean vector $\mu_{k}$ has at most s non-zero coordinates but the indices of the non-zero coordinates are not necessarily the same across k∈[K]. Clearly, the matrix $\mu =\left[\mu_{1},\ldots ,\mu_{K}\right]$ is jointly Ks-sparse. To adapt to the column-wise sparsity of μ, we modify the prior model by letting $\mu_{1},\ldots ,\mu_{K}$ follow the SSL prior independently given K:

(6’) $$\left(\mu_{k}|K,\theta \right)\sim \text{SSL}\left(\lambda_{0},\lambda_{1},\theta \right)\;\text{for}\;k=1,\ldots ,K.$$

The following corollary gives the posterior contraction rate under such a modification.

**Corollary 6** *Let* Y *be generated from a mixture of* $K^{*}$ *Gaussian distributions as in* (1) *with the true mean vectors* $\mu^{*}={\left[\mu_{1}^{*},\ldots ,\mu_{K^{*}}^{*}\right]}^{T}$ *and the true cluster membership matrix* $L^{*}$, *where* $\left|\text{supp}\left(\mu^{*}\right)\right|\leq K^{*}s$. *Suppose Assumptions 1–3 hold. Let* μ *and* L *follow the prior specification in* (3), (6’), (5)–(8) *with the same hyperparameters as in Theorem 4. Then, we have*

$$\Pi \left\{(\mu ,L):{\left\|\mu L^{T}-\left(\mu^{*}\right){\left(L^{*}\right)}^{T}\right\|}_{F}^{2}\geq M\left(sK_{\text{max}}\;\text{log}\;p+n\;\text{log}\;K_{\text{max}}\right)|Y\right\}\to 0$$

*in* $P_{\left(\mu^{*},L^{*},\Sigma^{*}\right)}$- *probability, for every large constant* M *and* $\left(\mu^{*},L^{*},\Sigma^{*}\right)\in \Theta_{K}^{*}$.

**Remark 7** *Corollary 6 can be easily extended to the case when the mean vectors have not only different sparsity structures, but also distinct sparsity sizes, i.e., $\left|\text{supp}\left(\mu_{k}\right)\right|\neq \left|\text{supp}\left(\mu_{k^{\prime }}\right)\right|$* *for some* $k\neq k^{\prime }$. In such a case, the first term of the posterior contraction rate becomes to $\sum_{k=1}^{K^{*}}\left|\text{supp}\left(\mu_{k}\right)\right|K_{\text{max}}\;\text{log}\;p$.

### 3.2 Mis-clustering error

Recovering cluster memberships is always a focal objective for clustering problems. In this subsection, we obtain a mis-clustering error bound of the proposed Bayesian model *a posteriori* based on the posterior contraction result for parameter estimation in Theorem 4. For any two cluster membership vectors $z,z^{\prime }\in ([K]{)}^{n}$, define the minimum Hamming distance $d_{H}\left(z,z^{\prime }\right)=(1/n){\;\text{inf}}_{\tau \in S_{K}}\sum_{i=1}^{n}1\left\{z_{i}\neq \tau \left(z_{i}^{\prime }\right)\right\}$ as the mis-clustering rate between z and $z^{\prime }$, where $S_{K}$ is the set of all permutations on [K]. Let $\sigma_{\text{max}}(X)$ and $\sigma_{\text{min}}(X)$ denote the largest and smallest non-zero singular value of matrix X, respectively. Below, we obtain the posterior contraction result for the mis-clustering error measured by $d_{H}$.

**Theorem 8** Assume the conditions in Theorem 4 hold and $n_{k}^{*}=\sum_{i=1}^{n}1\left(z_{i}^{*}=k\right)\to \infty$ *for all* k. *Let* $n_{\text{min}}^{*}≜{\text{min}}_{k\in \left[K\right]}n_{k}^{*}$ *and* $n_{\text{max}}^{*}≜{\text{max}}_{k\in \left[K\right]}n_{k}^{*}$. *Then we have*

$$\Pi \left\{\left.nd_{H}\left(z,z^{*}\right)\geq \frac{M{\left(n_{\text{max}}^{*}\right)}^{3}\sigma_{\text{max}}{\left(\mu^{*}\right)}^{2}}{{\left(n_{\text{min}}^{*}\right)}^{4}\sigma_{\text{min}}{\left(\mu^{*}\right)}^{4}}\left(s\;\text{log}\;p+n\;\text{log}\;K_{\text{max}}\right)\right|Y\right\}\to 0$$

*in* $P_{\left(\mu^{*},L^{*},\Sigma^{*}\right)}$- *probability for every large constant* M.

The main challenge in proving Theorem 8 is translating clustering accuracy into errors regarding the right singular subspace of $\mu L^{T}$. By leveraging a variant of the Davis-Kahan Theorem, we bound the distance of the right singular subspace of $\mu L^{T}$ using the distance of $\mu L^{T}$. Through geometric analysis, we show that correct clustering is achieved if the errors of right singular vectors are sufficiently small. This allows us to control mis-clustering error by the error of $\mu L^{T}$, and the desired result follows by directly applying Theorem 4.

**Remark 9** *If we assume* $(s\;\text{log}\;p)/\left(n{\left\|\mu^{*}\right\|}_{F}^{2}\right)\to 0$, *then by Theorem 8, the proportion of the mis-clustered data points is asymptotically negligible with a high posterior probability provided that* ${\left(n_{\text{max}}^{*}\right)}^{3}\sigma_{\text{max}}{\left(\mu^{*}\right)}^{4}=O\left({\left(n_{\text{min}}^{*}\right)}^{4}\sigma_{\text{min}}{\left(\mu^{*}\right)}^{4}\right)$ *as* n→∞. *Moreover, if we further assume that* $\sigma_{\text{max}}\left(\mu^{*}\right)≲\sigma_{\text{min}}\left(\mu^{*}\right)$ *and* $n_{\text{max}}^{*}≲n_{\text{min}}^{*}=O(n)$ *(which means that the sizes of the smallest cluster and the largest cluster are of the same order as* n*), then the number of mis-clustered data points, i.e.,* $nd_{H}\left(z,z^{*}\right)$, *is asymptotically bounded by a constant with a high posterior probability because* ${\left\|\mu^{*}\right\|}_{F}^{2}≍\sigma_{\text{min}}{\left(\mu^{*}\right)}^{2}$ *in this case*.

**Remark 10** Azizyan et al. (2013) *and* Cai et al. (2019) *also studied high-dimensional clustering with the sparsity assumption. However, they only considered the case when the number of clusters was 2. Assuming that* $\mu_{1}^{*}-\mu_{2}^{*}$ *was sparse,* Azizyan et al. (2013) *showed that the minimax optimal convergence rate of mis-clustering was* $\sqrt{s\;\text{log}\;p/n}/\Delta^{2}$ *when the two clusters had same mixing weights and isotropic covariance matrices. Assuming that the discriminant direction vector* $\beta^{*}={\left(\Sigma^{*}\right)}^{-1}\left(\mu_{1}^{*}-\mu_{2}^{*}\right)$ *was sparse,* Cai et al. (2019) *showed that the convergence rate of the excess mis-clustering error, defined as the difference between the mis-clustering error and the optimal mis-classification error obtained by Fisher’s linear discriminant rule when cluster-specific parameters were known, achieved the minimax optimal rate of* slogp/n. *However, the convergence rate of mis-clustering error was not investigated. In addition,* Li et al. (2017), Azizyan et al. (2013) *and* Cai et al. (2019) *focused on the two-cluster problem, but the minimax optimal result for high-dimensional sparse clustering with* K>2 *clusters was not studied. In contrast, we allow* K *to grow moderately with the sample size* n.

## 4. Simulation Studies

We evaluate the empirical performance of the proposed Bayesian method for sparse Gaussian mixtures through analyses of synthetic data sets. Posterior inference is carried out through an MCMC sampler, the details of which are provided in Appendix C. We also compare the performance of our model with four competitors: principal component analysis K-means (PCA-KM), sparse K-means (SKM) (Witten and Tibshirani, 2010), Clustering of High-dimensional Gaussian Mixtures with the EM (CHIME), and Gaussian-mixture-model-based clustering (MClust) (Fraley et al., 2012). In particular, PCA-KM is a two-stage approach that first performs a PCA to reduce dimensionality and then applies a K-means algorithm to the principal components. SKM is a generalization of the K-means in high dimensions to find clusters and important features (i.e., the non-zero coordinates) simultaneously. CHIME is a high-dimensional clustering approach based on an EM algorithm. To overcome the issue that the sample covariance matrix may not be invertible and thus the subsequent estimate of z is not available, CHIME focuses on the so-called sparse discriminant vector and directly use it in the Fisher discriminant rule to estimate cluster memberships. Note that the performance of CHIME is quite sensitive to the choice of initial values. Throughout simulation examples in this section, we set the initial values of CHIME to be the output of K-means. For PCA-KM and SKM, we choose the number of clusters via Silhouette method (Rousseeuw, 1987), with the range of K being from 2 to 10. For MClust and CHIME, the number of clusters is estimated via Bayesian information criterion (BIC).

### 4.1 Simulation setup

We consider three simulation scenarios. Scenario I is designed to evaluate the proposed Bayesian method in terms of clustering accuracy with varying numbers of clusters and support sizes of the mean vectors. The data matrix Y is of size p×n with p=400 and n=200. We assume that the true number of clusters $K^{*}$ ranges over {3, 5} and the support size s ranges over {6, 12}. We use 𝒮 to denote the set of non-zero coordinates and let the first s coordinates of the cluster means be non-zero, i.e., 𝒮={1,2,…,s}. For each $K^{*}\in \{3,5\}$, the true cluster assignment $z_{i}^{*}$ is generated from a categorical distribution: $z_{i}^{*}\sim \text{Cat}\left(p_{K^{*}}\right)$, where $p_{3}=(0.3,0.3,0.4)$ and $p_{5}=(0.2,0.2,0.2,0.2,0.2)$. When $K^{*}=3$, the three cluster mean vectors are ${\left(\mu_{1}^{*}\right)}_{𝒮}=3\times (1,1,\ldots ,1{)}^{T},{\left(\mu_{2}^{*}\right)}_{𝒮}=-1.5\times (1,1,\ldots ,1{)}^{T}$ and ${\left(\mu_{3}^{*}\right)}_{𝒮}=(0,\ldots ,0{)}^{T}$, where ${\left(\mu_{1}^{*}\right)}_{𝒮},{\left(\mu_{2}^{*}\right)}_{𝒮},{\left(\mu_{3}^{*}\right)}_{𝒮}\in R^{s}$. For $K^{*}=5$, the five cluster mean vectors are ${\left(\mu_{1}^{*}\right)}_{𝒮}=4\times (1,1,\ldots ,1{)}^{T},{\left(\mu_{2}^{*}\right)}_{𝒮}=-4\times (1,1,\ldots ,1{)}^{T},{\left(\mu_{3}^{*}\right)}_{𝒮}=(0,\ldots ,0{)}^{T},{\left(\mu_{4}^{*}\right)}_{𝒮}=4\times (-1,1,-1,1,\ldots ,-1,1{)}^{T}$ and ${\left(\mu_{5}^{*}\right)}_{𝒮}=1.5\times (1,-1,1,-1,\ldots ,1,-1{)}^{T}$. Given $K^{*}$ and $z_{i}^{*}$’s, data are generated from $Y_{i}\sim N\left(\mu_{z_{i}^{*}}^{*},I_{p}\right)$.

Scenario II focuses on the case when small clusters exist. The data matrix Y consists of n=200 observations with dimension p=400. We assume that the true number of clusters $K^{*}=3$ and the support size s=8. Similarly as Scenario I, we set 𝒮={1,2,…,s}. The mean vectors over the support 𝒮 in the three clusters are ${\left(\mu_{1}^{*}\right)}_{𝒮}=(5,2,\cdots ,5,2{)}^{T},{\left(\mu_{2}^{*}\right)}_{𝒮}=(10,5,\cdots ,10,5{)}^{T}$, and ${\left(\mu_{3}^{*}\right)}_{𝒮}=(15,2,\cdots ,15,2{)}^{T}$, respectively. For each observation i, its simulated true cluster assignment $z_{i}^{*}$ is generated from a categorical distribution independently: $z_{i}^{*}\sim \text{Cat}(0.02,0.48,0.5)$. Given $K^{*}$ and $z_{i}^{*}$’s, data are generated from $Y_{i}\sim N\left(\mu_{z_{i}^{*}}^{*},\Sigma_{z_{i}^{*}}\right)$, where $\Sigma_{1}=\Sigma_{3}=I_{p}$, and $\Sigma_{2}$ is a diagonal matrix whose diagonal entries equal 4 on the coordinates in the support 𝒮 and 1 elsewhere.

Scenario III aims to investigate the robustness of the proposed Bayesian method to the misspecification of the sampling distribution. The true distribution of the data is assumed to be a mixture of multivariate t distributions but we use the Gaussian mixtures as the working likelihood. The data matrix Y consists of n=200 observations of multivariate t-mixtures with dimension p=400 and a degree of freedom 5. The number of clusters is set to $K^{*}=3$, and the first s=8 coordinates of cluster mean vectors are non-zero. We generate cluster assignments $z_{i}^{*}$’s from a categorical distribution: $z_{i}^{*}\sim \text{Cat}(0.2,0.4,0.4)$ independently for i∈[n], and let the cluster mean vectors $\mu_{1}^{*},\mu_{2}^{*},\mu_{3}^{*}$ and the covariance matrices $\Sigma_{1},\Sigma_{2},\Sigma_{3}$ be the same as those in Scenario II for each multivariate t-cluster.

For each of the three scenarios, we apply the proposed Bayesian sparse Gaussian mixture model to the simulated data with 100 repeated simulations. In each simulation, we compute posterior inference using the developed MCMC sampler with 1000 burn-in iterations and another 4000 iterations for post-burn-in samples. The upper bound of the number of clusters is set to be $K_{\text{max}}=20$. We set the hyperparameters $\kappa ,\lambda_{0}$, and $\lambda_{1}$ in the spike-and-slab LASSO prior to be 0.1, 100, and 1 respectively, and λ in the truncated Poisson prior for K to be 2. The estimated number of clusters and cluster assignments under the proposed Bayesian method are reported based on the posterior mode of $z_{i}$’s from post-burn-in MCMC samples. The proposed Bayesian method, Mclust, PCA-KM, and SKM are performed under R with version 4.2.1 and CHIME is performed under Matlab with version 9.11 (R2021b).

### 4.2 Simulation results

**Figure 1:**
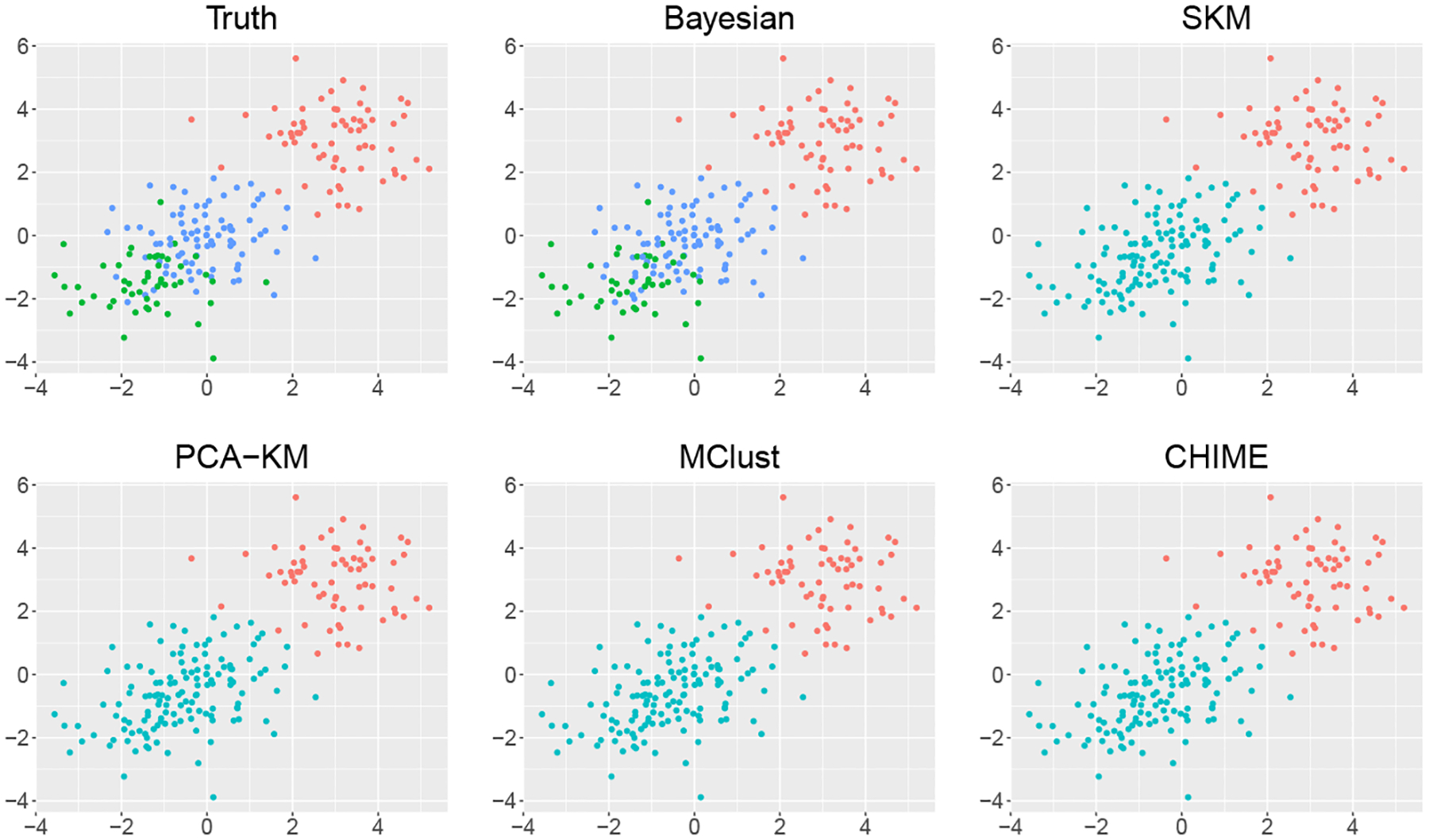
Clustering results of different methods compared to the true cluster memberships in Scenario I with $K^{*}=3$ and s=6 in a randomly selected simulation replicate. Data points are projected onto the subspace of the first two coordinates and different colors correspond to different estimated cluster memberships.

We first investigate the performance of the proposed Bayesian sparse Gaussian mixture model against the four competitors in Scenario I. We focus on the following three objectives: identification of the number of clusters, the clustering accuracy, and the cluster-wise mean vector estimation accuracy. The proposed Bayesian method can successfully recover the simulated true number of clusters. Specifically, when $K^{*}=3$, the proposed method identifies 3 clusters in 85 replicates out of 100 replicates for s=6 and in 98 replicates for s=12; when $K^{*}=5$, the proposed method identifies 5 clusters in 83 replicates out of 100 replicates for s=6 and in 98 replicates for s=12. In contrast, all the four competitors underestimate the number of clusters. In particular, when $K^{*}=3$, the estimated number of clusters using the four competitors all equal to 2 in 100 simulation replicates. When $K^{*}=5$, PCA-KM, SKM, MClust, and CHIME only correctly estimate the number of clusters in 6, 0, 4, and 3 out of 100 replicates for s=6, and in 8, 0, 9, and 3 out of 100 replicates for s=12. Figure 1 and Appendix Figure A1 plot the simulated true cluster memberships and the estimated clustering results under the proposed Bayesian method and the four competitors for one randomly selected simulation replicate when $K^{*}=3,s=6$, and $K^{*}=5,s=6$, respectively. We can see that the four competitors cannot well distinguish clusters with a certain degree of overlapping, e.g., the green and blue clusters in the upper left panel of Figure 1, while the proposed Bayesian method can successfully separate them.

In terms of clustering accuracy, we use the adjusted Rand index (ARI) (Rand, 1971) as the evaluation metric. Specifically, let ${𝒞}^{*}$ and ${𝒞}^{†}$ denote the true and estimated partitions of [n] respectively, and let $K^{*}≜\left|{𝒞}^{*}\right|,K^{†}≜\left|{𝒞}^{†}\right|$. Denote $n_{k}^{*}$ the size of kth cluster in ${𝒞}^{*}$ and $n_{k^{\prime }}^{†}$ as the size of $k^{\prime }$th cluster in ${𝒞}^{†}$. Let $n_{k,k^{\prime }}$ be the number of observations that are assigned to both the kth cluster in ${𝒞}^{*}$ and $k^{\prime }$th cluster in ${𝒞}^{†}$. Then the ARI is defined as

ARI𝒞*,𝒞†=∑k=1K*∑k′=1K†nk,k′2-∑k=1K*nk2∑k′=1K†nk′2/n2∑k=1Ktnk2+∑k′=1K†nk′2/2-∑k=1K*nk2∑k′=1K†nk′2/n2.

Table 1 reports the average ARIs of the clustering results of the proposed Bayesian method against the four competitors across 100 simulation replicates under Scenario I. The proposed Bayesian method outperforms the four alternatives in terms of higher ARIs in all settings.

**Figure 2:**
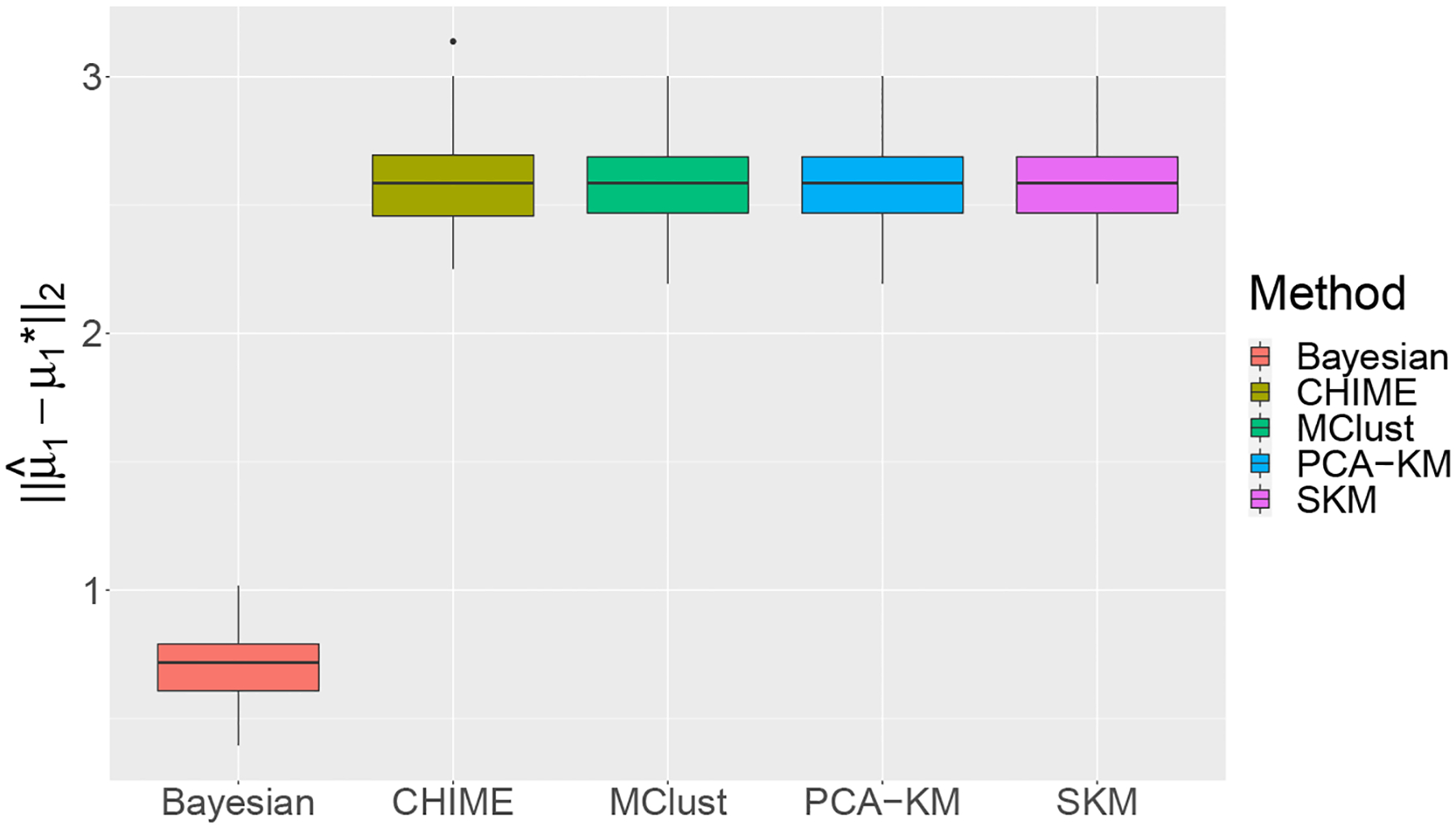
Numerical results of ${\left\|{\overset{ˆ}{\mu }}_{1}-\mu_{1}^{*}\right\|}_{2}$ of different methods in Scenario I with $K^{*}=3$ and s=12 across 100 simulation replicates.

We then examine the cluster-wise mean vector estimation accuracy by computing $\|{\overset{ˆ}{\mu }}_{1}-\mu_{1}^{*}{\|}_{2}$ under the proposed Bayesian method and alternatives, where ${\overset{ˆ}{\mu }}_{1}$ is the estimated mean vector under different methods. Specifically, ${\overset{ˆ}{\mu }}_{1}$ under the proposed Bayesian method is the posterior mean of $\mu_{1}$. CHIME and MClust directly return ${\overset{ˆ}{\mu }}_{1}$ since they are model-based methods. For PCA-KM and SKM, we use the empirical means induced from their estimated clustering memberships as ${\overset{ˆ}{\mu }}_{1}$ since they are based on K-means. Figure 2 presents the boxplots of ${\left\|{\overset{ˆ}{\mu }}_{1}-\mu_{1}^{*}\right\|}_{2}$ when $K^{*}=3$ and s=12 across 100 simulation replicates under different methods, showing that the proposed Bayesian method yields the smallest error of estimating $\mu_{1}^{*}$. Furthermore, Appendix Figure A2 plots the estimated $\mu_{1}^{*}$ under different methods in one randomly selected simulation replicate, indicating that the proposed Bayesian method recovers the sparsity pattern better than the four competitors. Lastly, we report the running times of all methods in Scenario I in Appendix Table A1.

**Figure 3:**
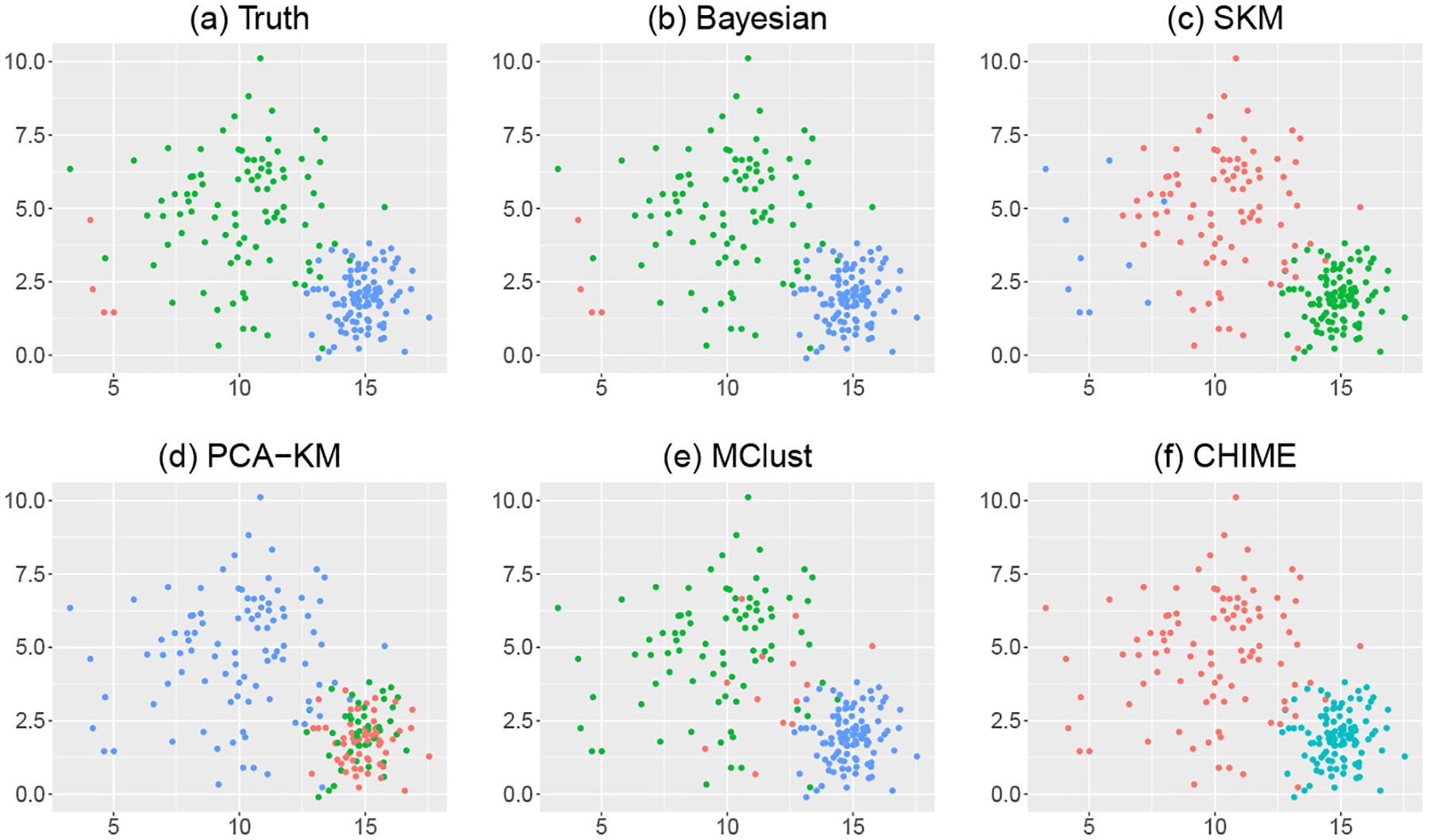
Clustering results of different methods compared with the truth in one randomly selected simulation replicate of Scenario II. (a) Simulated truth. (b) Clustering result under the proposed Bayesian method. (c-f) Clustering results under SKM, PCA-KM, MClust, and CHIME when the number of clusters is fixed to be truth. Data points are projected onto the subspace of the first two coordinates and different colors correspond to different estimated cluster memberships.

Scenario II is designed to evaluate the proposed Bayesian method when small clusters exist. Figure 3(a) shows the true clustering assignments in one randomly selected replicate, in which the small cluster (in red color) only contains four data points. Our Bayesian method successfully discovers the small cluster and yields the estimated number of clusters K=3 in 96 out of 100 simulation replicates, resulting in an average ARI of 0.99. Figure 3(b) shows the estimated clustering memberships under the proposed Bayesian method in the same simulation replicate, exactly matching the truth shown in Figure 3(a). In contrast, all four competitors are not able to identify the small cluster and report the estimated number of clusters K=2 in all 100 simulation replicates. We further examine the performance of the four competitors when we pre-specify the number of clusters to be the truth K=3. Figure 3(c) - (f) plot the estimated clustering memberships obtained from the four competitors. We can see that SKM, PCA-KM, and MClust incline to return clusters with relatively balance sizes, leading to inaccurate clustering assignments with the average ARIs being 0.76, 0.84, and 0.79, respectively, across 100 simulation replicates. CHIME only returns two clusters even though we set the number of clusters to be 3, as shown in Figure 3(f).

**Figure 4:**
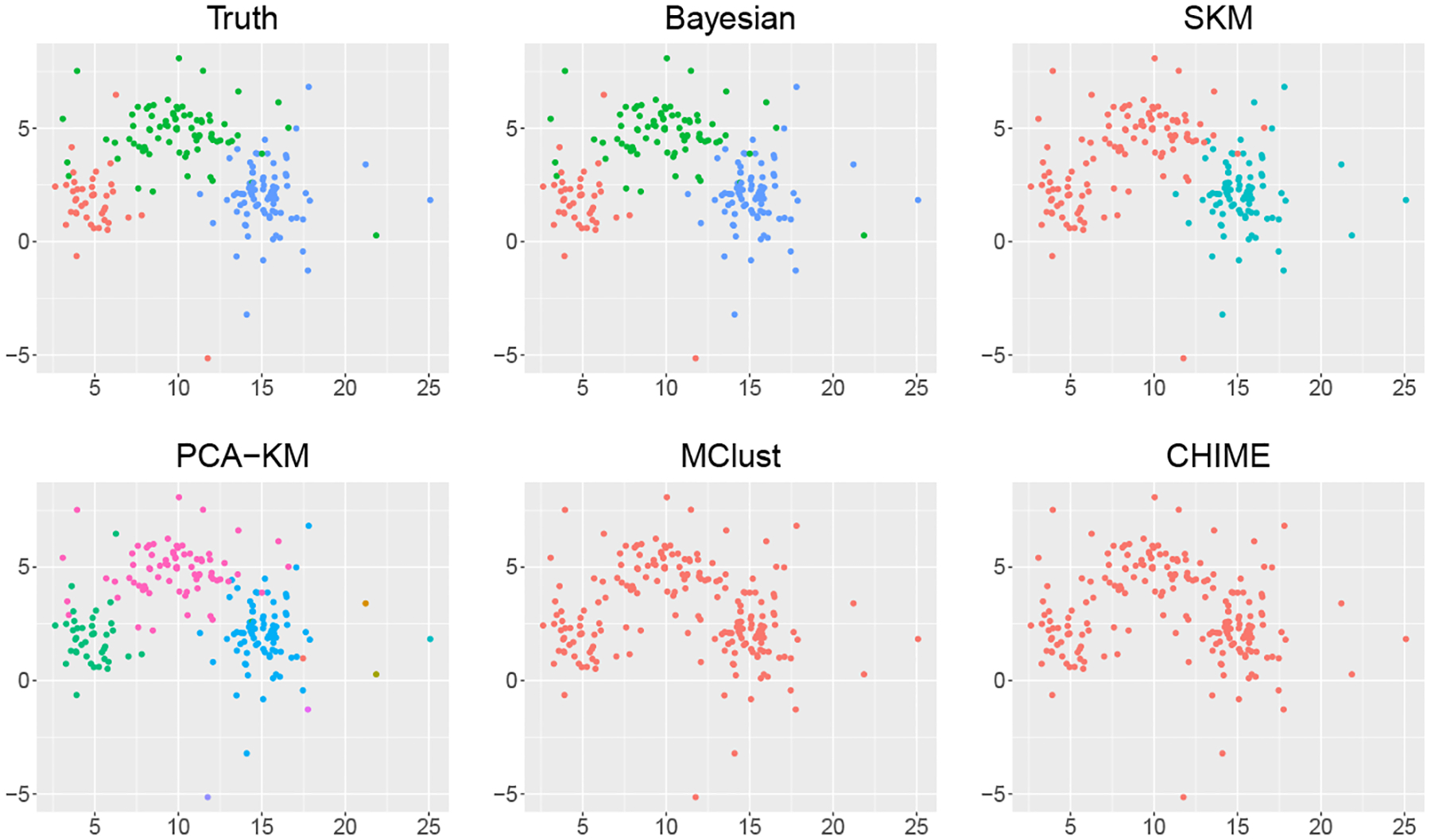
Clustering results under different methods in one randomly selected replicate of Scenario III. Data points are projected onto the subspace of the first two coordinates and different colors correspond to different estimated cluster memberships.

For Scenario III, the histograms of the estimated number of clusters under different methods are presented in Appendix Figure A3. Figure 4 visualizes the clustering results under different methods in a randomly selected simulation replicate. The proposed Bayesian method successfully identifies three clusters in 76 out of 100 simulation replicates, with an average ARI of 0.97 across 100 simulation replicates. Note that the multivariate t-distribution is heavy-tailed. Since we mis-specify the working model as Gaussian mixtures, it is reasonable to treat some observations as “outliers”, as shown in the upper left panel of Figure 4. Therefore, the proposed Bayesian method tends to assign these “outliers” to small clusters when it overestimates the number of clusters. PCA-KM returns 10 clusters in 59 out of 100 simulation replicates with the average ARI being 0.84. The estimated numbers of clusters of SKM are all 2 in 100 simulation replicates, and the average ARI is 0.52. For model-based methods, i.e., MClust and CHIME, which also use Gaussian mixtures as the working likelihood, their performance are much worse than others as they only identify one cluster in 93 out of 100 replicates, resulting in the average ARIs less than 0.05.

## 5. Single-cell Sequencing Data Analysis

**Figure 5:**
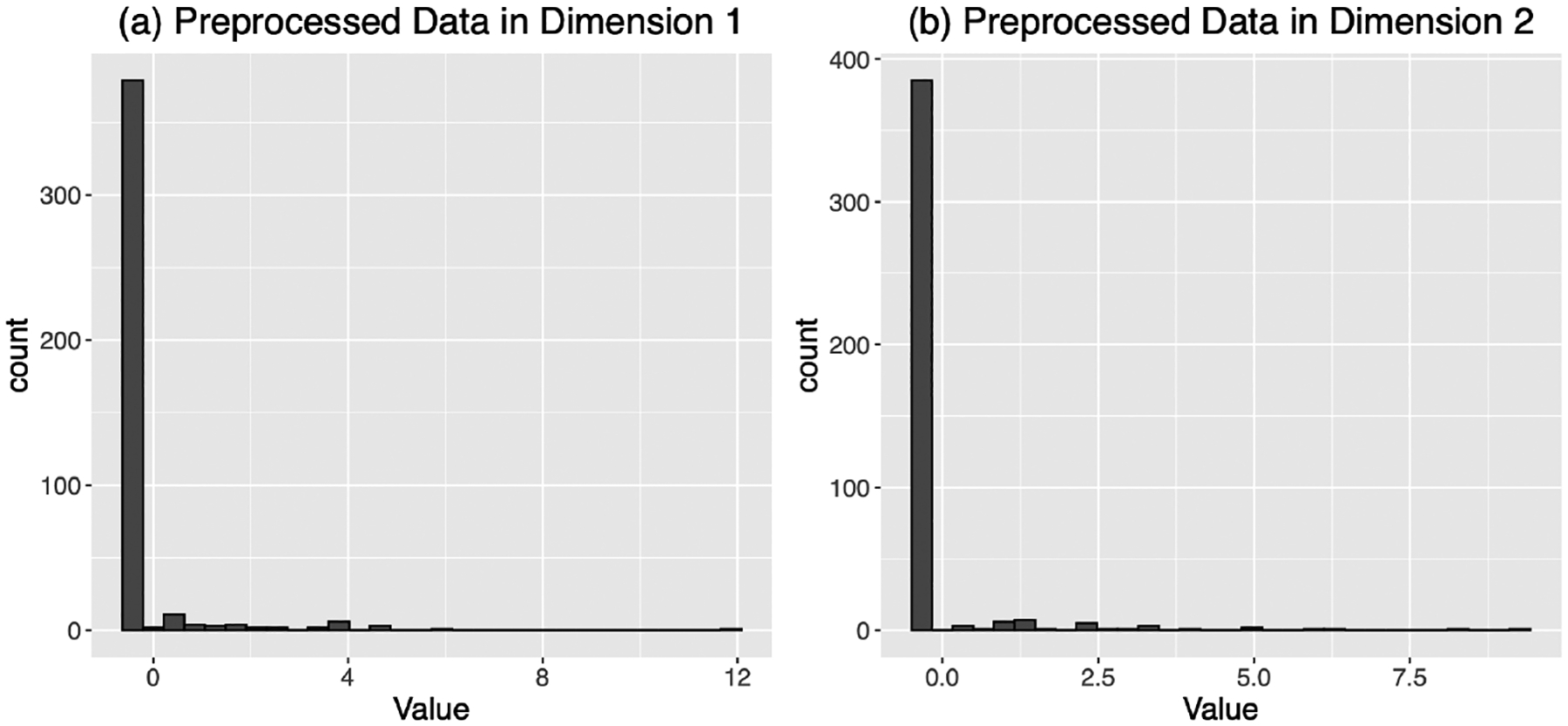
Histograms showing the distribution of normalized expression values for two representative dimensions. The highly skewed distributions demonstrate that most values are concentrated near zero, even after normalization.

Recent advances in high-throughput single-cell RNA sequencing (scRNA-Seq) technologies greatly enhance our understanding of cell-to-cell heterogeneity and cell lineages trajectories in development (Cao et al., 2019). One important goal of analyzing scRNA-Seq data is to cluster cells to identify cell subpopulations with different functions and gene expression patterns. The large number of detected genes in scRNA-Seq data makes the task of clustering cells a high-dimensional problem. In this section, we evaluate the proposed Bayesian sparse Gaussian mixture model using a benchmark scRNA-Seq data set (Darmanis et al., 2015), which is available at the data repository Gene Expression Omnibus (GSE67835, https://www.ncbi.nlm.nih.gov/geo/query/acc.cgi?acc=GSE67835). After excluding hybrid cells and filtering out lowly expressed genes (i.e., the total number of RNA-Seq counts over all non-hybrid cells is less than or equal to 10), we have p=18568 genes and n=420 cells in 8 cell types including fetal quiescent cells (110 cells), fetal replicating cells (25 cells), astrocytes cells (62 cells), neuron cells (131 cells), endothelial (20 cells), oligodendrocyte cells (38 cells), microglia cells (16 cells), and OPCs (16 cells). The original count data $y_{ji}$ for gene j in cell i is transformed into continuous type by taking base-2 logarithm and pseudo count 1, i.e., ${\text{log}}_{2}\left(y_{ji}+1\right)$. Then we divide each $y_{ji}$ by the total expression of each cell, i.e., ${\text{log}}_{2}\left(y_{ji}+1\right)/\sum_{j=1}^{p}{\text{log}}_{2}\left(y_{ji}+1\right)$. Lastly we normalize the data such that the standardized expression levels have zero mean and unit variance for each gene. Note that after normalization the data are no longer sparse and exhibit a skewed distribution; however, most values remain small in magnitude (see Figure 5 for two randomly selected dimensions). In our theoretical studies, the sparsity assumption applies only to the underlying mean vector—not to the raw or normalized data—since the framework explicitly accounts for noise in each dimension. This assumption is primarily introduced to guarantee the method’s theoretical properties, although the algorithm itself remains applicable regardless of the exact sparsity level.

We apply the proposed Bayesian method to the scRNA-Seq data with the same hyperparameters as in the simulation study. We run the MCMC sampler for 10000 iterations and discard the first 5000 iterations as burn-in. For comparison, we implement several alternatives, including K-means (KM), PCA-KM, MClust, SKM, K-means after non-negative matrix factorization (NMF-KM) (Zhu et al., 2017), and K-means after t-distributed stochastic neighbor embedding algorithm (tSNE-KM) (Linderman et al., 2019). For PCA-KM and NMF-KM, we first project the data onto the top 10-dimensional feature space, then apply the KM algorithm to cluster the cells. For the KM-based method, the optimal number of clusters is determined by the Silhouette method.

To assess the sparsity structure in this dataset, we first examine ξ, the indicator variable in our spike-and-slab prior that determines whether each gene is associated with the spike or slab component. Our analysis reveals that the posterior means of ξ are predominantly zero, with 18,321 out of 18,568 genes exhibiting a posterior mean of exactly zero. This indicates that a vast majority of genes are strongly associated with the spike component, reflecting a high level of sparsity in the underlying mean structure. Therefore, our model effectively captures the sparsity in the data, even after normalization.

To measure the performance of clustering results, we use not only the aforementioned ARI but another commonly-used criteria in the single-cell literature: normalized mutual information (NMI) (Ghosh and Acharya, 2011). Formally, with the same notations as in Section 4.2, the NMI is defined as

$$\text{NMI}\left({𝒞}^{*},{𝒞}^{†}\right)=\frac{\sum_{k=1}^{K^{*}}\sum_{k^{\prime }=1}^{K^{†}}\frac{n_{k,k^{\prime }}}{n}\text{log}\left(\frac{n_{k,k^{\prime }}}{n}\right)-\sum_{k=1}^{K^{*}}\frac{n_{k}^{*}}{n}-\sum_{k^{\prime }=1}^{K^{†}}\frac{n_{k^{\prime }}^{†}}{n}\text{log}\left(\frac{n_{k^{\prime }}^{†}}{n}\right)}{\sqrt{\sum_{k=1}^{K^{*}}\frac{n_{k}^{*}}{n}\text{log}\left(\frac{n_{k}^{*}}{n}\right)\sum_{k=1}^{K^{†}}\frac{n_{k^{\prime }}^{†}}{n}\text{log}\left(\frac{n_{k^{\prime }}^{†}}{n}\right)}}.$$

**Figure 6:**
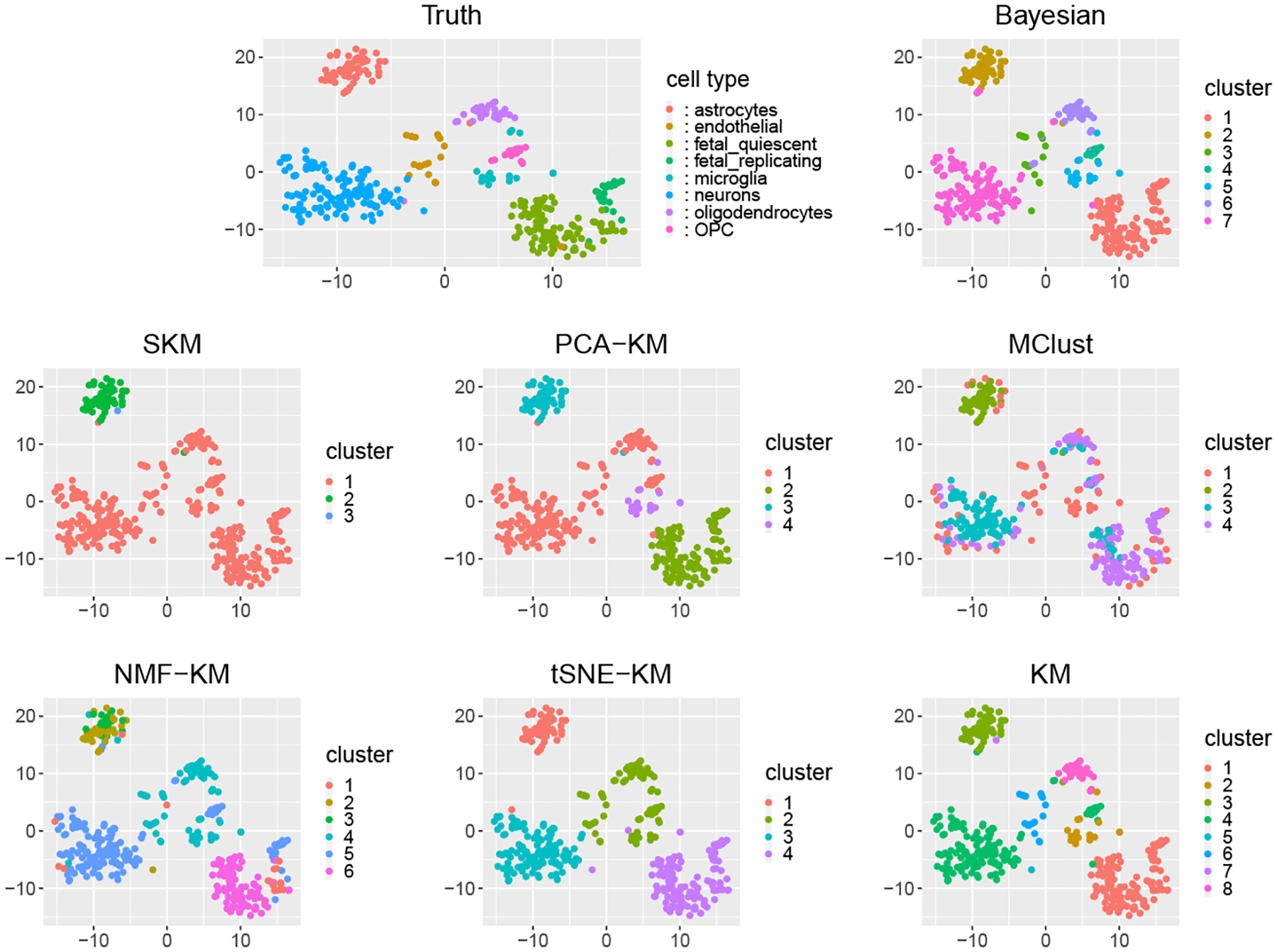
Clustering results of scRNA-Seq data corresponding to different methods. Data points are embedded into two-dimensional subspace by tSNE embedding.

Table 2 reports the ARIs and NMIs under the proposed method and alternatives, showing that the proposed model results in the highest ARI and NMI and achieves the best clustering accuracy. Figure 6 plots the true cell types and the estimated cluster memberships under all the methods. Although the proposed Bayesian method underestimates the number of cell types by 1 and yields K=7, it can identify most cell types except for fetal quiescent and fetal replicating cells. The KM method correctly estimates K=8 cell types. However, it cannot recognize OPC cells and gives two additional small clusters that are not interpretable. Other methods tend to underestimate K. In particular, SKM and MClust estimate K=3 and K=4 respectively, and perform worse than others in terms of much lower ARIs and NMIs, as shown in Table 2. Both PCA-KM and tSNE-KM estimate K=4 by correctly identifying the astrocytes cell type and merging fetal quiescent and fetal replicating cell types into one cluster. For the other five cell types, PCA-KM identifies microglia cell type and merges oligodendrocytes, OPC, endothelial, and neuron cell types into one cluster, while tSNE-KM identifies the neuron cell type and merges oligodendrocytes, OPC, microglia, and endothelial cells as one cluster. NMF-KM is able to identify neuron, fetal quiescent, and fetal replicating cell types but cannot distinguish others. tSNE-KM identifies oligodendrocytes, OPC, microglia, and endothelial cells as one cluster.

We further examine the alternative methods when the number of clusters is set to be the true number of cell types (K=8). Appendix Figure A4 plots the estimated clustering memberships under all the alternative methods. As shown in Figure A4, MClust cannot distinguish fetal quiescent and fetal replicating cell types and merges OPC and oligodendrocytes cell types into one cluster. PCA-KM and tSNE-KM return clusters with relatively similar sizes and hence their performance on small clusters are not satisfactory. SKM and NMF-KM perform significantly worse than others since they do not correctly identify any single cell type. Appendix Table A2 reports the resulting ARIs and NMIs, showing that the proposed Bayesian method still yields the highest ARI and NMI even though the number of clusters is correctly pre-specified for all alternative methods.

## 6. Discussion

We propose a Bayesian approach for high-dimensional Gaussian mixtures where the cluster mean vectors exhibit certain sparsity structure. We fully investigate the minimax risk for estimating the mean matrix, show that the posterior contraction rate is minimax optimal, and obtain an error bound for the mis-clustering error. Our approach demonstrates superior performance in both simulations and real-world applications.

There still exist challenges that need further research. One extension is to consider scenarios where the cluster-specific covariance matrices have some structures, such as sparse spiked structures (Xie et al., 2022). Exploring the theoretical properties of covariance matrix estimation could be an interesting future direction. On the implementation side, algorithms based on Markov chain Monte Carlo can be computationally expensive in ultra-high dimensions. Certain optimization-based alternatives, such as variational Bayes methods (Ray and Szabó, 2021) can be attractive. Developing the underlying backbone theory for variational Bayes approaches can be a promising future research direction as well.

## Supplementary Material

1

## Figures and Tables

**Table 1:** Average (standard deviation) adjusted Rand indices (ARIs) in Scenario I with different choice of the number of clusters $K^{*}$ and support size s.

	$K^{*}=3$		$K^{*}=5$	
	s=6	s=12	s=6	s=12
Bayesian	0.84 (0.19)	0.98 (0.01)	0.94 (0.03)	0.99 (0.01)
PCA-KM	0.54 (0.04)	0.55 (0.04)	0.64 (0.17)	0.60 (0.18)
MClust	0.54 (0.04)	0.55 (0.04)	0.81 (0.13)	0.78 (0.05)
SKM	0.55 (0.04)	0.55 (0.04)	0.54 (0.21)	0.74 (0.13)
CHIME	0.53 (0.10)	0.63 (0.18)	0.52 (0.27)	0.54 (0.29)

**Table 2:** Estimate of the number of clusters K, ARIs, and NMIs obtained by applying different methods to the scRNA-Seq data set.

Methods	Bayesian	SKM	PCA-KM	MClust	NMF-KM	tSNE-KM	KM
Estimate of K	7	3	4	4	6	4	9
ARI	0.84	0.15	0.59	0.33	0.63	0.78	0.79
NMI	0.80	0.22	0.58	0.35	0.61	0.70	0.77
