# Supplementary material for: Bayesian Sparse Gaussian Mixture Model for Clustering in High Dimensions
Source: J Mach Learn Res. Author manuscript; Available in PMC 2026 Mar 7. (PMC12965251)
Supplement: 1 [file NIHMS2074287-supplement-1.pdf]

Appendix A. Proofs for Section 2

Proof of Theorem 1 We define two subspaces of Θ_K^* as follows. We consider the case where $\boldsymbol{\Sigma}^* = \boldsymbol{I}_p$. Without loss of generality, we assume that n/K is an integer. If not, let $n' = \lfloor n/K \rfloor K$. Then, we derive a lower bound for a smaller parameter space $\Theta' \subset \mathbb{R}^{p\times K} \times \mathbb{R}^{n'\times K}$, which also serves as a lower bound for the original parameter space $\Theta_K^* \subset \mathbb{R}^{p\times K} \times \mathbb{R}^{n\times K}$.

We define the first subspace

$$\Theta_{K1}^{*}=\left\{(\boldsymbol{\mu},\boldsymbol{L},\boldsymbol{\Sigma})\in\Theta_K^{*}:\boldsymbol{\mu}=[\mu_0\boldsymbol{w}_1,\cdots,\mu_0\boldsymbol{w}_K],\mu_0=\sqrt{\frac{c_1\log K}{s}},\mathrm{supp}(\boldsymbol{\mu})=S,\boldsymbol{\Sigma}^{*}=\boldsymbol{I}_p\right\}$$

for some fixed sparsity support $S\subset[p]$ with $|S|=s$, a constant $c_1>0$, and $\boldsymbol{w}_1,\dots,\boldsymbol{w}_K\in\{0,1\}^p$. Specifically, we choose the vectors $\boldsymbol{w}_1,\dots,\boldsymbol{w}_K$ such that $\|(\boldsymbol{w}_i)_S\|_0=s$ for all $i\in[K]$ and $\|(\boldsymbol{w}_i)_S-(\boldsymbol{w}_j)_S\|_2^2>s/2$ for $i\neq j$. By Lemma 4.10 in Massart (2007), there exists $\{(\boldsymbol{w}_1)_S,\dots,(\boldsymbol{w}_K)_S\}\subset\{0,1\}^s$ satisfying these properties for $K\leq s$. Next we define the second subspace. Note that each $\boldsymbol{L}$ can be associated with a mapping $z:[n]\to[K]$ such that $\boldsymbol{l}_i=\boldsymbol{e}_{z_i}$ where $\boldsymbol{e}_i$ is the vector whose ith entry is 1 and 0 elsewhere. Then define

$$\Theta_{K2}^{*}=\left\{(\boldsymbol{\mu},\boldsymbol{L},\boldsymbol{\Sigma})\in\Theta_K^{*}:z^{-1}(k)=\left\{\frac{(k-1)n}{K}+1,\dots,\frac{kn}{K},\boldsymbol{\Sigma}^{*}=\boldsymbol{I}_p\right\},|\mathrm{supp}(\boldsymbol{\mu})|=s\right\},$$

where $\mathrm{supp}(\cdot)$ denotes the set of indices of the non-zero rows of a matrix.

■ We first consider the minimax lower bound over Θ_{K1}^{*}. By Lemma 4.10 in Massart (2007) we know there exist $\{\boldsymbol{w}_1,\cdots,\boldsymbol{w}_K\}\subset\{0,1\}^s$ such that $\|\boldsymbol{w}_i-\boldsymbol{w}_j\|^2>s/2$. Consider an ϵ_1-ball of Θ_{K1}^{*} with respect to the metric $d_1((\boldsymbol{\mu},\boldsymbol{L}_1),(\boldsymbol{\mu},\boldsymbol{L}_2))=\|\boldsymbol{\mu}\boldsymbol{L}_1^T-\boldsymbol{\mu}\boldsymbol{L}_2^T\|_F/\sqrt{n}$. Suppose $\boldsymbol{L}_1$ and $\boldsymbol{L}_2$ are associated with mappings $z_1,z_2:[n]\mapsto[K]$. We have

$$4n\epsilon_1^2>\|\boldsymbol{\mu}\boldsymbol{L}_1^T-\boldsymbol{\mu}\boldsymbol{L}_2^T\|_F^2\geq\mu_0^2\sum_{i=1}^{n}\|\boldsymbol{w}_{z_1(i)}-\boldsymbol{w}_{z_2(i)}\|_F^2\geq\mu_0^2|\{i:z_1(i)\neq z_2(i)\}|\frac{s}{2}.$$

Let $\epsilon_1^2=(c_1\log K)/48$. Since $\mu_0^2=(c_1\log K)/s$, we have $|\{i:z_1(i)\neq z_2(i)\}|\leq n/6$. Denote $B_\epsilon(\boldsymbol{\mu}\boldsymbol{L}^T):=\{\boldsymbol{L}_1\in\mathcal{L}_K:d_1((\boldsymbol{\mu},\boldsymbol{L}),(\boldsymbol{\mu},\boldsymbol{L}_1))\leq\epsilon\}$ for any $\boldsymbol{\mu}\in\mathbb{R}^{p\times K}$ and $\boldsymbol{L}\in\mathcal{L}_K$. Then for any $(\boldsymbol{\mu},\boldsymbol{L})\in\Theta_{K1}^{*}$, we have

$$\begin{aligned}|B_{\epsilon_1}(\boldsymbol{\mu}\boldsymbol{L}^T)|&\leq\binom{n}{n/6}K^{n/6}\leq\exp\left(n\log 6-\frac{5n}{6}\log 5+\frac{n}{6}\log K\right)\\&=\exp\left(n\log 6-\frac{5}{6}\frac{\log 5}{\log 6}n\log 6+\frac{n}{6}\log K\right)\leq\exp\left(\frac{3}{10}n\log 6+\frac{n}{6}\log K\right)\\&\leq\exp\left(\frac{3}{10}\frac{\log 6}{\log 2}n\log K+\frac{1}{6}n\log K\right)\leq\exp\left(0.95n\log K\right),\end{aligned}$$

where the second inequality comes from the Stirling's formula and the fourth inequality is due to the fact that $K\geq 2$. Denote $M(\epsilon_1,\Theta_{K1}^{*},d_1)$ as the ϵ-packing number of Θ_{K1}^{*} with respect to the metric d_1. Since $|\Theta_{K1}^{*}|=K^n$, we have

$$\log M\left(\epsilon_1,\Theta_{K1}^{*},d_1\right)\geq\log\frac{K^n}{\binom{n}{n/6}K^{n/6}}\geq\frac{1}{20}n\log K.$$

Note that $D_{KL}(\mathbb{P}_{\boldsymbol{\mu}\boldsymbol{L}_1^T}\|\mathbb{P}_{\boldsymbol{\mu}\boldsymbol{L}_2^T})=\frac{1}{2}\|\boldsymbol{\mu}\boldsymbol{L}_1-\boldsymbol{\mu}\boldsymbol{L}_2^T\|_F^2\leq\frac{2\mu_0^2sn}{2}=c_1n\log K$. Therefore, by the generalized Fano's lemma (Yu, 1997)

$$\inf_{\hat{\boldsymbol{\mu}},\hat{\boldsymbol{L}}}\sup_{(\boldsymbol{\mu}^*,\boldsymbol{L}^*)\in\Theta_{K1}^{*}}\mathbb{P}_*\left(\|\hat{\boldsymbol{\mu}}\hat{\boldsymbol{L}}^T-(\boldsymbol{\mu}^*)(\boldsymbol{L}^*)^T\|_F^2\geq c_2n\log K\right)\geq 1-\frac{c_1n\log K+\log 2}{n\log K/20}\geq 0.9$$

with some constant $c_2 = c_1/48 > 0$ for sufficiently small $c_1 < 1/20$ and sufficiently large n where $\mathbb{P}_*$ represents the probability measure under $(\boldsymbol{\mu}^*, \boldsymbol{L}^*, \boldsymbol{\Sigma}^*)$. By Markov's inequality,

$$\begin{aligned}&\inf_{\hat{\boldsymbol{\mu}},\hat{\boldsymbol{L}}} \sup_{(\boldsymbol{\mu}^*,\boldsymbol{L}^*)\in\Theta^*_{K1}} \mathbb{E}_* \left(\|\hat{\boldsymbol{\mu}}\hat{\boldsymbol{L}}^T - (\boldsymbol{\mu}^*)(\boldsymbol{L}^*)^T\|_F^2 \right) \\ &\quad\geq \inf_{\hat{\boldsymbol{\mu}},\hat{\boldsymbol{L}}} \sup_{(\boldsymbol{\mu}^*,\boldsymbol{L}^*)\in\Theta^*_{K1}} c_2 n \log K \, \mathbb{P}_* \left(\|\hat{\boldsymbol{\mu}}\hat{\boldsymbol{L}}^T - (\boldsymbol{\mu}^*)(\boldsymbol{L}^*)^T\|_F^2 \geq c_2 n \log K \right) \geq 0.9 c_2 n \log K.\end{aligned}$$

■ We next consider the minimax lower bound over Θ^*_{K2}. By the construction of Θ^*_{K2}, we have $\boldsymbol{\mu}\boldsymbol{L}^T = \begin{pmatrix}\boldsymbol{\mu}_1 & \dots & \boldsymbol{\mu}_1 & \boldsymbol{\mu}_2 & \dots & \boldsymbol{\mu}_2 & \dots & \boldsymbol{\mu}_K & \dots & \boldsymbol{\mu}_K\end{pmatrix}$. Thus, $\|\boldsymbol{\mu}\boldsymbol{L}^T - \boldsymbol{\mu}'\boldsymbol{L}^T\|_F^2 = (n/K)\sum_{k=1}^K \|\boldsymbol{\mu}_k - \boldsymbol{\mu}'_k\|_2^2$. Let $\boldsymbol{\mu}_k = \begin{pmatrix}\lambda_k & a_0\boldsymbol{v}^T\end{pmatrix}^T$ for $k \in [K]$, where λ_k's are distinct scalars for $k \in [K]$, $\boldsymbol{v} \in \{0,1\}^{p-1}$, and $a_0 > 0$ is to be specified later. By Lemma 4.10 in Massart (2007), there exists $\{\boldsymbol{v}^{(1)}, \cdots, \boldsymbol{v}^{(N)}\} \subset \{0,1\}^{p-1}$ such that

- $\|\boldsymbol{v}^{(i)} - \boldsymbol{v}^{(j)}\|^2 > \frac{s-1}{2}$ for $i \neq j$,
- $\|\boldsymbol{v}^{(i)}\|_0 = s-1$ for all $i \in [N]$,
- $N > \exp(c_4(s-1)\log\frac{p-1}{s-1})$ for some $c_4 \geq 0.233$.

For each distinct pair $\boldsymbol{\mu}, \boldsymbol{\mu}'$, we choose

$$\boldsymbol{\mu} = \begin{pmatrix}\lambda_1 & \dots & \lambda_K \\ a_0\boldsymbol{v} & \dots & a_0\boldsymbol{v}\end{pmatrix} \quad \text{and} \quad \boldsymbol{\mu}' = \begin{pmatrix}\lambda_1 & \dots & \lambda_K \\ a_0\boldsymbol{v}' & \dots & a_0\boldsymbol{v}'\end{pmatrix}$$

such that $\boldsymbol{v}, \boldsymbol{v}' \in \{\boldsymbol{v}^{(1)}, \dots, \boldsymbol{v}^{(N)}\}$, $\boldsymbol{v} \neq \boldsymbol{v}'$. Then we consider an ϵ_2-ball in Θ^*_{K2} with respect to the metric $d_1((\boldsymbol{\mu}, \boldsymbol{L}_1), (\boldsymbol{\mu}, \boldsymbol{L}_2)) = \|\boldsymbol{\mu}\boldsymbol{L}_1^T - \boldsymbol{\mu}\boldsymbol{L}_2^T\|/\sqrt{n}$. We have

$$\frac{1}{n}\|\boldsymbol{\mu}\boldsymbol{L}^T - \boldsymbol{\mu}'\boldsymbol{L}^T\|_F^2 = \frac{1}{K}\sum_{k=1}^K \|\boldsymbol{\mu}_k - \boldsymbol{\mu}'_k\|_2^2 = \frac{1}{K}\sum_{k=1}^K a_0^2\|\boldsymbol{v} - \boldsymbol{v}'\|_2^2 > \frac{1}{K}a_0^2\frac{(s-1)K}{2} = \frac{a_0^2(s-1)}{2}.$$

Let $a_0^2 = 2\{sc_3\log(p/s)\}/\{n(s-1)\}$ and $\epsilon_2^2 = \{c_3 s\log(p/s)\}/n$, where $c_3 > 0$ is a constant to be specified later. Then we have

$$M(\epsilon_2, \Theta^*_{K2}, d_1) \geq \exp\left(c_4(s-1)\log\frac{p-1}{s-1}\right) \geq \exp\left(\frac{c_4}{4}s\log\frac{p}{s}\right)$$

for $s \geq 2$ because $(p-1)/(s-1) \geq \sqrt{p/s}$. Note that for any $(\boldsymbol{\mu}, \boldsymbol{L}), (\boldsymbol{\mu}', \boldsymbol{L}) \in \Theta^*_{K2}$,

$$D_{KL}(\mathbb{P}_{\boldsymbol{\mu}\boldsymbol{L}^T}\|\mathbb{P}_{\boldsymbol{\mu}'\boldsymbol{L}^T}) = \frac{1}{2}\|\boldsymbol{\mu}\boldsymbol{L} - \boldsymbol{\mu}'\boldsymbol{L}^T\|_F^2 \leq a_0^2 sn.$$

Without loss of generality we assume $s \geq 11$. Then $\log 2 \leq \frac{10}{11}\frac{c_4}{4}s\log\left(\frac{p}{s}\right)$ since $c_4 \geq 0.2$, and

$$\frac{d_{KL}(\Theta^*_{K2}) + \log 2}{\log M(\epsilon_2, \Theta^*_{K2}, d_1)} \leq \frac{a_0^2 sn + \log 2}{\frac{c_4}{4}s\log\frac{p}{s}} \leq \frac{16c_3}{c_4} + \frac{10}{11}.$$

Therefore, by selecting $c_3 = c_4/(22 \times 16)$, we have

$$\inf_{\hat{\boldsymbol{\mu}},\hat{\boldsymbol{L}}} \sup_{(\boldsymbol{\mu}^*,\boldsymbol{L}^*)\in\Theta^*_{K2}} \mathbb{P}_* \left(\|\hat{\boldsymbol{\mu}}\hat{\boldsymbol{L}}^T - (\boldsymbol{\mu}^*)(\boldsymbol{L}^*)^T\|_F^2 \geq c_3 s \log\frac{p}{s} \right) \geq \frac{1}{22}$$

for $c_4 \geq 0.233$, and for sufficiently large n. It follows from Markov's inequality that

$$\inf_{\hat{\boldsymbol{\mu}},\hat{\boldsymbol{L}}} \sup_{(\boldsymbol{\mu}^*,\boldsymbol{L}^*)\in\Theta^*_{K2}} \mathbb{E}_* \left(\|\hat{\boldsymbol{\mu}}\hat{\boldsymbol{L}}^T - (\boldsymbol{\mu}^*)(\boldsymbol{L}^*)^T\|_F^2 \right) \geq \frac{c_3}{22} s \log\frac{p}{s}.$$

■ Now we combined the minimax lower bounds over Θ^*_{K1} and Θ^*_{K2}:

$$\begin{aligned}
&\inf_{\hat{\boldsymbol{\mu}},\hat{\boldsymbol{L}}} \sup_{(\boldsymbol{\mu}^*,\boldsymbol{L}^*)\in\Theta^*_{K}} \mathbb{E}_* \left(\|\hat{\boldsymbol{\mu}}\hat{\boldsymbol{L}}^T - (\boldsymbol{\mu}^*)(\boldsymbol{L}^*)^T\|_F^2 \right) \\
&\quad \geq \inf_{\hat{\boldsymbol{\mu}},\hat{\boldsymbol{L}}} \max_{j\in\{1,2,\}} \sup_{(\boldsymbol{\mu}^*,\boldsymbol{L}^*)\in\Theta^*_{Kj}} \mathbb{E}_* \left(\|\hat{\boldsymbol{\mu}}\hat{\boldsymbol{L}}^T - (\boldsymbol{\mu}^*)(\boldsymbol{L}^*)^T\|_F^2 \right) \\
&\quad \geq \max\left\{0.9 c_2 n \log K, \frac{c_3}{22} s \log\frac{p}{s}\right\} \geq C\left(s\log\frac{p}{s} + n\log K\right).
\end{aligned}$$

■

Proof of Theorem 3 Note that by a basic inequality, we have

$$\begin{aligned}
&\|\boldsymbol{Y} - (\boldsymbol{\mu}^*)(\boldsymbol{L}^*)^T\|_F^2 \geq \|\boldsymbol{Y} - \widehat{\boldsymbol{\mu}}\widehat{\boldsymbol{L}}^T\|_F^2 = \|(\boldsymbol{Y} - (\boldsymbol{\mu}^*)(\boldsymbol{L}^*)^T) + ((\boldsymbol{\mu}^*)(\boldsymbol{L}^*)^T - \widehat{\boldsymbol{\mu}}\widehat{\boldsymbol{L}}^T)\|_F^2 \\
&= \|\boldsymbol{Y} - (\boldsymbol{\mu}^*)(\boldsymbol{L}^*)^T\|_F^2 + \|(\boldsymbol{\mu}^*)(\boldsymbol{L}^*)^T - \widehat{\boldsymbol{\mu}}\widehat{\boldsymbol{L}}^T\|_F^2 + 2\langle \boldsymbol{Y} - (\boldsymbol{\mu}^*)(\boldsymbol{L}^*)^T, (\boldsymbol{\mu}^*)(\boldsymbol{L}^*)^T - \widehat{\boldsymbol{\mu}}\widehat{\boldsymbol{L}}^T\rangle_F \\
&= \|\boldsymbol{Y} - (\boldsymbol{\mu}^*)(\boldsymbol{L}^*)^T\|_F^2 + \|(\boldsymbol{\mu}^*)(\boldsymbol{L}^*)^T - \widehat{\boldsymbol{\mu}}\widehat{\boldsymbol{L}}^T\|_F^2 \\
&\quad + 2\left\langle \boldsymbol{Y} - (\boldsymbol{\mu}^*)(\boldsymbol{L}^*)^T, \frac{(\boldsymbol{\mu}^*)(\boldsymbol{L}^*)^T - \widehat{\boldsymbol{\mu}}\widehat{\boldsymbol{L}}^T}{\|(\boldsymbol{\mu}^*)(\boldsymbol{L}^*)^T - \widehat{\boldsymbol{\mu}}\widehat{\boldsymbol{L}}^T\|_F} \right\rangle_F \times \|(\boldsymbol{\mu}^*)(\boldsymbol{L}^*)^T - \widehat{\boldsymbol{\mu}}\widehat{\boldsymbol{L}}^T\|_F,
\end{aligned}$$

where $\langle\cdot,\cdot\rangle_F$ is the Frobenius inner product defined by $\langle \boldsymbol{A}, \boldsymbol{B}\rangle_F = \mathrm{tr}(\boldsymbol{A}^T\boldsymbol{B})$. After rearranging the terms on the both sides of the inequality, we obtain

$$\mathbb{E}_* \|(\boldsymbol{\mu}^*)(\boldsymbol{L}^*)^T - \widehat{\boldsymbol{\mu}}\widehat{\boldsymbol{L}}^T\|_F \leq 2\,\mathbb{E}_* \left[\sup_{(\boldsymbol{\mu},\boldsymbol{L})\in\Theta_K} \left\langle \boldsymbol{E}, (\boldsymbol{\Sigma}^*)^{\frac{1}{2}} \frac{\boldsymbol{\mu}\boldsymbol{L}^T - (\boldsymbol{\mu}^*)(\boldsymbol{L}^*)^T}{\|\boldsymbol{\mu}\boldsymbol{L}^T - (\boldsymbol{\mu}^*)(\boldsymbol{L}^*)^T\|_F} \right\rangle_F \right],$$

where $\boldsymbol{E} = (\boldsymbol{\Sigma}^*)^{-\frac{1}{2}}(\boldsymbol{Y} - (\boldsymbol{\mu}^*)(\boldsymbol{L}^*)^T)$ is the standardized noise matrix.

Consider the set of matrices

$$\widetilde{\Theta}_K = \left\{ (\boldsymbol{\Sigma}^*)^{\frac{1}{2}} \frac{\boldsymbol{\mu}\boldsymbol{L}^T - (\boldsymbol{\mu}^*)(\boldsymbol{L}^*)^T}{\|\boldsymbol{\mu}\boldsymbol{L}^T - (\boldsymbol{\mu}^*)(\boldsymbol{L}^*)^T\|_F} : \boldsymbol{\mu} \in \mathbb{R}^{p\times K}, |\mathrm{supp}(\boldsymbol{\mu})| \leq s, \boldsymbol{L} \in \mathcal{L}_K \right\}.$$

To obtain an upper bound of the right hand side of the inequality above, we use some tools of maximal inequality of empirical process. Specifically, we define a stochastic process $X(\boldsymbol{B}) = \langle \boldsymbol{E}, \boldsymbol{B}\rangle_F$ indexed by a $p \times n$ matrix $\boldsymbol{B}$. Since the entries of $\boldsymbol{E}$ are i.i.d. standard Gaussian, it follows that $X(\boldsymbol{B})$ is sub-Gaussian. Then by Corollary 8.5 in Kosorok (2008),

$$\mathbb{E}_* \left[\sup_{(\boldsymbol{\mu},\boldsymbol{L})\in\Theta_K} \left\langle \boldsymbol{E}, (\boldsymbol{\Sigma}^*)^{\frac{1}{2}} \frac{\boldsymbol{\mu}\boldsymbol{L}^T - (\boldsymbol{\mu}^*)(\boldsymbol{L}^*)^T}{\|\boldsymbol{\mu}\boldsymbol{L}^T - (\boldsymbol{\mu}^*)(\boldsymbol{L}^*)^T\|_F} \right\rangle_F \right] \lesssim \int_0^{\mathrm{diam}(\widetilde{\Theta}_K)} \sqrt{\log N(\epsilon, \widetilde{\Theta}_K, \|\cdot\|_F)}\mathrm{d}\epsilon.$$

Obtaining a sharp upper bound of $N(\epsilon, \widetilde{\Theta}_K, \|\cdot\|_F)$ is quite involved. We breakdown the computation of a sharp bound for the covering number of $\widetilde{\Theta}_K$ as follows.

Step 1: Decompose $\widetilde{\Theta}_K$ into unions of subspaces $\mathcal{E}_j^K$ where $\|\boldsymbol{\mu}\boldsymbol{L}^T - (\boldsymbol{\mu}^*)(\boldsymbol{L}^*)^T\|_F$ is bounded. Define a function $B : \mathbb{R}^{p\times K} \times \mathcal{L}_K \mapsto \widetilde{\Theta}_K$ as $B(\boldsymbol{\mu}, \boldsymbol{L}) = (\boldsymbol{\Sigma}^*)^{\frac{1}{2}} \frac{\boldsymbol{\mu}\boldsymbol{L}^T - (\boldsymbol{\mu}^*)(\boldsymbol{L}^*)^T}{\|\boldsymbol{\mu}\boldsymbol{L}^T - (\boldsymbol{\mu}^*)(\boldsymbol{L}^*)^T\|_F}$. Then we have

$$
\begin{aligned}
\|B(\boldsymbol{\mu}, \boldsymbol{L}) - B(\boldsymbol{\mu}', \boldsymbol{L}')\|_F &\leq \left\|(\boldsymbol{\Sigma}^*)^{\frac{1}{2}}\right\|_2 \left\| \frac{\boldsymbol{\mu}\boldsymbol{L}^T - (\boldsymbol{\mu}^*)(\boldsymbol{L}^*)^T}{\|\boldsymbol{\mu}\boldsymbol{L}^T - (\boldsymbol{\mu}^*)(\boldsymbol{L}^*)^T\|_F} - \frac{(\boldsymbol{\mu}')(\boldsymbol{L}')^T - (\boldsymbol{\mu}^*)(\boldsymbol{L}^*)^T}{\|(\boldsymbol{\mu}')(\boldsymbol{L}')^T - (\boldsymbol{\mu}^*)(\boldsymbol{L}^*)^T\|_F} \right\|_F \\
&= \lambda_{\max}(\boldsymbol{\Sigma}^*)^{\frac{1}{2}} \Bigg\| \frac{\boldsymbol{\mu}\boldsymbol{L}^T \|(\boldsymbol{\mu}')(\boldsymbol{L}')^T - (\boldsymbol{\mu}^*)(\boldsymbol{L}^*)^T\|_F - (\boldsymbol{\mu}')(\boldsymbol{L}')^T \|(\boldsymbol{\mu}^*)(\boldsymbol{L}^*)^T - \boldsymbol{\mu}\boldsymbol{L}^T\|_F}{\|\boldsymbol{\mu}\boldsymbol{L}^T - (\boldsymbol{\mu}^*)(\boldsymbol{L}^*)^T\|_F \|(\boldsymbol{\mu}')(\boldsymbol{L}')^T - (\boldsymbol{\mu}^*)(\boldsymbol{L}^*)^T\|_F} \\
&\quad - \frac{(\boldsymbol{\mu}^*)(\boldsymbol{L}^*)^T (\|(\boldsymbol{\mu}')(\boldsymbol{L}')^T - (\boldsymbol{\mu}^*)(\boldsymbol{L}^*)^T\|_F - \|\boldsymbol{\mu}\boldsymbol{L}^T - (\boldsymbol{\mu}^*)(\boldsymbol{L}^*)^T\|_F)}{\|\boldsymbol{\mu}\boldsymbol{L}^T - (\boldsymbol{\mu}^*)(\boldsymbol{L}^*)^T\|_F \|(\boldsymbol{\mu}')(\boldsymbol{L}')^T - (\boldsymbol{\mu}^*)(\boldsymbol{L}^*)^T\|_F} \Bigg\|_F \\
&\leq \lambda_{\max}(\boldsymbol{\Sigma}^*)^{\frac{1}{2}} \Bigg(\frac{\|\boldsymbol{\mu}\boldsymbol{L}^T - (\boldsymbol{\mu}')(\boldsymbol{L}')^T\|_F}{\|\boldsymbol{\mu}\boldsymbol{L}^T - (\boldsymbol{\mu}^*)(\boldsymbol{L}^*)^T\|_F} \\
&\quad + \frac{\big| \|(\boldsymbol{\mu}')(\boldsymbol{L}')^T - (\boldsymbol{\mu}^*)(\boldsymbol{L}^*)^T\|_F - \|\boldsymbol{\mu}\boldsymbol{L}^T - (\boldsymbol{\mu}^*)(\boldsymbol{L}^*)^T\|_F \big|}{\|\boldsymbol{\mu}\boldsymbol{L}^T - (\boldsymbol{\mu}^*)(\boldsymbol{L}^*)^T\|_F} \Bigg) \\
&\leq 2\lambda_{\max}(\boldsymbol{\Sigma}^*)^{\frac{1}{2}} \frac{\|\boldsymbol{\mu}\boldsymbol{L}^T - (\boldsymbol{\mu}')(\boldsymbol{L}')^T\|_F}{\|\boldsymbol{\mu}\boldsymbol{L}^T - (\boldsymbol{\mu}^*)(\boldsymbol{L}^*)^T\|_F}.
\end{aligned}
$$

We split Θ_K as follows. Define

$$
\mathcal{E}_j^K = \left\{ (\boldsymbol{\mu}, \boldsymbol{L}) : \boldsymbol{\mu} \in \mathbb{R}^{p\times K}, |\mathrm{supp}(\boldsymbol{\mu})| \leq s, \boldsymbol{L} \in \mathcal{L}_K, \|\boldsymbol{\mu}\boldsymbol{L}^T - (\boldsymbol{\mu}^*)(\boldsymbol{L}^*)^T\|_F \in (a_j, a_{j+1}] \right\},
$$

where the sequence $\{a_j\}_{j=-\infty}^{\infty}$ satisfies $\lim_{j\to\infty} a_j = \infty$ and $\lim_{j\to-\infty} a_j = 0$. We also require that $a_j \leq a_{j+1}/\sqrt{n}$. Suppose $\mathcal{N}_j^K$ is an $a_j\epsilon/2$-covering of $\mathcal{E}_j^K$ with respect to the Frobenius norm. Then, it follows that $\bigcup_{j=-\infty}^{\infty} B(\mathcal{N}_j^K)$ is an $\epsilon\lambda_{\max}(\boldsymbol{\Sigma}^*)^{\frac{1}{2}}$-covering of $\widetilde{\Theta}_K$ with respect to the Frobenius norm. Thus,

$$
N(\epsilon\lambda_{\max}(\boldsymbol{\Sigma}^*)^{\frac{1}{2}}, \widetilde{\Theta}_K, \|\cdot\|_F) \leq \sum_{j=-\infty}^{\infty} N(a_j\epsilon/2, \mathcal{E}_j^K, \|\cdot\|_F).
$$

Step 2: Show that $\boldsymbol{L}$ equals $\boldsymbol{L}^*$ up to a permutation for $(\boldsymbol{\mu}, \boldsymbol{L}) \in \mathcal{E}_j^K$ when a_{j+1} is small. Denote Δ as the minimum distance among all cluster centers, that is, $\Delta = \min_{i,j\in[K]} \|\boldsymbol{\mu}_i^* - \boldsymbol{\mu}_j^*\|_2$. For a fixed $\boldsymbol{L}$ which is induced by $z : [n] \mapsto [K]$, we denote $n_{gh} \triangleq |z^{-1}(g) \bigcap (z^*)^{-1}(h)|$, $n_g = \sum_{h=1}^K n_{gh}$, and $n_h^* = \sum_{g=1}^K n_{gh}$. We then have

$$
\begin{aligned}
\|\boldsymbol{\mu}\boldsymbol{L}^T - (\boldsymbol{\mu}^*)(\boldsymbol{L}^*)^T\|_F^2 &= \sum_{g=1}^K \sum_{h=1}^K n_{gh} \|\boldsymbol{\mu}_g - \boldsymbol{\mu}_h^*\|_2^2 \\
&= \sum_{g=1}^K \left\{ n_g \left\| \boldsymbol{\mu}_g - \frac{\sum_{h=1}^K n_{gh}\boldsymbol{\mu}_h^*}{n_g} \right\|_2^2 + \sum_{h=1}^K n_{gh} \|\boldsymbol{\mu}_h^*\|_2^2 - \frac{\left\|\sum_{h=1}^K n_{gh}\boldsymbol{\mu}_h^*\right\|_2^2}{n_g} \right\} \\
&= \sum_{g=1}^K n_g \left\| \boldsymbol{\mu}_g - \frac{\sum_{h=1}^K n_{gh}\boldsymbol{\mu}_h^*}{n_g} \right\|_2^2 + C_{\boldsymbol{L}},
\end{aligned}
$$

where $C_{\boldsymbol{L}} \triangleq \sum_{g=1}^{K}\left\{\sum_{h=1}^{K} n_{gh}\|\boldsymbol{\mu}_h^*\|_2^2 - \left\|\sum_{h=1}^{K} n_{gh}\boldsymbol{\mu}_h^*\right\|_2^2/n_g\right\}$.

Note that by Cauchy-Schwarz inequality and triangle inequality, we have

$$\left\|\sum_{h=1}^{K} n_{gh}\boldsymbol{\mu}_h^*\right\|_2^2 \le \left(\sum_{h=1}^{K} n_{gh}\left\|\boldsymbol{\mu}_h^*\right\|_2\right)^2 \le \sum_{h=1}^{K} n_{gh}\sum_{h=1}^{K} n_{gh}\|\boldsymbol{\mu}_h^*\|_2^2 = n_g\sum_{h=1}^{K} n_{gh}\|\boldsymbol{\mu}_h^*\|_2^2$$

for every $g \in [K]$, which implies $C_{\boldsymbol{L}} \ge 0$ for any $\boldsymbol{L}$. Note that if $\boldsymbol{LP} = \boldsymbol{L}^*$ for some permutation matrix $\boldsymbol{P} \in \mathcal{S}^{K\times K}$ where $\mathcal{S}^{K\times K}$ is the set of all $K \times K$ permutation matrices and τ which is the permutation function induced by $\boldsymbol{P}$, then for every $g \in [K]$, we have $n_g = n_{g,\tau(g)}$ and $n_{gh} = 0$ for $h \ne \tau(g)$. Thus $\left\|\sum_{h=1}^{K} n_{gh}\boldsymbol{\mu}_h^*\right\|_2^2 = n_{g,\tau(g)}^2\|\boldsymbol{\mu}_{\tau(g)}^*\|_2^2 = n_g n_{g,\tau(g)}\|\boldsymbol{\mu}_{\tau(g)}^*\|_2^2 = n_g\sum_{h=1}^{K} n_{gh}\|\boldsymbol{\mu}_h^*\|_2^2$, which implies $C_{\boldsymbol{L}} = 0$. Next, by rearranging the terms in the expression of $C_{\boldsymbol{L}}$ we have

$$\begin{aligned}\sum_{h=1}^{K} n_g n_{gh}\|\boldsymbol{\mu}_h^*\|_2^2 - \left\|\sum_{h=1}^{K} n_{gh}\boldsymbol{\mu}_h^*\right\|_2^2 &= \sum_{h=1}^{K} n_{gh}(n_g - n_{gh})\|\boldsymbol{\mu}_h^*\|_2^2 - \sum_{h_1=1}^{K}\sum_{h_2\ne h_1} n_{gh_1}n_{gh_2}(\boldsymbol{\mu}_{h_1}^*)^T\boldsymbol{\mu}_{h_2}^* \\ &= \sum_{h_1=1}^{K}\sum_{h_2\ne h_1}\left\{n_{gh_1}n_{gh_2}\|\boldsymbol{\mu}_{h_1}^*\|_2^2 - n_{gh_1}n_{gh_2}(\boldsymbol{\mu}_{h_1}^*)^T\boldsymbol{\mu}_{h_2}^*\right\} = \sum_{h_1=1}^{K}\sum_{h_2\ne h_1}\frac{1}{2}n_{gh_1}n_{gh_2}\|\boldsymbol{\mu}_{h_1}^* - \boldsymbol{\mu}_{h_2}^*\|_2^2.\end{aligned}$$

Suppose there is no permutation matrix $\boldsymbol{P} \in \mathcal{S}^{K\times K}$ such that $\boldsymbol{LP} = \boldsymbol{L}^*$. Then there exists some $g' \in [K]$ such that for some $h_1', h_2' \in [K]$ and $h_1' \ne h_2'$, $n_{g'h_1'} \ge 1$ and $n_{g'h_2'} \ge 1$. Furthermore, for such $g' \in [K]$ and h_1', h_2', $n_{g'h_1'}n_{g'h_2'} \ge (n_{g'} - 1)/(K-1)$. If this is not true, then we obtain $n_{g'h_1'}(n_{g'} - n_{g'h_1'}) = \sum_{h_2\ne h_1'} n_{g'h_1'}n_{g'h_2} < n_g - 1$ for the aforementioned h_1', and this contradicts to the fact that $n_{g'h_1'}(n_{g'} - n_{g'h_1'}) \ge n_{g'} - 1$ for $1 \le n_{g'h_1'} \le n_{g'} - 1$. The reason is that $n_{g'h_1'}(n_{g'} - n_{g'h_1'})$ is a quadratic function of $n_{g'h_1'}$ and the minimum is achieved when $n_{g'h_1'} = 1$ or $n_{g'h_1'} = n_{g'} - 1$. Therefore, we know that if $\boldsymbol{L}$ is not identical to $\boldsymbol{L}^*$ up to permutation, then there exists some $g' \in [K]$ and $h_1' \ne h_2'$ such that

$$\begin{aligned}C_{\boldsymbol{L}} &= \sum_{g=1}^{K}\left(\sum_{h=1}^{K} n_{gh}\|\boldsymbol{\mu}_h^*\|_2^2 - \frac{\left\|\sum_{h=1}^{K} n_{gh}\boldsymbol{\mu}_h^*\right\|_2^2}{n_g}\right) = \sum_{g=1}^{K}\sum_{h_1=1}^{K}\sum_{h_2\ne h_1}\frac{n_{gh_1}n_{gh_2}\|\boldsymbol{\mu}_{h_1}^* - \boldsymbol{\mu}_{h_2}^*\|_2^2}{2n_g} \\ &\ge \frac{n_{g'h_1'}n_{g'h_2'}\|\boldsymbol{\mu}_{h_1'}^* - \boldsymbol{\mu}_{h_2'}^*\|_2^2}{n_{g'}} \ge \frac{n_{g'} - 1}{n_{g'}(K-1)}\Delta^2 \ge \frac{\Delta^2}{2(K-1)}.\end{aligned}$$

Thus for $\mathcal{E}_j^K$, we can see that if $a_{j+1} \le \Delta/\sqrt{2(K-1)}$, then every $(\boldsymbol{\mu}, \boldsymbol{L}) \in \mathcal{E}_j^K$ satisfies $\boldsymbol{LP} = \boldsymbol{L}^*$ for some permutation matrix $\boldsymbol{P} \in \mathcal{S}^{K\times K}$.

Step 3: Reduction of covering numbers of $\mathcal{E}_j^K$'s for small a_{j+1}. For $(\boldsymbol{\mu}, \boldsymbol{L}) \in \mathcal{E}_j^K$ with $a_{j+1} \le \Delta/\sqrt{2(K-1)}$, we have $\|\boldsymbol{\mu}\boldsymbol{L}^T - (\boldsymbol{\mu}^*)(\boldsymbol{L}^*)^T\|_F = \|(\boldsymbol{\mu}\boldsymbol{P} - \boldsymbol{\mu}^*)(\boldsymbol{L}^*)^T\|_F \le \sqrt{n}\|\boldsymbol{\mu}\boldsymbol{P} - \boldsymbol{\mu}^*\|_F$ for some permutation matrix $\boldsymbol{P} \in \mathcal{S}^{K\times K}$. For a fixed $\boldsymbol{P} \in \mathcal{S}^{K\times K}$, denote $\mathcal{G}_j^K(\boldsymbol{P}) \triangleq \{\boldsymbol{\mu} \in \mathbb{R}^{p\times K} : |\mathrm{supp}(\boldsymbol{\mu})| \le s, \|\boldsymbol{\mu}\boldsymbol{P} - \boldsymbol{\mu}^*\|_F \in (a_j, a_{j+1}/\sqrt{n}]\}, \mathcal{G}_j^K \triangleq \{\boldsymbol{\mu} \in \mathbb{R}^{p\times K} : |\mathrm{supp}(\boldsymbol{\mu})| \le 2s, \|\boldsymbol{\mu}\|_F \in (a_j, a_{j+1}/\sqrt{n}]\}$. Then for every $\boldsymbol{P} \in \mathcal{S}^{K\times K}$, there is an injective

function $f_{\boldsymbol{P}} : \mathcal{G}_j^K(\boldsymbol{P}) \to \mathcal{G}_j^K$ such that $f_{\boldsymbol{P}}(\boldsymbol{\mu}) = \boldsymbol{\mu}\boldsymbol{P} - \boldsymbol{\mu}^*$. Thus f is a bijective function between $\mathcal{G}_j^K(\boldsymbol{P})$ and $R_f(\mathcal{G}_j^K(\boldsymbol{P}))$ where $R_f(\mathcal{G}_j^K(\boldsymbol{P})) \subset \mathcal{G}_j^K$ is the image of function f. Note that f is also distance-preserving with respect to the Frobenius norm, i.e., $\|f(\boldsymbol{\mu}) - f(\boldsymbol{\mu}')\|_F = \|\boldsymbol{\mu} - \boldsymbol{\mu}'\|_F$. Thus for any $\eta > 0$, $N(\eta, \mathcal{G}_j^K(\boldsymbol{P}), \|\cdot\|_F) = N(\eta, R_f(\mathcal{G}_j^K(\boldsymbol{P})), \|\cdot\|_F) \leq N(\eta, \mathcal{G}_j^K, \|\cdot\|_F)$. We know that for every $(\boldsymbol{\mu}, \boldsymbol{L}) \in \mathcal{E}_j^K$ such that $a_{j+1} \leq \Delta/\sqrt{2(K-1)}$, there exists $\boldsymbol{P} \in \mathcal{S}^{K\times K}$ such that $\boldsymbol{L}\boldsymbol{P} = \boldsymbol{L}^*$. Suppose $\mathcal{N}_j^K(\boldsymbol{P})$ is a $\zeta/\sqrt{n}$-covering of $\mathcal{G}_j^K(\boldsymbol{P})$. Then there exists $\tilde{\boldsymbol{\mu}} \in \mathcal{N}_j^K(\boldsymbol{P})$ such that $\|\tilde{\boldsymbol{\mu}}\boldsymbol{L} - (\boldsymbol{\mu}^*)(\boldsymbol{L}^*)^T\|_F = \|(\tilde{\boldsymbol{\mu}}\boldsymbol{P} - \boldsymbol{\mu}^*)(\boldsymbol{L}^*)^T\|_F \in (a_j, a_{j+1}]$, i.e., $(\tilde{\boldsymbol{\mu}}, \boldsymbol{L}) \in \mathcal{E}_j^K$, and $\|\boldsymbol{\mu}\boldsymbol{L} - \tilde{\boldsymbol{\mu}}\boldsymbol{L}\|_F \leq \sqrt{n}\|\boldsymbol{\mu} - \tilde{\boldsymbol{\mu}}\|_F \leq \zeta$, which means $\bigcup_{\boldsymbol{P}\in\mathcal{S}^{K\times K}} \mathcal{N}_j^K(\boldsymbol{P})$ is a ζ-covering of $\mathcal{E}_j^K$. Then we have

$$N(a_j\epsilon/2, \mathcal{E}_j^K, \|\cdot\|_F) \leq \sum_{\boldsymbol{P}\in\mathcal{S}^{K\times K}} N(a_j\epsilon/(2\sqrt{n}), \mathcal{G}_j^K(\boldsymbol{P}), \|\cdot\|_F) \leq K! N(a_j\epsilon/(2\sqrt{n}), \mathcal{G}_j^K, \|\cdot\|_F)$$

when $a_{j+1} \leq \Delta/\sqrt{2(K-1)}$. Note that for the covering number of the space $\mathcal{E}_j^K$, we abuse the notation by using $\|\cdot\|_F$ to denote the metric $d((\boldsymbol{\mu}_1, \boldsymbol{L}_1), (\boldsymbol{\mu}_2, \boldsymbol{L}_2)) = \|\boldsymbol{\mu}_1\boldsymbol{L}_1^T - \boldsymbol{\mu}_2\boldsymbol{L}_2^T\|_F$.

Step 4: Reduction of covering numbers of $\mathcal{E}_j^K$'s for large a_{j+1}. Next we consider the case when a_j is relatively large. Specifically, when $a_j \geq 2\|(\boldsymbol{\mu}^*)(\boldsymbol{L}^*)^T\|_F$ and $(\boldsymbol{\mu}, \boldsymbol{L}) \in \mathcal{E}_j^K$, we know that $\|\boldsymbol{\mu}\boldsymbol{L}^T\|_F \in (a_j - \|(\boldsymbol{\mu}^*)(\boldsymbol{L}^*)^T\|_F, a_{j+1} + \|(\boldsymbol{\mu}^*)(\boldsymbol{L}^*)^T\|_F] \subset (a_j/2, 2a_{j+1}]$. For $\mathcal{E}_j^K$, we can write it as $\mathcal{E}_j^K = \bigcup_{m=0}^{K-1} \mathcal{E}_{j,m}^K$ where $\mathcal{E}_{j,m}^K$ is the subset of $\mathcal{E}_j^K$ whose $\boldsymbol{L}$ is induced by a clustering with m empty clusters. Then for $\mathcal{E}_{j,m}^K$, it suffices to consider $\mathcal{F}_{j,m}^K \triangleq \{(\boldsymbol{\mu}_{-m}, \boldsymbol{L}_{-m}) : (\boldsymbol{\mu}, \boldsymbol{L}) \in \mathcal{E}_{j,m}^K\}$ where $\boldsymbol{\mu}_{-m}$ and $\boldsymbol{L}_{-m}$ are the sub-matrices of $\boldsymbol{\mu}$ and $\boldsymbol{L}$ by deleting the columns that correspond to the empty clusters respectively. For those j's with $a_j \geq 2\|(\boldsymbol{\mu}^*)(\boldsymbol{L}^*)^T\|_F$, we further have

$$\begin{aligned}\mathcal{F}_{j,m}^K &\subset \{(\boldsymbol{\mu}_{-m}, \boldsymbol{L}_{-m}) : \|\boldsymbol{\mu}_{-m}\boldsymbol{L}_{-m}^T\|_F \in (\frac{a_j}{2}, 2a_{j+1}], |\text{supp}(\boldsymbol{\mu}_{-m})| \leq s\} \\ &\subset \bigcup_{\boldsymbol{L}_{-m}\in\mathcal{L}_{K-m}} \mathcal{H}_{j,m}^K \times \{\boldsymbol{L}_{-m}\}\end{aligned}$$

because the singular values of $\boldsymbol{L}_{-m}$ are between 1 and $\sqrt{n}$, where

$$\mathcal{H}_{j,m}^K \triangleq \{\boldsymbol{\mu}_{-m} \in \mathbb{R}^{p\times(K-m)} : |\text{supp}(\boldsymbol{\mu}_{-m})| \leq s, \|\boldsymbol{\mu}_{-m}\|_F \in (a_j/2, 2a_{j+1}/\sqrt{n}]\}.$$

Since for any $(\boldsymbol{\mu}_{-m}, \boldsymbol{L}_{-m})$ and $(\boldsymbol{\mu}'_{-m}, \boldsymbol{L}_{-m}) \in \mathcal{H}_{j,m}^K \times \{\boldsymbol{L}_{-m}\}$ we have

$$\|\boldsymbol{\mu}_{-m}\boldsymbol{L}_{-m}^T - \boldsymbol{\mu}'_{-m}\boldsymbol{L}_{-m}^T\|_F \leq \|\boldsymbol{L}_{-m}\|_2\|\boldsymbol{\mu}_{-m} - \boldsymbol{\mu}'_{-m}\|_F \leq \sqrt{n}\|\boldsymbol{\mu}_{-m} - \boldsymbol{\mu}'_{-m}\|_F,$$

it follows that

$$N(a_j\epsilon/2, \mathcal{E}_j^K, \|\cdot\|_F) \leq \sum_{m=0}^{K-1} N(a_j\epsilon/2, \mathcal{F}_{j,m}^K, \|\cdot\|_F) \leq \sum_{m=0}^{K-1} |\mathcal{L}_{K-m}| N(a_j\epsilon/(2\sqrt{n}), \mathcal{H}_{j,m}^K, \|\cdot\|_F).$$

Step 5: Computing covering numbers of $\mathcal{E}_j^K$'s for small and large a_{j+1}. We denote $a_{-1} = \Delta/\sqrt{2(K-1)}$, $a_1 = \Delta/\sqrt{2(K-1)}$, $a_2 = 2\|(\boldsymbol{\mu}^*)(\boldsymbol{L}^*)^T\|_F$, $\mathcal{E}_0^K = \mathcal{E}_{-1}^K = \emptyset$ and

$$\frac{a_{j+1}}{a_j} = \begin{cases} \frac{\sqrt{n}}{4}\left(1 + \frac{1}{j^2}\right)^{\frac{1}{sK}} & \text{for } j > 2 \\ \sqrt{n}\left(1 + \frac{1}{(-j-1)^2}\right)^{\frac{1}{2sK}} & \text{for } j < -1 \end{cases}.$$

Note that without loss of generality we may assume $a_2 > a_1$. We have $a_{j+1}/\sqrt{n} \geq a_j$ and

$$\lim_{j\to\infty} a_j = \lim_{j\to\infty} \left(\frac{n}{16}\right)^{\frac{j-1}{2}} \left(\prod_{i=1}^{j-1}\left(1+\frac{1}{i^2}\right)\right)^{\frac{1}{sK}} 2\|(\boldsymbol{\mu}^*)(\boldsymbol{L}^*)^T\|_F = \infty,$$

$$\lim_{j\to\infty} a_{-j} = \lim_{j\to\infty} n^{-\frac{j-1}{2}} \left(\prod_{i=1}^{j-1}\left(1+\frac{1}{i^2}\right)\right)^{-\frac{1}{2sK}} \frac{\Delta}{\sqrt{2(K-1)}} = 0.$$

For $j > 2$, we have

$$N\left(\frac{a_j\epsilon}{2\sqrt{n}}, \mathcal{H}_{j,m}^K, \|\cdot\|_F\right) \leq \binom{p}{s}\left(\frac{3\sqrt{n}}{a_j\epsilon}\right)^{s(K-m)} \left\{\left(\frac{4a_{j+1}}{\sqrt{n}}\right)^{s(K-m)} - a_j^{s(K-m)}\right\}$$
$$\leq \binom{p}{s}\left(\frac{3\sqrt{n}}{\epsilon}\right)^{sK}\left\{\left(\frac{4a_{j+1}}{a_j\sqrt{n}}\right)^{sK} - 1\right\} \leq \binom{p}{s}\left(\frac{3\sqrt{n}}{\epsilon}\right)^{sK}\frac{1}{j^2}.$$

Then

$$\sum_{j=2}^{\infty} N\left(\frac{a_j\epsilon}{2}, \mathcal{E}_j^K, \|\cdot\|_F\right) \leq \sum_{j=1}^{\infty}\sum_{m=0}^{K-1} |\mathcal{L}_{K-m}| N\left(\frac{a_j\epsilon}{2\sqrt{n}}, \mathcal{H}_{j,m}^K, \|\cdot\|_F\right) \leq K^{n+1}\binom{p}{s}\left(\frac{3\sqrt{n}}{\epsilon}\right)^{sK}\frac{\pi^2}{6}.$$

Similarly,

$$\sum_{j=-\infty}^{-1} N\left(\frac{a_j\epsilon}{2}, \mathcal{E}_j^K, \|\cdot\|_F\right) \leq \sum_{j=-\infty}^{-1} K! N\left(\frac{a_j\epsilon}{2\sqrt{n}}, \mathcal{G}_j^K, \|\cdot\|_F\right) \leq K!\binom{p}{2s}\left(\frac{6\sqrt{n}}{\epsilon}\right)^{2sK}\frac{\pi^2}{6}.$$

Step 6: Computing the covering number of $\mathcal{E}_1^K$. Denote $\mathcal{F}_{1,m}^K(\boldsymbol{L}) \triangleq \{(\boldsymbol{\mu}_{-m}, \boldsymbol{L}_{-m}) : (\boldsymbol{\mu}_{-m}, \boldsymbol{L}_{-m}) \in \mathcal{E}_{1,m}^K\}$ for fixed $\boldsymbol{L}$ which induces m empty clusters. Then we have $\mathcal{F}_{1,m}^K = \bigcup_{\boldsymbol{L}\in\mathcal{L}_{K-m}} \mathcal{F}_{1,m}^K(\boldsymbol{L})$. By the previous derivation we have that $\|\boldsymbol{\mu}_{-m}\boldsymbol{L}_{-m}^T - (\boldsymbol{\mu}^*)(\boldsymbol{L}^*)^T\|_F^2 \leq a_{j+1}^2$ is equivalent to $\sum_{g=1}^{K-m} n_g \left\|\boldsymbol{\mu}_g - \sum_{h=1}^K n_{gh}\boldsymbol{\mu}_h^*/n_g\right\|_2^2 + C_{\boldsymbol{L}} \leq a_{j+1}^2$.

Denote $(\boldsymbol{\mu}_h^*)^{\mathrm{supp}(\boldsymbol{\mu}^*)\setminus S}, (\boldsymbol{\mu}_h^*)^{\mathrm{supp}(\boldsymbol{\mu}^*)\cap S} \in \mathbb{R}^p$ as the vectors which have the same values as $\boldsymbol{\mu}_h^*$ on coordinates $\mathrm{supp}(\boldsymbol{\mu}^*) \setminus S$ and $\mathrm{supp}(\boldsymbol{\mu}^*) \cap S$ respectively, and 0 elsewhere. Then, for S which is the support of $\boldsymbol{\mu}$, we have $\boldsymbol{\mu}_h^* = (\boldsymbol{\mu}_h^*)^{\mathrm{supp}(\boldsymbol{\mu}^*)\setminus S} + (\boldsymbol{\mu}_h^*)^{\mathrm{supp}(\boldsymbol{\mu}^*)\cap S}$ and $\langle \boldsymbol{\mu}_g, (\boldsymbol{\mu}_h^*)^{\mathrm{supp}(\boldsymbol{\mu}^*)\setminus S}\rangle = \langle (\boldsymbol{\mu}_h^*)^{\mathrm{supp}(\boldsymbol{\mu}^*)\cap S}, (\boldsymbol{\mu}_h^*)^{\mathrm{supp}(\boldsymbol{\mu}^*)\setminus S}\rangle = 0$. Thus, for $\mathcal{E}_1^K$ we have

$$\sum_{g=1}^{K-m} n_g \left\|\boldsymbol{\mu}_g - \frac{\sum_{h=1}^K n_{gh}(\boldsymbol{\mu}_h^*)^{\mathrm{supp}(\boldsymbol{\mu}^*)\cap S}}{n_g}\right\|_2^2 + \sum_{g=1}^{K-m} n_g \left\|\frac{\sum_{h=1}^K n_{gh}(\boldsymbol{\mu}_h^*)^{\mathrm{supp}(\boldsymbol{\mu}^*)\setminus S}}{n_g}\right\|_2^2 + C_{\boldsymbol{L}} \leq a_2^2.$$

We then denote $C_{\boldsymbol{L}}' \triangleq C_{\boldsymbol{L}} + \sum_{g=1}^{K-m} n_g \left\|\frac{\sum_{h=1}^K n_{gh}(\boldsymbol{\mu}_h^*)^{\mathrm{supp}(\boldsymbol{\mu}^*)\setminus S}}{n_g}\right\|_2^2$.

Denote $\mathcal{F}_{1,m,S}^K(\boldsymbol{L}) \triangleq \{(\boldsymbol{\mu}_{-m}, \boldsymbol{L}_{-m}) \in \mathcal{F}_{1,m}^K(\boldsymbol{L}) : \mathrm{supp}(\boldsymbol{\mu}) = S, |S| \leq s\}$ for fixed $S \subset [p]$. Then we have $\mathcal{F}_{1,m}^K(\boldsymbol{L}) = \bigcup_{S\subset[p],|S|\leq s} \mathcal{F}_{1,m,S}^K(\boldsymbol{L})$ and therefore

$$N(a_1\epsilon/2, \mathcal{E}_1^K, \|\cdot\|_F) \leq \sum_{m=0}^{K}\sum_{\boldsymbol{L}\in\mathcal{L}_{K-m}}\sum_{S\subset[p],|S|\leq s} N(a_1\epsilon/2, \mathcal{F}_{1,m,S}^K(\boldsymbol{L}), \|\cdot\|_F).$$

Let $\mathcal{I}_{1,m,S}^K(\boldsymbol{L}) \overset{\Delta}{=} \left\{(\boldsymbol{\mu}, \boldsymbol{L}_{-m}) : \sum_{g=1}^{K-m} n_g \|\boldsymbol{\mu}_g\|_2^2 + C'_{\boldsymbol{L}} \le a_2^2, \text{supp}(\boldsymbol{\mu}) = S, |S| \le s\right\}$ for fixed $\boldsymbol{L}$ which induces m empty clusters. Note that there is an injective function $f : \mathcal{F}_{1,m,S}^K(\boldsymbol{L}) \to \mathcal{I}_{1,m,S}^K(\boldsymbol{L})$ such that $f(\boldsymbol{\mu}_g) = \boldsymbol{\mu}_g - \sum_{h=1}^K n_{gh} (\boldsymbol{\mu}_h^*)^{\text{supp}(\boldsymbol{\mu}^*)\cap S}/n_g$ for $g \in [K-m]$ and we know that $\mathcal{I}_{1,m,S}^K(\boldsymbol{L})$ is contained in an $s(K-m)$-dimensional ellipsoid with center 0 and length of semi-axes $\{(a_2^2 - C'_{\boldsymbol{L}})/n_g\}_{g=1}^{K-m}$. Thus the volume of $\mathcal{F}_{1,m,S}^K(\boldsymbol{L})$ can be bounded

$$|\mathcal{F}_{1,m,S}^K(\boldsymbol{L})| \le |\mathcal{I}_{1,m,S}^K(\boldsymbol{L})| \le \frac{\pi^{\frac{s(K-m)}{2}}}{\Gamma\left(\frac{s(K-m)}{2}+1\right)} \prod_{g=1}^{K-m} \frac{(a_2^2 - C'_{\boldsymbol{L}})^{s/2}}{n_g^{s/2}} \le \frac{\pi^{\frac{s(K-m)}{2}}}{\Gamma\left(\frac{s(K-m)}{2}+1\right)} \frac{a_2^{s(K-m)}}{\prod_{g=1}^{K-m} n_g^{s/2}}$$

where Γ is the Euler's Gamma function.

Suppose $\mathcal{M}_{1,m,S}^K(\boldsymbol{L})$ is a maximal $a_1\epsilon/2$-packing of $\mathcal{F}_{1,m,S}^K(\boldsymbol{L})$ for fixed $\boldsymbol{L}$ and $S \subset [p]$. Then for every $(\tilde{\boldsymbol{\mu}}_{-m}, \boldsymbol{L}_{-m}) \in \mathcal{M}_{1,m,S}^K(\boldsymbol{L})$, consider $U_{1,m,S}((\tilde{\boldsymbol{\mu}}_{-m}, \boldsymbol{L}_{-m}), a_1\epsilon/4) \subset \mathcal{F}_{1,m,S}^K$. We have $\|\boldsymbol{\mu}\boldsymbol{L}^T - \tilde{\boldsymbol{\mu}}\boldsymbol{L}^T\|_F^2 = \sum_{g=1}^{K-m} n_g \|\boldsymbol{\mu}_g - \tilde{\boldsymbol{\mu}}_g\|_2^2 \le a_1^2\epsilon^2/16$. Let $\mathcal{U}_{1,m,S}(\boldsymbol{L}) = \left\{(\boldsymbol{\mu}, \boldsymbol{L}_{-m}) \in \mathbb{R}^{p\times(K-m)} \times \mathcal{L}_{K-m} : \sum_{g=1}^{K-m} 16 n_g \|\boldsymbol{\mu}_g\|_2^2/(a_1^2\epsilon^2) \le 1, \text{supp}(\boldsymbol{\mu}) = S, |S| \le s\right\}$ for fixed $\boldsymbol{L}$ and $S \subset [p]$. Since $\boldsymbol{\mu}$ shares the same support as $\tilde{\boldsymbol{\mu}}_{-m}$ for $(\boldsymbol{\mu}, \boldsymbol{L}_{-m}) \in U_{1,m,S}((\tilde{\boldsymbol{\mu}}_{-m}, \boldsymbol{L}_{-m}), a_1\epsilon/4)$, there exists an bijective function $f' : U_{1,m,S}((\tilde{\boldsymbol{\mu}}, \boldsymbol{L}), a_1\epsilon/4) \to \mathcal{U}_{1,m,S}(\boldsymbol{L})$ such that $f'(\boldsymbol{\mu}_g) = \boldsymbol{\mu}_g - \tilde{\boldsymbol{\mu}}_g$ for $g \in [K-m]$. In addition, we know that $\mathcal{U}_{1,m,S}(\boldsymbol{L})$ is essentially an $s(K-m)$-dimensional ellipsoid with center 0 and length of semi-axes $\{(a_1^2\epsilon^2)/16n_g\}_{g=1}^{K-m}$. Therefore, the volume $|U_{1,m,S}((\tilde{\boldsymbol{\mu}}, \boldsymbol{L}), \frac{a_1\epsilon}{4})| = \frac{\pi^{\frac{s(K-m)}{2}}}{\Gamma\left(\frac{s(K-m)}{2}+1\right)} \prod_{g=1}^{K-m} \left(\frac{a_1\epsilon}{4\sqrt{n_g}}\right)^s$. Note that the sets $U_{1,m,S}\left((\tilde{\boldsymbol{\mu}}_{-m}, \boldsymbol{L}_{-m}), \frac{a_1\epsilon}{4}\right)$ are disjoint when $\tilde{\boldsymbol{\mu}}_{-m}$ varies since $\mathcal{M}_{1,m,S}^K(\boldsymbol{L})$ is a packing. Then we have,

$$\begin{aligned} N\left(\frac{a_1\epsilon}{2}, \mathcal{F}_{1,m,S}(\boldsymbol{L}), \|\cdot\|_F\right) &\le |\mathcal{M}_{1,m,S}^K(\boldsymbol{L})| \le \frac{|\mathcal{F}_{1,m}^K|}{|U_{1,m,S}((\tilde{\boldsymbol{\mu}}, \boldsymbol{L}), a_1\epsilon/4)|} \\ &\le \frac{\pi^{\frac{s(K-m)}{2}}/\Gamma\left(\frac{s(K-m)}{2}+1\right)}{\pi^{\frac{s(K-m)}{2}}/\Gamma\left(\frac{s(K-m)}{2}+1\right)} \left(\frac{2a_2}{\epsilon a_1}\right)^{s(K-m)} \le \left(\frac{2}{\epsilon}\right)^{sK} \left(\frac{2\|(\boldsymbol{\mu}^*)(\boldsymbol{L}^*)^T\|_F}{\Delta}\right)^{sK}. \end{aligned}$$

Therefore,

$$\begin{aligned} N(a_1\epsilon/2, \mathcal{E}_1^K, \|\cdot\|_F) &\le \sum_{m=0}^{K-1} \sum_{\boldsymbol{L}\in\mathcal{L}_{K-m}} \sum_{S\in[p],|S|\le s} |\mathcal{M}_{1,m,S}^K(\boldsymbol{L})| \\ &\le KK^n \binom{p}{s} \left(\frac{2}{\epsilon}\right)^{sK} \left(\frac{2\|(\boldsymbol{\mu}^*)(\boldsymbol{L}^*)^T\|_F}{\Delta}\right)^{sK} \lesssim KK^n \binom{p}{s} \left(\frac{2}{\epsilon}\right)^{sK} (sn)^{sK/2}. \end{aligned}$$

Note that $\Delta \ge 1/n^q$ and $\|(\boldsymbol{\mu}^*)(\boldsymbol{L}^*)^T\|_F^2 = O(sn)$ by assumptions. Then we have

$$\log\left(\sum_{j=-\infty}^{\infty} N\left(\frac{a_j\epsilon}{2}, \mathcal{E}_j^K, \|\cdot\|_F\right)\right) \lesssim n\log K + s\log\frac{p}{s} + sK\log n + sK\log\frac{6}{\epsilon}.$$

Therefore, by Corollary 8.5 in Kosorok (2008), we have

$$\mathbb{E}_* \left[\sup_{(\widehat{\boldsymbol{\mu}}, \widehat{\boldsymbol{L}}) \in \Theta_K} \left\langle \boldsymbol{E}, (\boldsymbol{\Sigma}^*)^{\frac{1}{2}} \frac{\widehat{\boldsymbol{\mu}} \widehat{\boldsymbol{L}}^T - (\boldsymbol{\mu}^*)(\boldsymbol{L}^*)^T}{\|\widehat{\boldsymbol{\mu}} \widehat{\boldsymbol{L}}^T - (\boldsymbol{\mu}^*)(\boldsymbol{L}^*)^T\|_F} \right\rangle_F \right] \lesssim \int_0^{\mathrm{diam}(\widetilde{\Theta}_K)} \sqrt{\log N(\epsilon, \widetilde{\Theta}_K, \|\cdot\|_F)} \mathrm{d}\epsilon$$

$$\lesssim \int_0^2 \sqrt{\log K + s \log \frac{p}{s} + n \log K + \frac{sK}{2} \log n + sK \log \frac{6\lambda_{\max}(\boldsymbol{\Sigma}^*)^{\frac{1}{2}}}{\epsilon}} d\epsilon$$

$$\leq \sqrt{s \log \frac{p}{s} + n \log K + sK \log n} + 2\sqrt{sK} \int_0^1 \sqrt{-\log u + \log(12\lambda_{\max}(\boldsymbol{\Sigma}^*)^{\frac{1}{2}})} du$$

$$\lesssim \sqrt{s \log \frac{p}{s} + n \log K}.$$

■

Appendix B. Proofs for Section 3

B.1 Proof architecture

We first sketch the proof of Theorem 4 by providing technical lemmas below.

Lemma 11 *Under the conditions of Theorem 4, we have*

$$\Pi\{\|\boldsymbol{\mu} \boldsymbol{L}^T - (\boldsymbol{\mu}^*)(\boldsymbol{L}^*)^T\|_F^2 < s \log p\} \geq \exp\{-c(s \log p + n \log K^*)\}.$$

Note that the prior of $\boldsymbol{\mu}$ is absolutely continuous with respect to the Lebesgue measure, which implies $|\mathrm{supp}(\boldsymbol{\mu})| = p$ with probability 1. However, we expect most rows of $\boldsymbol{\mu}$ come from the "spike" distribution *a priori*, which implies the "magnitude" of these rows is quite small with high prior probability. This motivates us to define a generalized notation of the support. Formally, for $\delta > 0$, we define $\mathrm{supp}_\delta(\boldsymbol{\mu}) \triangleq \{j \in [p] : \|\boldsymbol{\mu}_{j*}\|_1 \leq \delta\}$ as the soft support of $\boldsymbol{\mu}$ with threshold δ, where $\boldsymbol{\mu}_{j*}$ represents the jth row of $\boldsymbol{\mu}$. Let $(\boldsymbol{\mu}_k)_{S_\delta} = (\mu_{jk} : j \in \mathrm{supp}_\delta(\boldsymbol{\mu})) \in \mathbb{R}^{|\mathrm{supp}_\delta(\boldsymbol{\mu})|}$ denotes the sub-vector of $\boldsymbol{\mu}_k$ whose coordinates are in $\mathrm{supp}_\delta(\boldsymbol{\mu})$. It is conceivable that for small δ, the size of the soft support of $\boldsymbol{\mu}$ is small compared with p with high prior probability. This heuristics is formalized through the following lemma.

Lemma 12 *Given K, suppose $\boldsymbol{\mu} \in \mathbb{R}^{p \times K}$ follows the prior specification (4) and (8) with some hyperparameters $\kappa > 0$, $\alpha > 1$, $\lambda_0 \gg \lambda_1 > 0$ and let $\delta = (1 + \kappa) \log p / \lambda_0$. Assume $K \log \log p \leq \log p$. Then given K, we have, for $\boldsymbol{\mu} \in \mathbb{R}^{p \times K}$,*

$$\Pi\left(|\mathrm{supp}_\delta(\boldsymbol{\mu})| \geq \beta\left(s + \frac{n \log K}{\log p}\right)\right) \leq \exp\left(-c(s \log p + n \log K)\right)$$

for some constants $\beta, c > 0$.

Lemma 13 *Let $(\boldsymbol{\mu}', \boldsymbol{L}') \in \Theta = \bigcup_{K=1}^{K_{\max}} \mathbb{R}^{p \times K} \times \mathcal{L}_K$ be such that $(\boldsymbol{\mu}')(\boldsymbol{L}')^T \neq (\boldsymbol{\mu}^*)(\boldsymbol{L}^*)^T$ and consider*

$$\mathcal{E} = \{(\boldsymbol{\mu}, \boldsymbol{L}) : \|\boldsymbol{\mu} \boldsymbol{L}^T - (\boldsymbol{\mu}')(\boldsymbol{L}')^T\|_F \leq \delta \|(\boldsymbol{\mu}')(\boldsymbol{L}')^T - (\boldsymbol{\mu}^*)(\boldsymbol{L}^*)^T\|_F\},$$

for some sufficiently small constant δ such that $0 < \delta < \|\boldsymbol{\Sigma}^*\|_2/(2\|\boldsymbol{\Sigma}^*\|_2 + 4)$. Assume the conditions of Theorem 4 hold. Let $p_0(\boldsymbol{Y}_i)$ and $p^*(\boldsymbol{Y}_i)$ be the density functions of $N(((\boldsymbol{\mu}^*)(\boldsymbol{L}^*)^T)_i, \boldsymbol{I}_p)$ and $N(((\boldsymbol{\mu}^*)(\boldsymbol{L}^*)^T)_i, \boldsymbol{\Sigma}^*)$ respectively. Denote $p_0(\boldsymbol{Y}) = \prod_{i=1}^n p_0(\boldsymbol{Y}_i)$ and $p^*(\boldsymbol{Y}) = \prod_{i=1}^n p^*(\boldsymbol{Y}_i)$. Then there exists a test function ϕ_n such that

$$\mathbb{E}_* \phi_n \leq \exp\{-c_1\|(\boldsymbol{\mu}')(\boldsymbol{L}')^T - (\boldsymbol{\mu}^*)(\boldsymbol{L}^*)^T\|_F^2\},$$
$$\sup_{(\boldsymbol{\mu},\boldsymbol{L})\in\mathcal{E}} \mathbb{E}_{(\boldsymbol{\mu},\boldsymbol{L},\boldsymbol{I}_p)}\left(\frac{p^*(\boldsymbol{Y})}{p_0(\boldsymbol{Y})}(1-\phi_n)\right) \leq \exp\left\{-c_2\|(\boldsymbol{\mu}')(\boldsymbol{L}')^T - (\boldsymbol{\mu}^*)(\boldsymbol{L}^*)^T\|_F^2\right\}$$

where $c_1, c_2 > 0$ are some positive constants that are independent of n.

Lemma 14 *Let*

$$\mathcal{F}_n = \bigcup_{K=1}^{K_{\max}} \left\{\boldsymbol{\mu}\boldsymbol{L}^T : \boldsymbol{\mu} \in \mathbb{R}^{p\times K}, |\mathrm{supp}_\delta(\boldsymbol{\mu})| \leq \beta\left(s + \frac{n\log K}{\log p}\right), \max_{k\in[K]} \|(\boldsymbol{\mu}_k)_{S_\delta}\|_\infty \leq a_n, \boldsymbol{L} \in \mathcal{L}_K\right\}$$

where β and δ are defined as in Lemma 12 and $a_n = (s\log p + n\log K_{\max})n^\gamma$ for some constant $\gamma > 0$. Denote $N(\epsilon_n, \mathcal{F}_n, d)$ as the covering number of $\mathcal{F}_n$ with respect to the metric $d(\boldsymbol{A},\boldsymbol{B}) = \|\boldsymbol{A}-\boldsymbol{B}\|_F/\sqrt{n}$. Suppose $\epsilon_n^2 = (s\log p + n\log K_{\max})/n$. Then we have, for some constant c,

$$N(\epsilon_n, \mathcal{F}_n, d) \leq \exp(cn\epsilon_n^2).$$

Lemma 15 *Let δ be defined as in Lemma 12 and $\mathcal{F}_n$ be defined as in Lemma 14. Assume the conditions of Theorem 4 hold. Then we have, for some constant c,*

$$\Pi(\mathcal{F}_n^c) \leq \exp\{-c(s\log p + n\log K^*)\}.$$

B.2 Proofs of the auxiliary lemmas

In this subsection, we provide the detailed proofs of the lemmas appearing in Section B.1.
Proof of Lemma 11 The proof of this lemma is based on a mofidication of that of Lemma 3.1 in Xie et al. (2022). Denote $\epsilon^2 = (s\log p)/n$. First by conditioning on the event $\{(\boldsymbol{\mu},\boldsymbol{L}) \in \Theta : \boldsymbol{L} = \boldsymbol{L}^*, K = K^*\}$, we have that

$$\Pi\{\|\boldsymbol{\mu}\boldsymbol{L}^T - (\boldsymbol{\mu}^*)(\boldsymbol{L}^*)^T\|_F^2 < n\epsilon^2 \mid \boldsymbol{L} = \boldsymbol{L}^*, K = K^*\} \geq \Pi\left(\bigcap_{k=1}^{K^*}\left\{\|\boldsymbol{\mu}_k - \boldsymbol{\mu}_k^*\|_2^2 \leq \epsilon^2\right\}\right),$$

where n_k^* is the number of observations assigned to the kth cluster according to the cluster assignment matrix $\boldsymbol{L}^*$. Now we focus on the prior distribution of $\boldsymbol{\mu}_k$. Denote S_0 the true sparsity of $\boldsymbol{\mu}^*$. Note that for each $k \in [K^*]$, $\|\boldsymbol{\mu}_k - \boldsymbol{\mu}_k^*\|_2 \leq \|(\boldsymbol{\mu}_k)_{S_0} - (\boldsymbol{\mu}_k^*)_{S_0}\|_2 + \|(\boldsymbol{\mu}_k)_{S_0^c}\|_2$,

$$\mathbb{P}\left(\bigcap_{k=1}^{K^*}\left\{\|\boldsymbol{\mu}_k - \boldsymbol{\mu}_k^*\|_2^2 < \epsilon^2\right\}\right) \geq \Pi\left(\bigcap_{k=1}^{K^*}\left\{\|(\boldsymbol{\mu}_k)_{S_0} - (\boldsymbol{\mu}_k^*)_{S_0}\|_2 < \frac{\epsilon}{2}\right\} \cap \left\{\|(\boldsymbol{\mu}_k)_{S_0^c}\|_2 < \frac{\epsilon}{2}\right\}\right).$$

Now we introduce the latent random variable $\xi_j \sim \mathrm{Bernoulli}(\theta)$ such that $|\mu_{jk}| \mid \xi_j \sim (1-\xi_j)\mathrm{Exp}(\lambda_0) + \xi_j\mathrm{Exp}(\lambda_1)$ independently for all $j \in [p]$ and $k \in [K^*]$, where μ_{jk} is the

jth coordinate of $\boldsymbol{\mu}_k$. Recall that a Laplace distribution can be represented as a scale-mixture of normals as $(\mu_{jk} \mid \phi_j, \xi_j) \sim N\left(0, \phi_j/\lambda_{\xi_j}^2\right)$ with $\phi_j \sim \text{Exp}\,(1/2)$. Define the event $\mathcal{A} = \bigcap_{j\in S_0}\{\xi_j = 1\}\bigcap_{j\in S_0^c}\{\xi_j = 0\}\bigcap_{j\in S_0}\{1 < \phi_j < 4\}$. Note that given $\mathcal{A}$, the entries of $\boldsymbol{\mu}$ are independent. Conditioning on $\mathcal{A}$, we have $|\mu_{jk}| \sim \text{Exp}(\lambda_0)$ for $j \in S_0^c$, which implies

$$\prod_{k=1}^{K^*}\Pi\left(\|(\boldsymbol{\mu}_k)_{S_0^c}\|_2 < \frac{\epsilon}{2} \,\Big|\, \mathcal{A}\right) \geq \prod_{k=1}^{K^*}\prod_{j\in S_0^c}\Pi\left(|\mu_{jk}| < \frac{\epsilon}{2\sqrt{p}} \,\Big|\, \mathcal{A}\right)$$
$$= \left\{1 - \exp\left(-\frac{\lambda_0\epsilon}{2\sqrt{p}}\right)\right\}^{K^*(p-s)} \geq \left(1 - \frac{s}{p}\right)^{pK^*} \geq \exp\{-\log(2e)sK^*\}.$$

Here we use the inequality $(1-x)^{1/x} \geq 1/(2e)$ for $x \in (0, 1/2)$ and the fact $\lambda_0 \geq 2\log\frac{p}{s}\sqrt{\frac{np}{s\log p}} \implies \frac{\lambda_0}{2}\sqrt{\frac{s\log p}{np}} \geq \log\frac{p}{s}$. Next, by Anderson's lemma (see Lemma 1.4 in supporting document for Pati et al. (2014)), for each $k \in [K^*]$, conditioning on $\mathcal{A}$ (which guarantees that $1 \leq \phi_j \leq 4$ for all $j \in S_0$), we have

$$\Pi\left(\|(\boldsymbol{\mu}_k)_{S_0} - (\boldsymbol{\mu}_k^*)_{S_0}\|_2 < \frac{\epsilon}{2} \,\Big|\, \mathcal{A}\right) \geq \exp\left(-\frac{1}{2}\sum_{j\in S_0}\frac{|\mu_{jk}^*|^2\lambda_1^2}{\phi_j}\right)\Pi\left(\|(\boldsymbol{\mu}_k)_{S_0}\|_2 < \frac{\epsilon}{2} \,\Big|\, \mathcal{A}\right)$$
$$\geq \exp\left(-\frac{1}{2}\sum_{j\in S_0}\frac{|\mu_{jk}^*|^2\lambda_1^2}{\phi_j}\right)\prod_{j\in S_0}\left\{2\Phi\left(\frac{\epsilon\lambda_1}{2\sqrt{s\phi_j}}\right) - 1\right\}$$
$$\geq \exp\left(-\frac{1}{2}\lambda_1^2\|(\boldsymbol{\mu}_k^*)_{S_0}\|_2^2\right)\left\{2\Phi\left(\frac{\epsilon\lambda_1}{4\sqrt{s}}\right) - 1\right\}^s \geq \exp\left\{-\frac{1}{2}\lambda_1^2\|\boldsymbol{\mu}_k^*\|_2^2 - s\left(1 + \left|\log\frac{\epsilon\lambda_1}{4\sqrt{s}}\right|\right)\right\},$$

where we use $\log(2\Phi(x) - 1) \geq -1 - |\log x|$ for small $x > 0$ in the last inequality. Since

$$sK^*\left|\log\frac{\epsilon\lambda_1}{4\sqrt{s}}\right| = sK^*\left|\log\left(\frac{\lambda_1}{4}\sqrt{\frac{\log p}{n}}\right)\right|$$
$$\leq sK^*\left|\log\frac{\lambda_1}{4}\right| + \frac{sK^*}{2}\left|\log\frac{\log p}{n}\right| \leq c_1' sK^*\log n \leq c_1 s\log p$$

for some constants $c_1', c_1 > 0$, it follows that $\prod_{k=1}^{K^*}\Pi\left(\|\boldsymbol{\mu}_k - \boldsymbol{\mu}_k^*\|_2^2 < \epsilon \,\Big|\, \mathcal{A}\right) \geq \exp(-c_2 s\log p)$ for some constant $c_2 \geq \max(c_1, C) > 0$ given $\sum_{k=1}^{K^*}\lambda_1^2\|\boldsymbol{\mu}_k^*\|_2^2 \leq Cs\log p$. We next consider the prior probability of the event $\mathcal{A}$. First note that $\prod_{j\in S_0}\Pi(1 < \phi_j < 4) \geq \exp(-c_3 s)$ for some constant $c_3 > 0$ by the definition of the exponential distribution. Since the prior of θ is Beta$(1, \beta_\theta)$ where $\beta_\theta = p^{1+\kappa}\log p$, for some constant $c_4 > 1 + \kappa > 0$,

$$\Pi\left(\bigcap_{i\in S_0}\{\xi_i = 1\}\bigcap_{i\in S_0^c}\{\xi_i = 0\}\right) = \int_0^1\theta^s(1-\theta)^{p-s}\Pi(d\theta)$$
$$= \frac{\Gamma(\beta_\theta + 1)}{\Gamma(\beta_\theta)}\int_0^1\theta^s(1-\theta)^{p+\beta_\theta-s}d\theta = \frac{\Gamma(s+1)\Gamma(p + \beta_\theta - s)}{\Gamma(p + \beta_\theta + 1)}\frac{\Gamma(\beta_\theta + 1)}{\Gamma(\beta_\theta)}$$
$$\geq \exp\left(-s\log(p + \beta_\theta) + \log\beta_\theta\right) \geq \exp(-s\log(2\beta_\theta))$$
$$\geq \exp\left(-s\log\log p - (1+\kappa)s\log p - s\log 2\right) \geq \exp(-c_4 s\log p).$$

Hence, we obtain that $\Pi(\mathcal{A}) \geq \exp(-c_3 s - c_4 s \log p)$ and therefore,

$$\Pi\left(\bigcap_{k=1}^{K^*}\left\{\|\boldsymbol{\mu}_k - \boldsymbol{\mu}_k^*\|_2^2 < \frac{\epsilon^2}{K^*}\right\}\right) \geq \prod_{k=1}^{K^*}\Pi\left(\|\boldsymbol{\mu}_k - \boldsymbol{\mu}_k^*\|_2 < \frac{\epsilon}{\sqrt{K^*}} \;\middle|\; \mathcal{A}\right)\Pi(\mathcal{A}) \geq \exp(-c_5 s \log p)$$

for some constant $c_5 > \max(c_2, c_4) > 0$. Thus, we have

$$\Pi\left(\|\boldsymbol{\mu}\boldsymbol{L}^T - (\boldsymbol{\mu}^*)(\boldsymbol{L}^*)^T\|_F^2 \leq s \log p \mid \boldsymbol{L} = \boldsymbol{L}^*, K = K^*\right) \geq \exp(-c_5 s \log p).$$

Next consider $\Pi(\boldsymbol{L} = \boldsymbol{L}^* \mid K = K^*)$ for Multinomial-Dirichlet model. Let $\boldsymbol{w} = (w_1, \ldots, w_{K^*})^T$. Define integers $\alpha_r = \lfloor \alpha \rfloor$ and $\beta_r = \lfloor K^*\alpha \rfloor$. Then we know $\lceil \alpha \rceil = \alpha_r + 1$ and $\lceil K^*\alpha \rceil = \beta_r + 1$. Note that the gamma function $\Gamma(x)$ is strictly increasing for $x > 2$ and we have $\Gamma(x) \leq 1$ for $1 \leq x \leq 2$. Thus, we have, for $K^*\alpha > 2$,

$$\frac{\Gamma(K^*\alpha)}{\Gamma(K^*\alpha + n)} \geq \frac{\Gamma(\beta_r)}{\Gamma(\beta_r + 1 + n)} = \frac{(\beta_r - 1)!}{(\beta_r + n)!};$$

for $1 \leq K^*\alpha \leq 2$,

$$\frac{\Gamma(K^*\alpha)}{\Gamma(K^*\alpha + n)} \geq \frac{\min_{x\in[1,2]}\Gamma(x)}{(\beta_r + n)!} = \left(\min_{x\in[1,2]}\Gamma(x)\right)\frac{\beta_r!}{(\beta_r + n)!}.$$

Similarly, we also have, for $\alpha > 2$,

$$\frac{\Gamma(\alpha + n_i)}{\Gamma(\alpha)} \geq \frac{\Gamma(\alpha_r + n_i)}{\Gamma(\alpha_r + 1)} \geq \frac{(\alpha_r + n_i - 1)!}{\alpha_r!};$$

for $1 \leq \alpha \leq 2$,

$$\frac{\Gamma(\alpha + n_i)}{\Gamma(\alpha)} \geq \frac{(\alpha_r + n_i - 1)!}{1} = \frac{(\alpha_r + n_i - 1)!}{\alpha_r!}.$$

Therefore, we conclude that for $\alpha \geq 1$ and some constants $C_1, C_2 > 0$,

$$\frac{\Gamma(K^*\alpha)}{\Gamma(K^*\alpha + n)} \geq C_1\frac{(\beta_r - 1)!}{(\beta_r + n)!} \quad \text{and} \quad \frac{\Gamma(\alpha + n_i)}{\Gamma(\alpha)} \geq C_2\frac{(\alpha_r + n_i - 1)!}{\alpha_r!}.$$

By integrating out $\boldsymbol{w}$ we have

$$\begin{aligned}
\Pi(\boldsymbol{L} = \boldsymbol{L}^* \mid K = K^*) &= \int \Pi(\boldsymbol{L} = \boldsymbol{L}^* \mid \boldsymbol{w}, K = K^*)d\Pi(\boldsymbol{w} \mid K = K^*) \\
&= \int_0^1 \cdots \int_0^1 \prod_{i=1}^n w_{z_i^*}\frac{\Gamma(K^*\alpha)}{(\Gamma(\alpha))^{K^*}}\prod_{j=1}^{K^*} w_j^{\alpha-1} dw_1 \cdots dw_{K^*} = \frac{\Gamma(K^*\alpha)}{(\Gamma(\alpha))^{K^*}}\frac{\prod_{i=1}^{K^*}\Gamma(\alpha + n_i)}{\Gamma(K^*\alpha + n)} \\
&\geq C_1\frac{(\beta_r - 1)!}{(\beta_r + n)!}(C_2)^{K^*}\prod_{i=1}^{K^*}\frac{(\alpha_r + n_i - 1)!}{\alpha_r!} \\
&= C_1(C_2)^{K^*}\frac{(\beta_r - 1)!}{(\beta_r + n)!}\frac{(K^*\alpha_r + n - K^*)!}{(\alpha_r!)^{K^*}}\frac{1}{\binom{K^*\alpha_r + n - K^*}{\alpha_r + n_1 - 1, \cdots, \alpha_r + n_{K^*} - 1}}
\end{aligned}$$

where $\binom{a}{a_1,\cdots,a_{K^*}} = \frac{a!}{a_1!a_2!\cdots a_{K^*}!}$ is the multinomial coefficient for $a_1 + a_2 + \cdots + a_{K^*} = a$. We know that the function $(a_1,\cdots,a_{K^*}) \mapsto \binom{a}{a_1,\cdots,a_{K^*}}$ achieves the maximum when $a_1,\ldots,a_{K^*}$ are as close as possible to each other. Formally, let $a = qK^* + r$ where $q \triangleq a \mod K^* = \lfloor a/K^* \rfloor$ and $0 \le r < K^*$, then the multinomial coefficient is maximized when $a_1 = \cdots = a_r = q+1$ and $a_{r+1} = \cdots = a_{K^*} = q$, and hence the maximal value is $a!/\{q!^{K^*-r}(q+1)!^r\}$. Then the preceding expression achieves the minimum when $n_1 = \cdots = n_r = \lfloor n/K^* \rfloor + 1$ and $n_{r+1} = \cdots = n_{K^*} = \lfloor n/K^* \rfloor$ where $r = n - \lfloor n/K^* \rfloor K^*$. Note that $\frac{\alpha_r}{\beta_r} = \frac{\lfloor \alpha \rfloor}{\lfloor K^*\alpha \rfloor} \ge \frac{\lfloor \alpha \rfloor}{(\lfloor \alpha \rfloor + 1)K^*} \ge \frac{1}{2K^*}$. So

$$\begin{aligned}
\Pi(\boldsymbol{L} = \boldsymbol{L}^* \mid K = K^*) &\ge C_1(C_2)^{K^*} \frac{\{(\alpha_r+1)(\alpha_r+2)\cdots(\alpha_r + \lfloor \frac{n}{K^*} \rfloor - 1)\}^{K^*}(\lfloor \frac{n}{K^*} \rfloor + \alpha_r)^r}{\beta_r(\beta_r+1)(\beta_r+2)\cdots(\beta_r+n)} \\
&\ge C_1(C_2)^{K^*} \frac{(\alpha_r+1)^{K^*}(\alpha_r+2)^{K^*}\cdots(\alpha_r+\lfloor \frac{n}{K^*} \rfloor - 1)^{K^*}}{(\beta_r+2K^*)^{K^*}(\beta_r+4K^*)^{K^*}\cdots(\beta_r+2\lfloor \frac{n}{K^*} \rfloor K^* - 2K^*)^{K^*}} \times \frac{1}{(\beta_r+n)^{r+K^*}} \\
&\ge C_1(C_2)^{K^*} \left(\frac{1}{2K^*}\right)^{\lfloor \frac{n}{K^*} \rfloor K^*} \times \frac{1}{(\beta_r+n)^{r+K^*}}.
\end{aligned}$$

Also note the prior of K is a truncated Poisson, so we have

$$\Pi(K = K^*) \ge \frac{e^{-\lambda}\lambda^{K^*}}{K^*!} \ge \exp(-\lambda + K^* \log \lambda - K^* \log K^*) \ge \exp(-2K^* \log K^*).$$

Therefore, for some constant $c > 0$,

$$\begin{aligned}
&\Pi\left(\|\boldsymbol{\mu}\boldsymbol{L}^T - (\boldsymbol{\mu}^*)(\boldsymbol{L}^*)^T\|_F^2 \le s\log p\right) \\
&\quad \ge \Pi\left(\|\boldsymbol{\mu}\boldsymbol{L}^T - (\boldsymbol{\mu}^*)(\boldsymbol{L}^*)^T\|_F^2 \le s\log p \mid \boldsymbol{L} = \boldsymbol{L}^*, K = K^*\right)\Pi(\boldsymbol{L} = \boldsymbol{L}^* \mid K = K^*)\Pi(K = K^*) \\
&\quad \ge \exp\{-c(s\log p + n\log K^*)\}.
\end{aligned}$$

■

Proof proof of Lemma 12 We denote $\boldsymbol{\mu}_{j*}$ the jth row of $\boldsymbol{\mu}$ for $j = 1,\ldots,p$. Note that from the prior model, we have $|\mu_{ji}| \mid \xi_j \sim (1-\xi_j)\mathrm{Exp}(\lambda_0) + \xi_j \mathrm{Exp}(\lambda_1)$ where $\mathrm{Exp}(\lambda)$ is the exponential distribution with parameter λ. Then we have $\|\boldsymbol{\mu}_{j*}\|_1 \mid \xi_j \sim (1-\xi_j)\mathrm{Gamma}(K,\lambda_0) + \xi_j \mathrm{Gamma}(K,\lambda_1)$ where $\mathrm{Gamma}(K,\lambda)$ is the Gamma distribution with shape K and rate λ. Thus, by the change of variable $u = \lambda_0 x$ and conditioning on the event $A = \{\theta \le c_1(s + n\log K/\log p)/p^{1+\kappa}\}$ for some constant $c_1 > 0$, we have

$$\begin{aligned}
\Pi\left(\|\boldsymbol{\mu}_{j*}\|_1 > \delta\right) &\le (1-\theta)\int_\delta^\infty \frac{\lambda_0^K}{\Gamma(K)} x^{K-1} e^{-\lambda_0 x} dx + \theta = (1-\theta)\frac{\lambda_0^K}{\Gamma(K)}\frac{1}{\lambda_0^K}\int_{\lambda_0\delta}^\infty u^{K-1}e^{-u}du + \theta \\
&< \frac{1}{\Gamma(K)}(\lambda_0\delta)^K e^{-\lambda_0\delta} + c_1\frac{s + \frac{n\log K}{\log p}}{p^{1+\kappa}} \quad < (\lambda_0\delta)^K e^{-\lambda_0\delta} + c_1\frac{s + \frac{n\log K}{\log p}}{p^{1+\kappa}} \\
&\le \exp(K\log(1+\kappa) + K\log\log p - (1+\kappa)\log p) + c_1\frac{s + \frac{n\log K}{\log p}}{p^{1+\kappa}} \\
&\le \exp(-\kappa\log p) + c_1\frac{s + \frac{n\log K}{\log p}}{p^{1+\kappa}} \le (1+c_1)\frac{s + \frac{n\log K}{\log p}}{p^{1+\kappa}}
\end{aligned}$$

for sufficiently large n. Note that the second inequality comes form the result of Natalini and Palumbo (2000) about upper incomplete gamma function for $\lambda_0\delta = (1+\kappa)\log p > K+1$. The fourth inequality is due to the fact that the function $x \mapsto x^K e^{-x}$ is decreasing when $x > K$. Note that Hagerup and Rüb (1990) stated a version of Chernoff's inequality for binomial distributions that $\mathbb{P}(X > ap) \le \left(\left(\frac{q}{a}\right)^a \exp(a)\right)^p$ if $X \sim \text{Binomial}(p,q)$ and $q \le a < 1$. Then over the event $A = \{\theta \le c_1(s + n\log K/\log p)/p^{1+\kappa}\}$ we have

$$\begin{aligned}
&\Pi\left(|\text{supp}_\delta(\boldsymbol{\mu})| > \beta\left(s + \frac{n\log K}{\log p}\right) \mid A\right)\\
&\le \exp\left(-\beta\left(s+\frac{n\log K}{\log p}\right)\log\frac{\beta\left(s+\frac{n\log K}{\log p}\right)}{pq} + \beta\left(s + \frac{n\log K}{\log p}\right)\right)\\
&\le \exp\left(-\beta\left(s+\frac{n\log K}{\log p}\right)\log(\beta(1+c_1)p^\kappa)\right)\\
&\le \exp\left(-\beta\left(\kappa s\log p + s\log(\beta(1+c_1)) + \kappa n\log K + \frac{n\log K}{\log p}\log(\beta(1+c_1))\right)\right)\\
&\le \exp\left(-c_2(s\log p + n\log K)\right)
\end{aligned}$$

for some constant $0 < c_2 \le \beta\kappa$. For the event A, we calculate the prior probability of A^c. Let $\beta_\theta = p^{1+\kappa}\log p$, we have

$$\begin{aligned}
\Pi\left(\theta > c_1\frac{s\log p + n\log K}{p^{1+\kappa}\log p}\right) &= \int_{c_1\frac{s\log p+n\log K}{p^{1+\kappa}\log p}}^1 \frac{\Gamma(\beta_\theta+1)}{\Gamma(\beta_\theta)}(1-\theta)^{\beta_\theta-1}d\theta\\
&= \left(1 - c_1\frac{s\log p + n\log K}{p^{1+\kappa}\log p}\right)^{\beta_\theta} \le \exp\left(-\beta_\theta c_1\frac{s\log p + n\log K}{p^{1+\kappa}\log p}\right)\\
&\le \exp\left(-c_1(s\log p + n\log K)\right).
\end{aligned}$$

Therefore, for some constant $0 < c \le \min(c_1, c_2)$ we have

$$\begin{aligned}
&\Pi\left(|\text{supp}_\delta(\boldsymbol{\mu})| > \beta\left(s+\frac{n\log K}{\log p}\right)\right) = \int_0^1 \Pi\left(|\text{supp}_\delta(\boldsymbol{\mu})| > \beta\left(s + \frac{n\log K}{\log p}\right) \mid A\right)\Pi(d\theta)\\
&\le \int_0^{c_1\frac{s\log p+n\log K}{p^{1+\kappa}\log p}} \Pi(|\text{supp}_\delta(\boldsymbol{\mu})| > \beta s \mid A)\Pi(d\theta) + \Pi\left(\theta > c_1\frac{s\log p + n\log K}{p^{1+\kappa}\log p}\right)\\
&\le \exp\left(-c(s\log p + n\log K)\right).
\end{aligned}$$

■

Proof of Lemma 13 Construct a likelihood ratio test $\phi_n = \mathbb{1}\{\|\boldsymbol{Y} - (\boldsymbol{\mu}^*)(\boldsymbol{L}^*)^T\|_F^2 - \|\boldsymbol{Y} - (\boldsymbol{\mu}')(\boldsymbol{L}')^T\|_F^2 \ge \rho\}$ where $\rho = (2m-1)\|(\boldsymbol{\mu}^*)(\boldsymbol{L}^*)^T - (\boldsymbol{\mu}')(\boldsymbol{L}')^T\|_F^2$ for constant $m = \left(\|\boldsymbol{\Sigma}^*\|_2 - \sqrt{\|\boldsymbol{\Sigma}^*\|_2^2 - \|\boldsymbol{\Sigma}^*\|_2}\right)/2 > 0$ if $\|\boldsymbol{\Sigma}^*\|_2 \ge 1$ and $m = \|\boldsymbol{\Sigma}^*\|_2/2$ if $\|\boldsymbol{\Sigma}^*\|_2 < 1$. For the type I error probability, we have under the true model, $\boldsymbol{Y}_i - ((\boldsymbol{\mu}^*)(\boldsymbol{L}^*)^T)_i \sim N(0, \boldsymbol{\Sigma}^*)$ for

each i. Thus, by Hoeffding's inequality for sub-Gaussian random variables,

$$\begin{aligned}
&\mathbb{P}_*\left(\|\boldsymbol{Y}-(\boldsymbol{\mu}^*)(\boldsymbol{L}^*)^T\|_F^2-\|\boldsymbol{Y}-(\boldsymbol{\mu}')(\boldsymbol{L}')^T\|_F^2\geq\rho\right)\\
&\quad=\mathbb{P}_*\left(\sum_{i=1}^n(\boldsymbol{Y}_i-((\boldsymbol{\mu}^*)(\boldsymbol{L}^*)^T)_i)^T((\boldsymbol{\mu}')(\boldsymbol{L}')^T-(\boldsymbol{\mu}^*)(\boldsymbol{L}^*)^T)_i\geq m\|(\boldsymbol{\mu}^*)(\boldsymbol{L}^*)^T-(\boldsymbol{\mu}')(\boldsymbol{L}')^T\|_F^2\right)\\
&\quad\leq 2\exp\left(-\frac{c_1m^2\|(\boldsymbol{\mu}')(\boldsymbol{L}')^T-(\boldsymbol{\mu}^*)(\boldsymbol{L}^*)^T\|_F^4}{\|(\boldsymbol{\Sigma}^*)^{\frac{1}{2}}((\boldsymbol{\mu}')(\boldsymbol{L}')^T-(\boldsymbol{\mu}^*)(\boldsymbol{L}^*)^T)\|_F^2}\right)\\
&\quad\leq\exp\left(-c_1'\|(\boldsymbol{\mu}')(\boldsymbol{L}')^T-(\boldsymbol{\mu}^*)(\boldsymbol{L}^*)^T\|_F^2\right)
\end{aligned}$$

for some constants $c_1,c_1'>0$ since $\frac{m^2}{\|\boldsymbol{\Sigma}^*\|_2}\geq\frac{1}{\|\boldsymbol{\Sigma}^*\|_2}\geq\frac{1}{M_{\boldsymbol{\Sigma}}}>0$. For the type II error,

$$\begin{aligned}
C&\triangleq\int\frac{p^*(\boldsymbol{Y})p(\boldsymbol{Y})}{p_0(\boldsymbol{Y})}d\boldsymbol{Y}=\mathbb{E}_*\frac{p(\boldsymbol{Y})}{p_0(\boldsymbol{Y})}=\mathbb{E}_*\exp\left(-\frac{1}{2}(\|\boldsymbol{Y}-\boldsymbol{\mu}\boldsymbol{L}^T\|_F^2-\|\boldsymbol{Y}-(\boldsymbol{\mu}^*)(\boldsymbol{L}^*)^T\|_F^2)\right)\\
&=\mathbb{E}_*\exp\left(\sum_{i=1}^n(\boldsymbol{Y}_i-((\boldsymbol{\mu}^*)(\boldsymbol{L}^*)^T)_i)^T(\boldsymbol{\mu}\boldsymbol{L}^T-(\boldsymbol{\mu}^*)(\boldsymbol{L}^*)^T)_i-\frac{1}{2}\|\boldsymbol{\mu}\boldsymbol{L}^T-(\boldsymbol{\mu}^*)(\boldsymbol{L}^*)^T\|_F^2\right)\\
&=\exp\left(\frac{1}{2}\|(\boldsymbol{\Sigma}^*)^{\frac{1}{2}}(\boldsymbol{\mu}\boldsymbol{L}^T-(\boldsymbol{\mu}^*)(\boldsymbol{L}^*)^T)\|_F^2-\frac{1}{2}\|\boldsymbol{\mu}\boldsymbol{L}^T-(\boldsymbol{\mu}^*)(\boldsymbol{L}^*)^T\|_F^2\right)\\
&\leq\exp\left(\frac{1}{2}\left(\|\boldsymbol{\Sigma}^*\|_2-1\right)\|\boldsymbol{\mu}\boldsymbol{L}^T-(\boldsymbol{\mu}^*)(\boldsymbol{L}^*)^T\|_F^2\right)\\
&\leq\exp\left(\frac{1}{2}\left(\|\boldsymbol{\Sigma}^*\|_2-1\right)\left(\|\boldsymbol{\mu}\boldsymbol{L}^T-(\boldsymbol{\mu}')(\boldsymbol{L}')^T\|_F+\|(\boldsymbol{\mu}')(\boldsymbol{L}')^T-(\boldsymbol{\mu}^*)(\boldsymbol{L}^*)^T\|_F\right)^2\right)\\
&\leq\exp\left(\frac{1}{2}(1+\delta)^2\left(\|\boldsymbol{\Sigma}^*\|_2-1\right)\|(\boldsymbol{\mu}')(\boldsymbol{L}')^T-(\boldsymbol{\mu}^*)(\boldsymbol{L}^*)^T\|_F^2)\right)<\infty.
\end{aligned}$$

Therefore,

$$\mathbb{E}_{(\boldsymbol{\mu},\boldsymbol{L},\boldsymbol{I}_p)}\left(\frac{p^*(\boldsymbol{Y})}{p_0(\boldsymbol{Y})}(1-\phi_n)\right)=C\,\mathbb{P}_{\boldsymbol{Z}}\left(\|\boldsymbol{Z}-(\boldsymbol{\mu}^*)(\boldsymbol{L}^*)^T\|_F^2-\|\boldsymbol{Z}-(\boldsymbol{\mu}')(\boldsymbol{L}')^T\|_F^2<\rho\right)$$

where $\boldsymbol{Z}$ has density $\frac{p(\boldsymbol{Z})p^*(\boldsymbol{Z})}{Cp_0(\boldsymbol{Z})}$. We then have

$$\begin{aligned}
&\frac{p(\boldsymbol{Z})p^*(\boldsymbol{Z})}{p_0(\boldsymbol{Z})}\\
&\quad\propto\exp\left(-\frac{1}{2}\left(\|(\boldsymbol{\Sigma}^*)^{-\frac{1}{2}}(\boldsymbol{Z}-(\boldsymbol{\mu}^*)(\boldsymbol{L}^*)^T)\|_F^2+\|\boldsymbol{Z}-\boldsymbol{\mu}\boldsymbol{L}^T\|_F^2-\|\boldsymbol{Z}-(\boldsymbol{\mu}^*)(\boldsymbol{L}^*)^T\|_F^2\right)\right)\\
&\quad=\exp\Bigg(-\frac{1}{2}\Big(\|(\boldsymbol{\Sigma}^*)^{-\frac{1}{2}}(\boldsymbol{Z}-(\boldsymbol{\mu}^*)(\boldsymbol{L}^*)^T)\|_F^2+\|(\boldsymbol{\mu}^*)(\boldsymbol{L}^*)^T-\boldsymbol{\mu}\boldsymbol{L}^T\|_F^2\\
&\qquad\qquad+2\langle(\boldsymbol{\Sigma}^*)^{-\frac{1}{2}}(\boldsymbol{Z}-(\boldsymbol{\mu}^*)(\boldsymbol{L}^*)),(\boldsymbol{\Sigma}^*)^{\frac{1}{2}}((\boldsymbol{\mu}^*)(\boldsymbol{L}^*)^T-\boldsymbol{\mu}\boldsymbol{L}^T)\rangle_F\Big)\Bigg)\\
&\quad\propto\exp\left(-\frac{1}{2}\left(\|(\boldsymbol{\Sigma}^*)^{-\frac{1}{2}}\left(\boldsymbol{Z}-(\boldsymbol{\mu}^*)(\boldsymbol{L}^*)^T+\boldsymbol{\Sigma}^*((\boldsymbol{\mu}^*)(\boldsymbol{L}^*)^T-\boldsymbol{\mu}\boldsymbol{L}^T)\right)\|_F^2\right)\right).
\end{aligned}$$

Thus $\boldsymbol{Z}_i \sim N(((\boldsymbol{\mu}^*)(\boldsymbol{L}^*)^T)_i - \boldsymbol{\Sigma}^*((\boldsymbol{\mu}^*)(\boldsymbol{L}^*)^T - \boldsymbol{\mu}\boldsymbol{L}^T)_i, \boldsymbol{\Sigma}^*)$ and $\boldsymbol{Z}_i - (\boldsymbol{\mu}\boldsymbol{L}^T)_i \sim N((\boldsymbol{I}_p - \boldsymbol{\Sigma}^*)((\boldsymbol{\mu}^*)(\boldsymbol{L}^*)^T - \boldsymbol{\mu}\boldsymbol{L}^T)_i, \boldsymbol{\Sigma}^*)$ for $i \in [n]$. Then, by the same argument as type I error,

$$\begin{aligned}
&\mathbb{P}_{\boldsymbol{Z}}(\|\boldsymbol{Z} - (\boldsymbol{\mu}^*)(\boldsymbol{L}^*)^T\|_F^2 - \|\boldsymbol{Z} - (\boldsymbol{\mu}')(\boldsymbol{L}')^T\|_F^2 < \rho) \\
&\quad = \mathbb{P}_{\boldsymbol{Z}}\left(\sum_{i=1}^n (\boldsymbol{Z}_i - ((\boldsymbol{\mu}^*)(\boldsymbol{L}^*)^T)_i)^T((\boldsymbol{\mu}')(\boldsymbol{L}')^T - (\boldsymbol{\mu}^*)(\boldsymbol{L}^*)^T)_i < m\|(\boldsymbol{\mu}^*)(\boldsymbol{L}^*)^T - (\boldsymbol{\mu}')(\boldsymbol{L}')^T\|_F^2\right) \\
&\quad = \mathbb{P}_{\boldsymbol{Z}}\Bigg(\sum_{i=1}^n (\boldsymbol{Z}_i - (\boldsymbol{\mu}\boldsymbol{L}^T)_i)^T((\boldsymbol{\mu}')(\boldsymbol{L}')^T - (\boldsymbol{\mu}^*)(\boldsymbol{L}^*)^T)_i < m\|(\boldsymbol{\mu}^*)(\boldsymbol{L}^*)^T - (\boldsymbol{\mu}')(\boldsymbol{L}')^T\|_F^2 \\
&\qquad\qquad - \sum_{i=1}^n (\boldsymbol{\mu}\boldsymbol{L}^T - (\boldsymbol{\mu}^*)(\boldsymbol{L}^*)^T)_i^T((\boldsymbol{\mu}')(\boldsymbol{L}')^T - (\boldsymbol{\mu}^*)(\boldsymbol{L}^*)^T)_i\Bigg) \\
&\quad \leq \mathbb{P}_{\boldsymbol{Z}}\left(\sum_{i=1}^n (\boldsymbol{Z}_i - (\boldsymbol{\mu}\boldsymbol{L}^T)_i)^T((\boldsymbol{\mu}')(\boldsymbol{L}')^T - (\boldsymbol{\mu}^*)(\boldsymbol{L}^*)^T)_i < (\delta - 1 + m)\,\|(\boldsymbol{\mu}^*)(\boldsymbol{L}^*)^T - (\boldsymbol{\mu}')(\boldsymbol{L}')^T\|_F^2\right).
\end{aligned}$$

Note that the inequality comes from Cauchy-Schwarz inequality,

$$\begin{aligned}
&\langle \boldsymbol{\mu}\boldsymbol{L}^T - (\boldsymbol{\mu}^*)(\boldsymbol{L}^*)^T, (\boldsymbol{\mu}^*)(\boldsymbol{L}^*)^T - (\boldsymbol{\mu}')(\boldsymbol{L}')^T\rangle_F \\
&\quad = \langle \boldsymbol{\mu}\boldsymbol{L}^T - (\boldsymbol{\mu}')(\boldsymbol{L}')^T, (\boldsymbol{\mu}^*)(\boldsymbol{L}^*)^T - (\boldsymbol{\mu}')(\boldsymbol{L}')^T\rangle_F - \|(\boldsymbol{\mu}^*)(\boldsymbol{L}^*)^T - (\boldsymbol{\mu}')(\boldsymbol{L}')^T\|_F^2 \\
&\quad \leq (\delta - 1)\|(\boldsymbol{\mu}^*)(\boldsymbol{L}^*)^T - (\boldsymbol{\mu}')(\boldsymbol{L}')^T\|_F^2.
\end{aligned}$$

Denote $T = \sum_{i=1}^n (\boldsymbol{\mu}\boldsymbol{L}^T - (\boldsymbol{\mu}^*)(\boldsymbol{L}^*)^T)_i^T(\boldsymbol{\Sigma}^* - \boldsymbol{I}_p)((\boldsymbol{\mu}')(\boldsymbol{L}')^T - (\boldsymbol{\mu}^*)(\boldsymbol{L}^*)^T)_i$ as the mean of random variable $\sum_{i=1}^n (\boldsymbol{Z}_i - (\boldsymbol{\mu}\boldsymbol{L}^T)_i)^T((\boldsymbol{\mu}')(\boldsymbol{L}')^T - (\boldsymbol{\mu}^*)(\boldsymbol{L}^*)^T)_i$. Similarly we have

$$\begin{aligned}
&- \langle \boldsymbol{\mu}\boldsymbol{L}^T - (\boldsymbol{\mu}^*)(\boldsymbol{L}^*)^T, (\boldsymbol{\mu}^*)(\boldsymbol{L}^*)^T - (\boldsymbol{\mu}')(\boldsymbol{L}')^T\rangle_F \\
&\quad = \langle \boldsymbol{\mu}\boldsymbol{L}^T - (\boldsymbol{\mu}')(\boldsymbol{L}')^T, (\boldsymbol{\mu}')(\boldsymbol{L}')^T - (\boldsymbol{\mu}^*)(\boldsymbol{L}^*)^T\rangle_F + \|(\boldsymbol{\mu}^*)(\boldsymbol{L}^*)^T - (\boldsymbol{\mu}')(\boldsymbol{L}')^T\|_F^2 \\
&\quad \leq (\delta + 1)\|(\boldsymbol{\mu}^*)(\boldsymbol{L}^*)^T - (\boldsymbol{\mu}')(\boldsymbol{L}')^T\|_F^2.
\end{aligned}$$

Thus,

$$\begin{aligned}
-T &= \sum_i (\boldsymbol{\mu}\boldsymbol{L}^T - (\boldsymbol{\mu}^*)(\boldsymbol{L}^*)^T)_i^T(\boldsymbol{\Sigma}^* - \boldsymbol{I}_p)((\boldsymbol{\mu}^*)(\boldsymbol{L}^*)^T - (\boldsymbol{\mu}')(\boldsymbol{L}')^T)_i \\
&\leq \|\boldsymbol{\Sigma}^*\|_2\langle(\boldsymbol{\mu}\boldsymbol{L}^T - (\boldsymbol{\mu}^*)(\boldsymbol{L}^*)^T), (\boldsymbol{\mu}^*)(\boldsymbol{L}^*)^T - (\boldsymbol{\mu}')(\boldsymbol{L}')^T\rangle_F \\
&\qquad - \langle \boldsymbol{\mu}\boldsymbol{L}^T - (\boldsymbol{\mu}^*)(\boldsymbol{L}^*)^T, (\boldsymbol{\mu}^*)(\boldsymbol{L}^*)^T - (\boldsymbol{\mu}')(\boldsymbol{L}')^T\rangle_F \\
&\leq (\|\boldsymbol{\Sigma}^*\|_2(\delta - 1) + (\delta + 1))\,\|(\boldsymbol{\mu}^*)(\boldsymbol{L}^*)^T - (\boldsymbol{\mu}')(\boldsymbol{L}')^T\|_F^2.
\end{aligned}$$

Therefore, by Hoeffding's inequality,

$$\begin{aligned}
&\mathbb{P}_{\boldsymbol{Z}}(\|\boldsymbol{Z} - (\boldsymbol{\mu}^*)(\boldsymbol{L}^*)^T\|_F^2 - \|\boldsymbol{Z} - (\boldsymbol{\mu}')(\boldsymbol{L}')^T\|_F^2 < \rho) \\
&\quad \leq \mathbb{P}_{\boldsymbol{Z}}\Bigg(\sum_{i=1}^n (\boldsymbol{Z}_i - (\boldsymbol{\mu}\boldsymbol{L}^T)_i)^T((\boldsymbol{\mu}')(\boldsymbol{L}')^T - (\boldsymbol{\mu}^*)(\boldsymbol{L}^*)^T)_i - T \\
&\qquad\qquad < (\delta - 1 + m + \|\boldsymbol{\Sigma}^*\|_2(\delta - 1) + \delta + 1)\,\|(\boldsymbol{\mu}^*)(\boldsymbol{L}^*)^T - (\boldsymbol{\mu}')(\boldsymbol{L}')^T\|_F^2\Bigg) \\
&\quad \leq 2\exp\left(-\frac{c_2^2\|(\boldsymbol{\mu}')(\boldsymbol{L}')^T - (\boldsymbol{\mu}^*)(\boldsymbol{L}^*)^T\|_F^4}{2\|(\boldsymbol{\Sigma}^*)^{\frac{1}{2}}(\boldsymbol{\mu}')(\boldsymbol{L}')^T - (\boldsymbol{\mu}^*)(\boldsymbol{L}^*)^T\|_F^2}\right) \leq 2\exp\left(-c_2'\|(\boldsymbol{\mu}')(\boldsymbol{L}')^T - (\boldsymbol{\mu}^*)(\boldsymbol{L}^*)^T\|_F^2\right)
\end{aligned}$$

for constant $c_2' = c_2^2/(2\|\boldsymbol{\Sigma}^*\|_2) > 0$ where we use the fact that $c_2 = \delta - 1 + m + \|\boldsymbol{\Sigma}^*\|_2(\delta - 1) + \delta + 1 = (\|\boldsymbol{\Sigma}^*\|_2 + 2)\delta - (\|\boldsymbol{\Sigma}^*\|_2 - m) < 0$ since $\delta < \frac{\|\boldsymbol{\Sigma}^*\|_2}{2(\|\boldsymbol{\Sigma}^*\|_2+2)} < \frac{\|\boldsymbol{\Sigma}^*\|_2 - m}{\|\boldsymbol{\Sigma}^*\|_2+2}$. In addition, $\frac{c_2^2}{\|\boldsymbol{\Sigma}^*\|_2} \geq \frac{(\|\boldsymbol{\Sigma}^*\|_2 - m)^2}{\|\boldsymbol{\Sigma}^*\|_2} \geq \frac{\|\boldsymbol{\Sigma}^*\|_2}{4} \geq \frac{m_{\boldsymbol{\Sigma}}}{4} > 0$. As a result,

$$\begin{aligned}\mathbb{E}_{(\boldsymbol{\mu},\boldsymbol{L},\boldsymbol{I}_p)}\left(\frac{p^*(\boldsymbol{Y})}{p_0(\boldsymbol{Y})}(1-\phi_n)\right) &= C\,\mathbb{P}_{\boldsymbol{Z}}\left(\|\boldsymbol{Z} - (\boldsymbol{\mu}^*)(\boldsymbol{L}^*)^T\|_F^2 - \|\boldsymbol{Z} - (\boldsymbol{\mu}')(\boldsymbol{L}')^T\|_F^2 < \rho\right)\\ &\leq 2\exp\left(-c_3\|\boldsymbol{\mu}\boldsymbol{L}^T - (\boldsymbol{\mu}^*)(\boldsymbol{L}^*)^T\|_F^2\right)\end{aligned}$$

for some constant

$$\begin{aligned}c_3 &= c_2' - \frac{1}{2}(1+\delta)^2\left(\|\boldsymbol{\Sigma}^*\|_2 - 1\right)\\ &= \frac{((\|\boldsymbol{\Sigma}^*\|_2 + 2)\delta + m - \|\boldsymbol{\Sigma}^*\|_2)^2 - \|\boldsymbol{\Sigma}^*\|_2(\|\boldsymbol{\Sigma}^*\|_2 - 1)(1+\delta)^2}{2\|\boldsymbol{\Sigma}^*\|_2}\\ &= \frac{(5\|\boldsymbol{\Sigma}^*\|_2 + 4)\delta^2 + (-4\|\boldsymbol{\Sigma}^*\|_2^2 + 2\|\boldsymbol{\Sigma}^*\|_2 m + 4m - 2\|\boldsymbol{\Sigma}^*\|_2)\delta + (m^2 - 2\|\boldsymbol{\Sigma}^*\|_2 m + \|\boldsymbol{\Sigma}^*\|_2)}{2\|\boldsymbol{\Sigma}^*\|_2}.\end{aligned}$$

Denote $c_4(\delta) = 2\|\boldsymbol{\Sigma}^*\|_2 c_3$ as a quadratic function of δ, then $c_4(0) = m^2 - 2\|\boldsymbol{\Sigma}^*\|_2 m + \|\boldsymbol{\Sigma}^*\|_2 > 0$. When $\|\boldsymbol{\Sigma}^*\|_2 \geq 1$, $m = \frac{\|\boldsymbol{\Sigma}^*\|_2 - \sqrt{\|\boldsymbol{\Sigma}^*\|_2^2 - \|\boldsymbol{\Sigma}^*\|_2}}{2} \leq \frac{1}{2}$. When $\|\boldsymbol{\Sigma}^*\|_2 < 1$, $m = \frac{\|\boldsymbol{\Sigma}^*\|_2}{2} \leq \frac{1}{2}$. Thus $c_4(0) \geq \frac{1}{4}$ and therefore there exists $\delta > 0$ such that $c_3 \geq \frac{1}{8\|\boldsymbol{\Sigma}^*\|_2} \geq \frac{1}{8M_{\boldsymbol{\Sigma}}} > 0$. ■

Proof of Lemma 14 Denote $\mathcal{F}_{nK} = \{(\boldsymbol{\mu}, \boldsymbol{L}) : \boldsymbol{\mu} \in \mathbb{R}^{p\times K}, (\boldsymbol{\mu}_k)_{S_\delta} \in [-a_n, a_n]^{\beta(s + n\log K/\log p)}$ for $k \in [K], |\mathrm{supp}_\delta(\boldsymbol{\mu})| \leq \beta(s + n\log K/\log p), \boldsymbol{L} = \mathcal{L}_K, K \leq K_{\max}\}$. Then $N(\epsilon_n, \mathcal{F}_n, d) \leq \sum_{K=1}^{K_{\max}} N(\epsilon_n, \mathcal{F}_{nK}, d)$ since $\mathcal{F}_{nK_1}$ and $\mathcal{F}_{nK_2}$ are disjoint for $K_1 \neq K_2$. Consider for fixed $\boldsymbol{L}$,

$$\frac{\|\boldsymbol{\mu}\boldsymbol{L}^T - \boldsymbol{\mu}'\boldsymbol{L}^T\|_F^2}{n} \leq \frac{\|\boldsymbol{\mu} - \boldsymbol{\mu}'\|_F^2\|\boldsymbol{L}\|_F^2}{n} = \|\boldsymbol{\mu} - \boldsymbol{\mu}'\|_F^2.$$

Denote $\mathcal{G}_{nK1} \triangleq \{\boldsymbol{\mu} \in \mathbb{R}^{p\times K} : (\boldsymbol{\mu}_k)_{S_\delta} \in [-a_n, a_n]^{\beta(s+n\log K/\log p)}, k \in [K]\}$ and $\mathcal{G}_{nK2} \triangleq \mathcal{L}_K$. We know that the cardinality of $\mathcal{G}_{nK2}$ is K^n. Therefore, $\log N(\epsilon_n, \mathcal{F}_{nK}, d) \leq n\log K + \log N(\epsilon_1, \mathcal{G}_{nK1}, \|\cdot\|_F)$ where $\epsilon_1^2 = (s\log p + n\log K_{\max})/n$. Let $\mathcal{G}_{nK1\delta^c} \triangleq \{\boldsymbol{\mu} \in \mathcal{G}_{nK1} : \boldsymbol{\mu}_{S_\delta} = 0\}$ and $\mathcal{G}_{nK1\delta} \triangleq \{\boldsymbol{\mu} \in \mathcal{G}_{nK1} : \boldsymbol{\mu}_{S_\delta^c} = 0\}$. Suppose $\mathcal{N}_{\delta^c}$ and $\mathcal{N}_\delta$ are the minimal $\epsilon_1/2$-coverings of $\mathcal{G}_{nK1\delta^c}$ and $\mathcal{G}_{nK1\delta}$ respectively. Then for any $\boldsymbol{\mu} \in \mathcal{G}_{nK1}$, there exists $\tilde{\boldsymbol{\mu}} \in \mathcal{N}_{\delta^c}$ and $\bar{\boldsymbol{\mu}} \in \mathcal{N}_\delta$ such that $\|\boldsymbol{\mu} - (\tilde{\boldsymbol{\mu}} + \bar{\boldsymbol{\mu}})\|_F \leq \|\boldsymbol{\mu}_{S_\delta^c} - \tilde{\boldsymbol{\mu}}_{S_\delta^c}\|_F + \|\boldsymbol{\mu}_{S_\delta} - \bar{\boldsymbol{\mu}}_{S_\delta}\|_F \leq \epsilon_1$. Thus, we have $\log N(\epsilon_1, \mathcal{G}_{nK1}, \|\cdot\|_F) \leq \log N(\epsilon_1/2, \mathcal{G}_{nK1\delta^c}, \|\cdot\|_F) + \log N(\epsilon_1/2, \mathcal{G}_{nK1\delta}, \|\cdot\|_F)$.

Note that for $\mathcal{G}_{nK1\delta^c}$, we have $|\mathcal{G}_{nK1\delta^c}| < \delta^{pK}$. Since $\delta \lesssim \log p/(pK_{\max}\sqrt{n/\log p})$, we know that $|\mathcal{G}_{nK1\delta^c}| \lesssim |B_{\epsilon_1}^{p\times K}(0)|$ where $B_{\epsilon_1}^{p\times K}(0)$ is an ϵ_1-ball in $\mathbb{R}^{p\times K}$. Thus $N(\epsilon_1/2, \mathcal{G}_{nK1\delta^c}, \|\cdot\|_F)$ is bounded above by some constant. We know that for a subset of Euclidean space,

$$\log N(\epsilon_1, \mathcal{G}_{nK1\delta}, \|\cdot\|_F) \leq \beta\left(s + \frac{n\log K}{\log p}\right)K\log\frac{3a_n}{\epsilon_1} + \log\binom{p}{\beta\left(s + \frac{n\log K}{\log p}\right)}.$$

Note that since $(s + n\log K/\log p)/p \to 0$ as $n \to \infty$, by Stirling's formula we have $\log\binom{p}{\beta\left(s+\frac{n\log K}{\log p}\right)} \lesssim s\log p + n\log K$. By letting $a_n = (s\log p + n\log K_{\max})n^\gamma$ we have

$\log N(\epsilon_1, \mathcal{G}_{nK1}, \|\cdot\|_F) \lesssim s\log p + n\log K$ since $K\log n \lesssim \log p$. Thus, $\log N(\epsilon_n, \mathcal{F}_{nK}, d) \lesssim s\log p + n\log K$. Therefore, for some constant $c' > 0$,

$$N(\epsilon_n, \mathcal{F}_n, d) \le \sum_{K=1}^{K_{\max}} \exp(c(s\log p + n\log K)) \le \exp(c'(s\log p + n\log K_{\max})) = \exp(c' n\epsilon_n^2).$$

■

Proof of Lemma 15 We have

$$\begin{aligned}
\Pi(\mathcal{F}_n^c) &\le \sum_{k=1}^{K_{\max}} \Pi\left(\bigcup_{i=1}^{k}\{\|\boldsymbol{\mu}_i\|_\infty > a_n\}\,\middle|\, K=k\right)\Pi(K=k) \\
&\qquad + \Pi\left(|\mathrm{supp}_\delta(\boldsymbol{\mu})| > \beta\left(s + \frac{n\log K_{\max}}{\log p}\right)\right) \\
&\le \sum_{k=1}^{K_{\max}} kp\Pi(|\mu_{11}| > a_n)\Pi(K=k) + \Pi\left(|\mathrm{supp}_\delta(\boldsymbol{\mu})| > \beta\left(s + \frac{n\log K_{\max}}{\log p}\right)\right) \\
&\le p\Pi(|\mu_{11}| > a_n)\sum_{k=1}^{K_{\max}} k\Pi(K=k) + \Pi\left(|\mathrm{supp}_\delta(\boldsymbol{\mu})| > \beta\left(s + \frac{n\log K_{\max}}{\log p}\right)\right) \\
&\le \frac{\lambda}{1-e^{-\lambda}} p\Pi(|\mu_{11}| > a_n) + \Pi\left(|\mathrm{supp}_\delta(\boldsymbol{\mu})| > \beta\left(s + \frac{n\log K_{\max}}{\log p}\right)\right).
\end{aligned}$$

By lemma 12, the last term on the right hand side of the inequality is bounded above

$$\Pi\left(|\mathrm{supp}_\delta(\boldsymbol{\mu})| > \beta\left(s + \frac{n\log K_{\max}}{\log p}\right)\right) \le \exp\left(-c(s\log p + n\log K_{\max})\right)$$

for some constant $c > 0$. By the spike-and-slab lasso prior, we know that $|\mu_{11}| \sim (1-\theta)\mathrm{Exp}(\lambda_0) + \theta\mathrm{Exp}(\lambda_1)$. Let $X = |\mu_{11}|$, we have $X \mid \theta \sim (1-\theta)\mathrm{Exp}(\lambda_0) + \theta\mathrm{Exp}(\lambda_1)$. Then

$$\begin{aligned}
\sup_{m\ge1}\frac{(\mathbb{E}[|X|^m])^{1/m}}{m} &= \sup_{m\ge1}\frac{(\mathbb{E}[\mathbb{E}[|X|^m \mid \theta]])^{1/m}}{m} = \sup_{m\ge1}\frac{\mathbb{E}\left[(1-\theta)\frac{m!}{\lambda_0^m} + \theta\frac{m!}{\lambda_1^m}\right]^{1/m}}{m} \\
&= \sup_{m\ge1}\frac{1}{m}\left\{\left(1 - \frac{1}{1+\beta_\theta}\right)\frac{m!}{\lambda_0^m} + \frac{1}{1+\beta_\theta}\frac{m!}{\lambda_1^m}\right\}^{1/m} \\
&\le \sup_{m\ge1}\frac{1}{m}\frac{(m!)^{1/m}}{\lambda_1} \le \sup_{m\ge1}\frac{1}{m}\frac{(em)^{1/m}m}{e\lambda_1} \le \frac{2}{\lambda_1} \le 2n^\gamma \le \infty
\end{aligned}$$

for any $n > 0$. Note that the first inequality is due to the power mean inequality for $\lambda_0 \ge \lambda_1$. Thus by Bernstein inequality, we have

$$\begin{aligned}
\Pi(X \ge a_n) &= \Pi(X - \mathbb{E}X \ge a_n - \mathbb{E}X) \le \Pi(X - \mathbb{E}X \ge \frac{a_n}{2}) \\
&\le 2\exp\left(-c\min\left(\frac{a_n^2}{4\|X\|_{\psi_1}}, \frac{a_n}{2\|X\|_{\psi_1}}\right)\right) \le \exp(-c(s\log p + n\log K_{\max})).
\end{aligned}$$

Note that here $\mathbb{E}\, X = \frac{1-\theta}{\lambda_0} + \frac{\theta}{\lambda_1} \leq \frac{a_n}{2}$ and $a_n = (s \log p + n \log K_{\max}) n^{\gamma}$.

■

B.3 Proof of Theorem 4

Proof of Theorem 4 Denote $\bar{\epsilon}_n^2 = (s \log p + n \log K^*)/n$ and $\Theta = \bigcup_{K=1}^{K_{\max}} \mathbb{R}^{p \times K} \times \mathcal{L}_K$. Let d be a metric on Θ with $d((\boldsymbol{\mu}, \boldsymbol{L}), (\boldsymbol{\mu}', \boldsymbol{L}')) = \|\boldsymbol{\mu}\boldsymbol{L}^T - \boldsymbol{\mu}'\boldsymbol{L}'^T\|_F$. Let

$$U_n = \left\{(\boldsymbol{\mu}, \boldsymbol{L}) \in \Theta : d((\boldsymbol{\mu}, \boldsymbol{L}), (\boldsymbol{\mu}^*, \boldsymbol{L}^*)) < M\sqrt{n}\epsilon_n\right\}.$$

By Bayes rule, we have $\Pi(U_n^c \mid \boldsymbol{Y}) = \frac{\int_{U_n^c} \frac{p_n(\boldsymbol{Y})}{p_0(\boldsymbol{Y})} d\Pi}{\int \frac{p_n(\boldsymbol{Y})}{p_0(\boldsymbol{Y})} d\Pi} := \frac{N_n}{D_n}$ where

$$p_0(\boldsymbol{Y}) = (2\pi)^{-\frac{np}{2}} \exp\left(-\frac{1}{2}\|\boldsymbol{Y} - (\boldsymbol{\mu}^*)(\boldsymbol{L}^*)^T\|_F^2\right), p_n(\boldsymbol{Y}) = (2\pi)^{-\frac{np}{2}} \exp\left(-\frac{1}{2}\|\boldsymbol{Y} - \boldsymbol{\mu}\boldsymbol{L}^T\|_F^2\right).$$

We also denote $p^*(\boldsymbol{Y}) = (2\pi)^{-\frac{np}{2}} \det(\boldsymbol{\Sigma}^*)^{-\frac{n}{2}} \exp\left(-\frac{1}{2}\|(\boldsymbol{\Sigma}^*)^{-\frac{1}{2}}\left(\boldsymbol{Y} - (\boldsymbol{\mu}^*)(\boldsymbol{L}^*)^T\right)\|_F^2\right)$. By Lemma 11 we know that $\Pi\left(\|\boldsymbol{\mu}\boldsymbol{L}^T - (\boldsymbol{\mu}^*)(\boldsymbol{L}^*)^T\|_F^2 \leq s \log p\right) \geq \exp\left(-c_1 n \bar{\epsilon}_n^2\right)$ for some constant $c_1 > 0$. Denote $A_n = \{D_n > \exp(-c_2 n \bar{\epsilon}_n^2)\}$ for some $c_2 > c_1 > 0$. Thus,

$$A_n \supset \{D_n > \Pi(\|\boldsymbol{\mu}\boldsymbol{L}^T - (\boldsymbol{\mu}^*)(\boldsymbol{L}^*)^T\|_F^2 \leq s \log p) \exp(-(c_2 - c_1) n \bar{\epsilon}_n^2)\}.$$

Let $\mathbb{1}(A)$ denote the indicator random variable of event A. Then we can write

$$\begin{aligned}
&\mathbb{E}_* \Pi(U_n^c \mid Y) = \mathbb{E}_*\{(\phi_n + 1 - \phi_n)\Pi(U_n^c \mid \boldsymbol{Y})\} \\
&\quad = \mathbb{E}_*\{\phi_n \Pi(U_n^c \mid \boldsymbol{Y})\} + \mathbb{E}_*\{(1 - \phi_n)\mathbb{1}(A_n)\Pi(U_n^c \mid \boldsymbol{Y})\} + \mathbb{E}_*\{(1 - \phi_n)\mathbb{1}(A_n^c)\Pi(U_n^c \mid \boldsymbol{Y})\} \\
&\quad \leq \mathbb{E}_* \phi_n + \mathbb{E}_*\left\{(1 - \phi_n) \exp(c_2 n \bar{\epsilon}_n^2) \int_{U_n^c} \frac{p_n(\boldsymbol{Y})}{p_0(\boldsymbol{Y})} d\Pi\right\} + \mathbb{P}_*(A_n^c).
\end{aligned}$$

We treat the three terms on the right-hand side of the last equality separately. Denote

$$\mathcal{F}_n = \bigcup_{K=1}^{K_{\max}} \left\{(\boldsymbol{\mu}, \boldsymbol{L}) : |\mathrm{supp}_{\delta_{\boldsymbol{\mu}}}(\boldsymbol{\mu})| \leq \beta\left(s + \frac{n \log K}{\log p}\right), \max_{k \in [K]} \|(\boldsymbol{\mu}_k)_{S_\delta}\|_\infty \leq a_n, \boldsymbol{L} \in \mathcal{L}_K\right\}$$

for $\delta_{\boldsymbol{\mu}}$ and β defined in Lemma 12, and $a_n = (s \log p + n \log K_{\max}) n^{\gamma}$. Let $U_{n,j} = \{(\boldsymbol{\mu}, \boldsymbol{L}) \in \mathcal{F}_n : d((\boldsymbol{\mu}, \boldsymbol{L}), (\boldsymbol{\mu}^*, \boldsymbol{L}^*)) \in \left[j^2 n \epsilon_n^2, (j+1)^2 n \epsilon_n^2\right)\}$. Let $N_{n,j}$ be the maximal ϵ_n-nets $U_{n,j,1}, \ldots, U_{n,j,N_j}$ that covers $U_{n,j}$ with respect to metric d. By Lemma 13 we have that for each $U_{n,j,h} \in N_{n,j}$, there exists a test $\phi_{n,j,h}$ such that $\mathbb{E}_* \phi_{n,j,h} \leq \exp\left(-c_3 n j^2 \epsilon_n^2\right)$. Denote $\phi_n = \max_{j=M}^{\infty} \max_{h=1}^{|N_{n,j}|} \phi_{n,j,h}$. Then $\mathbb{E}_* \phi_n \leq \sum_{j=M}^{\infty} \sum_{h=1}^{|N_{n,j}|} \mathbb{E}_* \phi_{n,j,h}$. By Lemma 13, we have $\mathbb{E}_* \phi_n \leq N(\epsilon_n, \mathcal{F}_n, d) \frac{\exp(-c_3 n M^2 \epsilon_n^2)}{1 - \exp(-c_3 n \epsilon_n^2)}$. By lemma 14 we have $N(\epsilon_n, \mathcal{F}_n, d) \leq \exp(c_4 n \epsilon_n^2)$. Thus the first term goes to 0 under $\mathbb{E}_*$ as n tends to infinity and sufficiently large $M > 0$.

For the third term, by definition we have $\mathbb{P}_*(A_n^c) \leq 1 - \Pi\{D_n > \Pi(\|\boldsymbol{\mu}\boldsymbol{L}^T - (\boldsymbol{\mu}^*)(\boldsymbol{L}^*)^T\|_F^2 \leq s \log p) \exp(-(c_2 - c_1) n \bar{\epsilon}_n^2)\}$. Consider the event in the probability on the right hand side. By dividing both sides of the inequality by $\Pi(\|\boldsymbol{\mu}\boldsymbol{L}^T - (\boldsymbol{\mu}^*)(\boldsymbol{L}^*)^T\|_F^2 \leq s \log p)$, we can rewrite

it in terms of Π', which is the restricted and renormalized probability measure of prior Π conditioning on the event $E_n = \{\|\boldsymbol{\mu}\boldsymbol{L}^T - (\boldsymbol{\mu}^*)(\boldsymbol{L}^*)^T\|_F^2 \leq s\log p\}$. By Jensen's inequality,

$$\sum_i Z_{ni} \triangleq \int \sum_i \log \frac{p_n(\boldsymbol{Y}_i)}{p_0(\boldsymbol{Y}_i)} d\Pi' \leq \log \int \prod_i \frac{p_n(\boldsymbol{Y}_i)}{p_0(\boldsymbol{Y}_i)} d\Pi'.$$

Then we have that $\sum_i \mathbb{E}_* Z_{ni} = -\frac{1}{2}\int \|\boldsymbol{\mu}\boldsymbol{L}^T - (\boldsymbol{\mu}^*)(\boldsymbol{L}^*)^T\|_F^2 d\Pi' \geq -\frac{1}{2}s\log p$. So for the event $\{D_n' > \exp(-(c_2-c_1)n\bar{\epsilon}_n^2)\}$ where $D_n' = \int \prod_i \frac{p_n(\boldsymbol{Y}_i)}{p_0(\boldsymbol{Y}_i)} d\Pi'$, we have $\Pi(\log D_n' \geq -(c_2 - c_1)n\bar{\epsilon}_n^2) \geq \Pi\left(\sum_i Z_{ni} \geq -(c_2-c_1)n\bar{\epsilon}_n^2\right) \geq \Pi\left(\sum_i (Z_{ni} - \mathbb{E}_* Z_{ni}) \geq -c_2' n\bar{\epsilon}_n^2\right)$. Therefore, by Hoeffding inequality for sub-Gaussian random variable,

$$\Pi\left(\left|\sum_i Z_{ni} - \mathbb{E}_* Z_{ni}\right| \geq \frac{c_2' n\bar{\epsilon}_n^2}{2}\right) \leq 2\exp\left(-c_5 \frac{n^2\bar{\epsilon}_n^4}{\|(\boldsymbol{\Sigma}^*)^{\frac{1}{2}}((\boldsymbol{\mu}^*)(\boldsymbol{L}^*)^T - \boldsymbol{\mu}\boldsymbol{L}^T)\|_F^2}\right)$$
$$\leq 2\exp\left(-c_5 \frac{n^2\bar{\epsilon}_n^4}{\|(\boldsymbol{\Sigma}^*)^{\frac{1}{2}}\|_2^2 \|((\boldsymbol{\mu}^*)(\boldsymbol{L}^*)^T - \boldsymbol{\mu}\boldsymbol{L}^T)\|_F^2}\right) \leq \exp\left(-c_5' n\bar{\epsilon}_n^2\right)$$

for some constants $c_5, c_5' > 0$ since $\|\boldsymbol{\Sigma}^*\|_2$ is upper bounded from infinity. Thus, as $n \to \infty$, $\mathbb{E}_* \mathbb{1}(A_n^c) \leq 1 - \Pi(\log D_n' \geq -(c_2-c_1)n\bar{\epsilon}_n^2) \leq \exp(-c_5' n\bar{\epsilon}_n^2) \to 0$.

For the second term, we have, by Fubini's theorem,

$$\mathbb{E}_*(1-\phi_n)\exp(c_2 n\bar{\epsilon}_n^2)\int_{U_n^c} \frac{p_n(\boldsymbol{Y})}{p_0(\boldsymbol{Y})} d\Pi \leq \exp(c_2 n\bar{\epsilon}_n^2)\left(\int_{U_n^c \cap \mathcal{F}_n} \mathbb{E}_n\left((1-\phi_n)\frac{p^*(\boldsymbol{Y})}{p_0(\boldsymbol{Y})}\right) d\Pi + \Pi(\mathcal{F}_n^c)\right).$$

By Lemma 15, we know that $\log \Pi\left(\mathcal{F}_n^c\right) \lesssim -n\bar{\epsilon}_n^2$. By Lemma 13, we have $\mathbb{E}_n\left((1-\phi_n)\frac{p^*(\boldsymbol{Y})}{p_0(\boldsymbol{Y})}\right) \leq \exp(-c_5 n M^2 \epsilon_n^2)$ so the above term goes to 0 as n tends to infinity.

■

B.4 Proof of Theorem 8

Proof of Theorem 8 We find the singular value decomposition (SVD) of $\boldsymbol{\mu}\boldsymbol{L}^T = \boldsymbol{U}\Sigma\boldsymbol{V}^T$ for some diagonal matrix $\Sigma \in \mathbb{R}^{p\times n}$ and $\boldsymbol{U} \in \mathbb{O}^p$ and $\boldsymbol{V} \in \mathbb{O}^n$ where $\mathbb{O}^m$ denotes the set of m by m orthogonal matrices. Consider the matrix $\boldsymbol{L}$, let $\Sigma_L = \boldsymbol{L}^T\boldsymbol{L}$ be the diagonal matrix whose kth diagonal entry is the size of cluster k. Then denote $\boldsymbol{L}_N = \boldsymbol{L}\Sigma_L^{-1/2}$ as the "normalization" of $\boldsymbol{L}$ since it is orthogonal. On the other hand, for matrix $\boldsymbol{\mu}$, we suppose the corresponding QR decomposition is $\boldsymbol{\mu} = \boldsymbol{Q}\boldsymbol{R}$ for some $\boldsymbol{Q} \in \mathbb{O}^p$ and upper triangular matrix $\boldsymbol{R} \in \mathbb{R}^{p\times K}$. Then suppose the SVD of $\boldsymbol{R}\Sigma_L^{1/2}$ is $\boldsymbol{R}\Sigma_L^{1/2} = \boldsymbol{U}_1\Sigma_1\boldsymbol{V}_1^T$ for some $\boldsymbol{U}_1 \in \mathbb{O}^p$ and $\boldsymbol{V}_1 \in \mathbb{O}^K$. Therefore we obtain $\boldsymbol{\mu}\boldsymbol{L}^T = \boldsymbol{Q}\boldsymbol{U}_1\Sigma_1\boldsymbol{V}_1^T\boldsymbol{L}_N^T$ and we know that v_i, which denotes the ith column of $\boldsymbol{L}_N\boldsymbol{V}_1$, satisfies $\boldsymbol{\mu}\boldsymbol{L}^T v_i = \sigma_i u_i$ for $i = 1, \cdots, K$ where u_i is the ith column of $\boldsymbol{Q}\boldsymbol{U}_1$ and σ_i is the ith singular value of $\boldsymbol{\mu}\boldsymbol{L}^T$.

Then we can use a variant of Davis-Kahan theorem (Yu et al., 2014). Suppose $\boldsymbol{\mu}\boldsymbol{L}^T = \boldsymbol{Q}\boldsymbol{U}_1\Sigma_1\boldsymbol{V}_1^T\boldsymbol{L}_N^T$ and $(\boldsymbol{\mu}^*)(\boldsymbol{L}^*)^T = \boldsymbol{Q}^*\boldsymbol{U}_1^*\Sigma_1^*(\boldsymbol{V}_1^*)^T(\boldsymbol{L}_N^*)^T$. Denote $D_F(\boldsymbol{O}_1, \boldsymbol{O}_2) = \inf_{\boldsymbol{V}\in\mathbb{O}^r} \|\boldsymbol{O}_1 - \boldsymbol{O}_2\boldsymbol{V}\|_F$ for $\boldsymbol{O}_1, \boldsymbol{O}_2 \in \mathbb{O}^r$ and let $\|\sin\Theta(\boldsymbol{O}_1, \boldsymbol{O}_2)\|_F$ be the (Frobenius) sine-theta distance

between span($\boldsymbol{O}_1$) and span($\boldsymbol{O}_2$). Then the relationship between the metric D_F and sine-theta distance holds: $\|\sin\Theta(\boldsymbol{O}_1,\boldsymbol{O}_2)\|_F \le D_F(\boldsymbol{O}_1,\boldsymbol{O}_2) \le \sqrt{2}\|\sin\Theta(\boldsymbol{O}_1,\boldsymbol{O}_2)\|_F$. Note that for the right singular subspace of $\boldsymbol{\mu}\boldsymbol{L}^T$, we have

$$\begin{aligned}\sqrt{2}\|\sin\Theta(\boldsymbol{L}_N\boldsymbol{V}_1,\boldsymbol{L}_N^*\boldsymbol{V}_1^*)\|_F &\ge \inf_{\boldsymbol{V}\in\mathbb{O}^K}\|\boldsymbol{L}_N\boldsymbol{V}_1-\boldsymbol{L}_N^*\boldsymbol{V}_1^*\boldsymbol{V}\|_F\\ &= \inf_{\boldsymbol{V}\in\mathbb{O}^K}\|\left(\boldsymbol{L}_N-\boldsymbol{L}_N^*\boldsymbol{V}_1^*\boldsymbol{V}(\boldsymbol{V}_1)^{-1}\right)\boldsymbol{V}_1\|_F\\ &= \inf_{\boldsymbol{V}\in\mathbb{O}^K}\|\boldsymbol{L}_N-\boldsymbol{L}_N^*\boldsymbol{V}_1^*\boldsymbol{V}(\boldsymbol{V}_1^*)^{-1}\|_F \ge \|\sin\Theta(\boldsymbol{L}_N,\boldsymbol{L}_N^*)\|_F.\end{aligned}$$

Then by Theorem 3 in Yu et al. (2014), we have

$$\|\sin\Theta(\boldsymbol{L}_N,\boldsymbol{L}_N^*)\|_F \le \frac{2\sqrt{2}(2\sigma_{\max}+\|\boldsymbol{\mu}\boldsymbol{L}^T-(\boldsymbol{\mu}^*)(\boldsymbol{L}^*)^T\|_2)}{\sigma_{\min}^2}\times\|\boldsymbol{\mu}\boldsymbol{L}^T-(\boldsymbol{\mu}^*)(\boldsymbol{L}^*)^T\|_F$$

where $\sigma_{\max}$ and $\sigma_{\min}$ represent the max and min singular values of $(\boldsymbol{\mu}^*)(\boldsymbol{L}^*)^T$ respectively.

We denote $\boldsymbol{L}_N = [(l_N)_1,\ldots,(l_N)_n]^T$ and $\boldsymbol{L}_N^* = [(l_N)_1^*,\ldots,(l_N)_n^*]^T$. Note that $\boldsymbol{L}_N^*$ and $\boldsymbol{L}_N$ have at most K distinct rows. Let ζ be the minimum distance among these K distinct rows of $\boldsymbol{L}_N^*$ with respect to ℓ-2 norm: $\zeta = \min_{(l_N)_i^*\neq(l_N)_j^*}\|(l_N)_i^*-(l_N)_j^*\|_2$. Let $\boldsymbol{O} = \arg\inf_{\boldsymbol{V}\in\mathbb{O}^K}\|\boldsymbol{L}_N-\boldsymbol{L}_N^*\boldsymbol{V}\|_F$. Define the set $\mathcal{I} = \left\{i : \|(l_N)_i-\boldsymbol{O}^T(l_N)_i^*\|_2 \ge \zeta/2\right\}$. Assume that the event $\mathcal{E}_n = \{\|\sin\Theta(\boldsymbol{L}_N,\boldsymbol{L}_N^*)\|_F \le \eta_n\}$ occurs *a posteriori*, where $\eta_n = \frac{8\sqrt{2}\sigma_{\max}}{\sigma_{\min}^2}(M(s\log p+n\log K_{\max}))^{1/2}$ and M is the constant in Theorem 4. By Theorem 4, we know that $\mathbb{E}_*\{\Pi(\mathcal{E}_n)\}\to 1$ as $n\to\infty$. This implies that $|\mathcal{I}| \le 4\eta_n^2/\zeta^2$ since otherwise we have $\|\boldsymbol{L}_N-\boldsymbol{L}_N^*\boldsymbol{O}\|_F^2 \ge (\zeta^2/4)(4\eta_n^2/\zeta^2) = \eta_n^2$, which contradicts with the definition of $\mathcal{E}_n$. Thus, for any $i,j\in\mathcal{I}^c$ with $(l_N)_i = (l_N)_j$, $\|(l_N)_i^*-(l_N)_j^*\|_2 \le \|(l_N)_i-\boldsymbol{O}^T(l_N)_i^*\|_2+\|(\boldsymbol{l}_N)_j-\boldsymbol{O}^T(l_N)_j^*\|_2 < \zeta$, which implies $(l_N)_i^* = (l_N)_j^*$ since ζ is the minimum distance between pair of distinct rows of $\boldsymbol{L}_N^*$. On the other hand, note that $\zeta^2 = 1/n_{\max}^*+1/n_2$ where n_2 is the second largest cluster size. Consequently, since

$$\begin{aligned}\sigma_{\min}((\boldsymbol{\mu}^*)(\boldsymbol{L}^*)^T) &= \sqrt{\lambda_{K^*}((\boldsymbol{\mu}^*)(\boldsymbol{L}^*)^T(\boldsymbol{L}^*)(\boldsymbol{\mu}^*)^T)} \ge \sqrt{n_{\min}^*\lambda_{K^*}((\boldsymbol{\mu}^*)(\boldsymbol{\mu}^*)^T)} \ge n_{\min}^*\sigma_{\min}(\boldsymbol{\mu}^*),\\ \sigma_{\max}((\boldsymbol{\mu}^*)(\boldsymbol{L}^*)^T) &= \sqrt{\lambda_{\max}((\boldsymbol{\mu}^*)(\boldsymbol{L}^*)^T(\boldsymbol{L}^*)(\boldsymbol{\mu}^*)^T)} \le \sqrt{n_{\max}^*\lambda_{\max}((\boldsymbol{\mu}^*)(\boldsymbol{\mu}^*)^T)} \le n_{\max}^*\sigma_{\max}(\boldsymbol{\mu}^*),\end{aligned}$$

we have $|\mathcal{I}| \le \frac{4\eta_n^2}{\zeta^2} \asymp \frac{(n_{\max}^*)^3\sigma_{\max}(\boldsymbol{\mu}^*)^2}{(n_{\min}^*)^4\sigma_{\min}(\boldsymbol{\mu}^*)^4}(s\log p+n\log K_{\max})$. Note that $n_k^* > |\mathcal{I}|$ for all k, namely, $\{(l_N)_i^* : i\notin\mathcal{I}\}$ consists of all K^* distinct rows of $\boldsymbol{L}_N^*$. Therefore, each of the unique $B_{\|\cdot\|_2}(\boldsymbol{O}^T(l_N)_i^*,\zeta/2)$ for $i\in[n]$, which is the ℓ-2 ball centered at $\boldsymbol{O}^T(l_N)_i^*$ with radius $\zeta/2$, contains at least one element of $\{(l_N)_i : i\in\mathcal{I}^c\}$. Recall that ζ is the minimum distance between any pair of distinct rows of $\boldsymbol{L}_N^*$, so these open balls are disjoint for distinct rows of $\boldsymbol{L}_N^*$. It follows from the pigeonhole principle that each ball contains exactly one element of $\{(l_N)_i : i\in\mathcal{I}^c\}$. Thus, if $(l_N)_i^* = (l_N)_j^*$ for $i,j\in\mathcal{I}^c$, then $(l_N)_i,(l_N)_j\in B_{\|\cdot\|_2}(\boldsymbol{O}^T(l_N)_i^*,\zeta/2)$, implying that $(l_N)_i = (l_N)_j$ by the fact that every such ball contains exactly one row of $\boldsymbol{L}$.

Therefore we prove that for any $i\in\mathcal{I}^c$, $(l_N)_i = (l_N)_j$ if and only if $(l_N)_i^* = (l_N)_j^*$. So the number of mis-clustered points are at most $|\mathcal{I}|$, which gives us the result because $\mathbb{E}_*\{\Pi(\mathcal{E}_n)\}\to 1$ as $n\to\infty$ by Theorem 4. ■

Appendix C. Posterior Inference via Gibbs Sampling

In this section, we introduce a Gibbs sampler for posterior inference of the proposed Bayesian sparse Gaussian mixture model. We design a sampler based on the algorithm proposed in Miller and Harrison (2018). Let $\mathcal{C}$ denote the partition of $[n]$ according to the cluster memberships $\boldsymbol{z}$. Formally, $\mathcal{C} = \{E_k : |E_k| > 0\}$ where $E_k = \{i : z_i = k \text{ for } i \in [n]\}$. Let $\mathcal{C}_{-i}$ be the partition of $[n] \setminus \{i\}$ according to the cluster memberships $\{z_j\}_{j \neq i}$. We also denote n_c as the number of data points in $c \in \mathcal{C}$ and n_c^- as the number of data points in $c \in \mathcal{C}_{-i}$. We can derive an urn representation for the mixture model from the exchangeable partition distribution: $\pi(\mathcal{C}) = V_n(|\mathcal{C}|) \prod_{c \in \mathcal{C}} \alpha^{(|c|)}$, where $V_n(|\mathcal{C}|) = \sum_{k=1}^{\infty} p_K(k) k_{(|\mathcal{C}|)} / (\alpha k)^{(n)}$ and p_K is the prior of K. Here $x^{(m)} \triangleq x(x+1)\cdots(x+m-1)$, $x_{(m)} \triangleq x(x-1)\cdots(x-m+1)$.

To address the non-conjugacy of the Laplace distribution, we re-write the SSL(λ_0, λ_1, θ) prior through the normal-scale-mixture representation of Laplace as follows: for $j \in [p]$,

$$(x_j \mid \phi_j, \xi_j = a) \sim N\left(0, \frac{\phi_j}{\lambda_a^2}\right), a = 0, 1, \quad \phi_j \sim \text{Exp}\left(\frac{1}{2}\right), \quad \xi_j \sim \text{Bernoulli}(\theta).$$

We obtain the following closed-form full conditional posterior distributions of $\boldsymbol{\mu}_c$, $\boldsymbol{\phi}_c$ and ξ:

$$\begin{aligned}
((\boldsymbol{\mu}_c)_j \mid -) &\sim N\left(\sum_{l \in c} (\boldsymbol{Y}_l)_j \left(n_c + \frac{\lambda_{\xi_j}^2}{(\phi_c)_j}\right)^{-1}, \left(n_c + \frac{\lambda_{\xi_j}^2}{(\phi_c)_j}\right)^{-1}\right), \\
((\phi_c)_j \mid -) &\sim \text{GiG}(0.5, (\boldsymbol{\mu}_c)_j^2 \lambda_{\xi_j}^2, 1), \\
(\xi_j \mid -) &\sim \text{Bernoulli}(\theta'), \quad \text{where} \quad \theta' \propto \prod_{c \in \mathcal{C}} \frac{\lambda_1}{\sqrt{(\phi_c)_j}} \exp\left(-\frac{1}{2} \lambda_1^2 \frac{(\boldsymbol{\mu}_c)_j^2}{(\phi_c)_j}\right) \theta, \\
(\theta \mid -) &\sim \text{Beta}\left(1 + \sum_{j=1}^{p} \xi_j, \beta_\theta + p - \sum_{j=1}^{p} \xi_j\right).
\end{aligned}$$

Here, $\text{GiG}(\zeta, \chi, \tau)$ denotes the generalized inverse Gaussian distribution whose probability density function is $f(x) = x^{\zeta - 1} \exp\left(-\left(\chi / x + \tau x\right)/2\right)$. We also remark that there exists a potential label switching phenomenon when sampling centers $\boldsymbol{\mu}_c$ and auxiliary variables $\boldsymbol{\phi}_c$ for all clusters. This can be prevented by the following alignment process.

(i) Collect B post-burn-in samples $\boldsymbol{\mu}^{(b)} = \begin{pmatrix} \boldsymbol{\mu}_1^{(b)} & \cdots & \boldsymbol{\mu}_{K^{(b)}}^{(b)} \end{pmatrix}$ and $\boldsymbol{L}^{(b)} = \begin{pmatrix} \boldsymbol{l}_1^{(b)} & \cdots & \boldsymbol{l}_{K^{(b)}}^{(b)} \end{pmatrix}^T$ for $b = 1, \ldots, B$, where $K^{(b)} \triangleq |\mathcal{C}^{(b)}|$ is the number of clusters in bth iteration.

(ii) Find the index b^* that corresponds to the maximizer of the log-likelihood function: $b^* = \arg\min_{b \in [B]} \left\| \boldsymbol{Y} - (\boldsymbol{\mu}^{(b)})(\boldsymbol{L}^{(b)})^T \right\|_{\text{F}}^2$.

(iii) For $b = 1, \ldots, B$, find $\mathcal{P}^{(b)} = \arg\min_{\mathcal{P} \in \mathcal{S}^{K^{(b)} \times K^{(b)}}} \left\| \boldsymbol{\mu}^{(b^*)} - \boldsymbol{\mu}^{(b)} \mathcal{P} \right\|_{\text{F}}^2$, where $\mathcal{S}^{K^{(b)} \times K^{(b)}}$ is the set of all $K^{(b)} \times K^{(b)}$ permutation matrices.

(iv) For $b = 1, \ldots, B$, replace $\boldsymbol{\mu}^{(b)}$ by $\boldsymbol{\mu}^{(b)} \mathcal{P}^{(b)}$ and $\boldsymbol{L}^{(b)}$ by $\boldsymbol{L}^{(b)} \mathcal{P}^{(b)}$.

We provide the detailed Gibbs sampler in Algorithm 1 below. The R code can be found at `https://github.com/YanxunXu/HighDimClustering`.

Algorithm 1 The Gibbs sampler

Require: Initialization of $\mathcal{C}$,$\{\boldsymbol{\mu}_c : c \in \mathcal{C}\}$, $\boldsymbol{\xi}$, $\{\boldsymbol{\phi}_c : c \in \mathcal{C}\}$

for $b \leftarrow 1$ to B **do**
  for $i \leftarrow 1$ to n **do**
    $t \leftarrow |\mathcal{C}_{-i}|$
    if $z_i \neq z_l$ for all $l \neq i$ **then**
      Remove $\boldsymbol{\mu}_{z_j}$
    end if
    Sample $\boldsymbol{\phi}_{t+1} \sim p_{\boldsymbol{\phi}}(\boldsymbol{\phi}_{t+1})$
    Sample $\boldsymbol{\mu}_{t+1} \sim p_{\boldsymbol{\mu}|\boldsymbol{\xi},\boldsymbol{\phi}}(\boldsymbol{\mu}_{t+1} \mid \boldsymbol{\xi}, \boldsymbol{\phi}_{t+1})$
    for $k \leftarrow 1$ to t **do**
      $m_k \leftarrow (n_k^- + \alpha)p(\boldsymbol{Y}_i \mid \boldsymbol{\mu}_c)$ where n_k^- is the size of cluster k in $\mathcal{C}_{-j}$
    end for
    $V_n(t) \leftarrow \frac{t!}{n!}\frac{\Gamma(\alpha t)}{n^{\alpha t-1}}p_K(t)$
    $V_n(t+1) \leftarrow \frac{(t+1)!}{n!}\frac{\Gamma(\alpha(t+1))}{n^{\alpha(t+1)-1}}p_K(t+1)$
    $m_{t+1} \leftarrow \alpha\frac{V_n(t+1)}{V_n(t)}p(\boldsymbol{Y}_i \mid \boldsymbol{\mu}_{t+1})$
    Sample $z_i \sim \text{Categorical}\left(\frac{m_1}{\sum_{k=1}^{t+1} m_k}, \cdots, \frac{m_{t+1}}{\sum_{k=1}^{t+1} m_k}\right)$
  end for
  for $c \leftarrow 1$ to $|\mathcal{C}|$ **do**
    for $j \leftarrow 1$ to p **do**
      Sample $(\boldsymbol{\mu}_c)_j \sim N\left(\sum_{l\in c}(\boldsymbol{Y}_l)_j\left(n_c + \frac{\lambda_{\xi_j}^2}{(\boldsymbol{\phi}_c)_j}\right)^{-1}, \left(n_c + \frac{\lambda_{\xi_j}^2}{(\boldsymbol{\phi}_c)_j}\right)^{-1}\right)$
    end for
  end for
  for $c \leftarrow 1$ to $|\mathcal{C}|$ **do**
    for $j \leftarrow 1$ to p **do**
      Sample $(\boldsymbol{\phi}_{\boldsymbol{c}})_j \sim \text{GiG}(0.5, (\boldsymbol{\mu}_c)_j^2\lambda_{\xi_j}^2, 1)$
    end for
  end for
  for $j \leftarrow 1$ to p **do**
    $\theta' \leftarrow \frac{\prod_{c\in\mathcal{C}} \lambda_1 \exp\left(-\frac{1}{2}\lambda_1^2\frac{(\boldsymbol{\mu}_c)_j^2}{(\boldsymbol{\phi}_c)_j}\right)\theta}{\prod_{c\in\mathcal{C}} \lambda_1 \exp\left(-\frac{1}{2}\lambda_1^2\frac{(\boldsymbol{\mu}_c)_j^2}{(\boldsymbol{\phi}_c)_j}\right)\theta+\prod_{c\in\mathcal{C}} \lambda_0 \exp\left(-\frac{1}{2}\lambda_0^2\frac{(\boldsymbol{\mu}_c)_j^2}{(\boldsymbol{\phi}_c)_j}\right)(1-\theta)}$
    Sample $\xi_j \sim \text{Bernoulli}(\theta')$
  end for
  Sample $\theta \sim \text{Beta}\left(1 + \sum_{j=1}^p \xi_j, \beta_\theta + p - \sum_{j=1}^p \xi_j\right)$
end for

Appendix D. Additional Numerical Studies

We conduct three additional simulation studies to evaluate the proposed method under various setups: (a) when the covariance matrix of the true sampling distribution is diagonal, with different diagonal entries; (b) when the true sampling distribution exhibits skewness; and (c) when the true coordinates do not exhibit sparsity. We use the same hyperparameters as in the simulation studies, and run the proposed method with 1000 burn-in samples and 4000 post-burn-in samples. Each additional simulation is replicated 100 times.

In (a), we modify Scenario I in the simulation study with $s = 12$ and $K = 3$ by setting $\mathbf{\Sigma} = \mathrm{diag}(\sigma_1^2, \ldots, \sigma_p^2)$, where σ_j^2 are i.i.d. sampled from Gamma(100, 100) for $j \in [p]$. In all 100 replicated simulations, the proposed method successfully identifies the three clusters, yielding an average ARI of 0.97. In contrast, the four competitors tend to merge the two overlapping clusters, resulting in average ARIs of 0.54. These results highlight the robustness of the proposed Bayesian method, consistently outperforming alternative methods in terms of clustering accuracy.

In (b), we consider the sampling distribution to be a skewed t-distribution, with the same location and scale parameters as Scenario III in the manuscript. We set the skewness parameter to 10 and the degrees of freedom to 2. The average ARI across these 100 replicates is 0.84 for our proposed method, compared to 0.34 for PCA-KM. MClust and SKM fail to cluster in almost all simulation replicates, returning an average ARI of less than 0.01.

In (c), we set $n = 200$, $p = 400$, $K = 2$, $(\boldsymbol{\mu}_1^*)_S = r\mathbf{1}_s$, $(\boldsymbol{\mu}_2^*)_S = -r\mathbf{1}_s$, and assume $\mathbf{\Sigma}_1^* = \mathbf{\Sigma}_2^* = \boldsymbol{I}_p$. We fix the "signal-to-noise ratio" by setting $\|\boldsymbol{\mu}_1^* - \boldsymbol{\mu}_2^*\|_2^2 = 36$ and the sparsity support is defined as $S = [s]$, with s varying over $\{4, 10, 100, 400\}$. Our proposed Bayesian model successfully recovers the two clusters when $s = 4$ and 10. However, when $s = 100$ and 400, our model returns only one cluster, indicating poorer performance when the underlying truth is denser. Intuitively, this discrepancy arises because the spike-and-slab prior inherently introduces bias when the true model is not sparse. This bias affects the estimation of cluster centers, thereby influencing the update of cluster membership during subsequent MCMC iterations.

	Scenario I	
	$K = 3$, $s = 6$	$K = 3$, $s = 12$
Bayesian	369	368
PCA-KM	0.04	0.03
MClust	1.27	1.51
SKM	22.61	21.73
CHIME	11.28	11.37

Table A1: Empirical running times (seconds) for all methods in simulation studies.

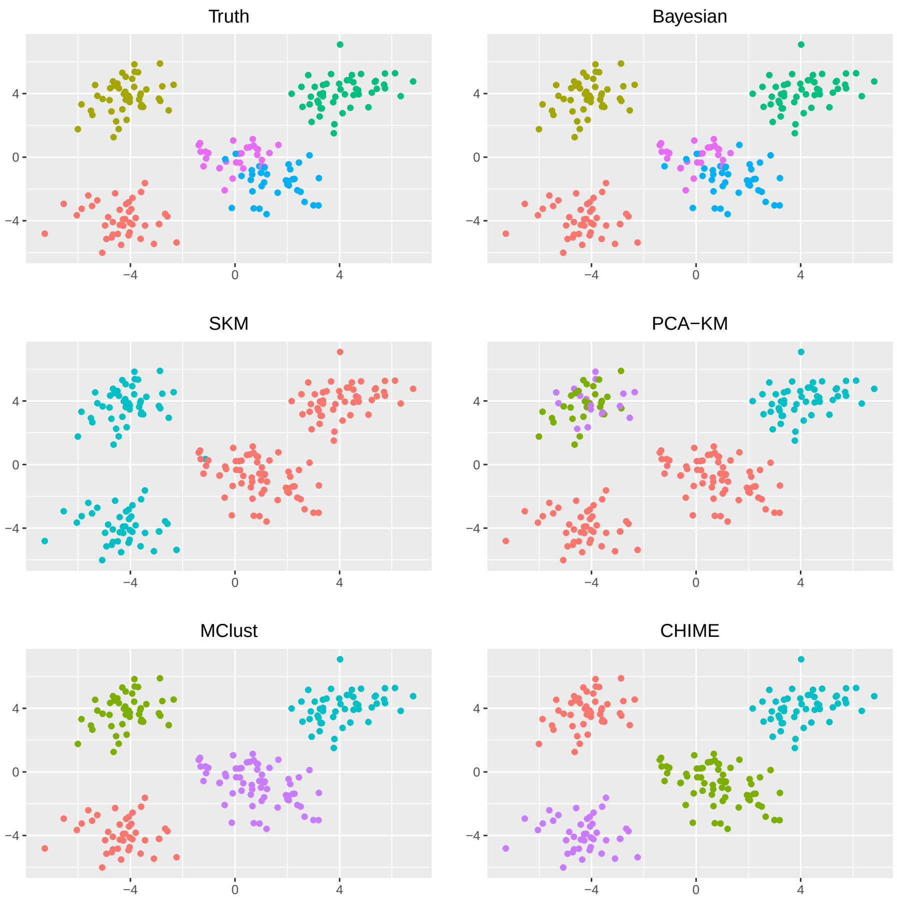

Figure A1: Clustering results of different methods compared with the true cluster memberships in the simulation Scenario I with $K^* = 5$ and $s = 6$ in a randomly selected simulation replicate. Data points are projected onto the subspace of the first two coordinates and different colors correspond to different estimated cluster memberships of the data points.

Methods	ARI	NMI
KM	0.79	0.77
tSNE-KM	0.63	0.73
PCA-KM	0.81	0.79
NMF-KM	0.77	0.78
SKM	0.15	0.23
MClust	0.83	0.79

Table A2: ARIs and NMIs of different methods on scRNA-Seq data. The number of clusters is set to be truth ($K = 8$) for all methods.

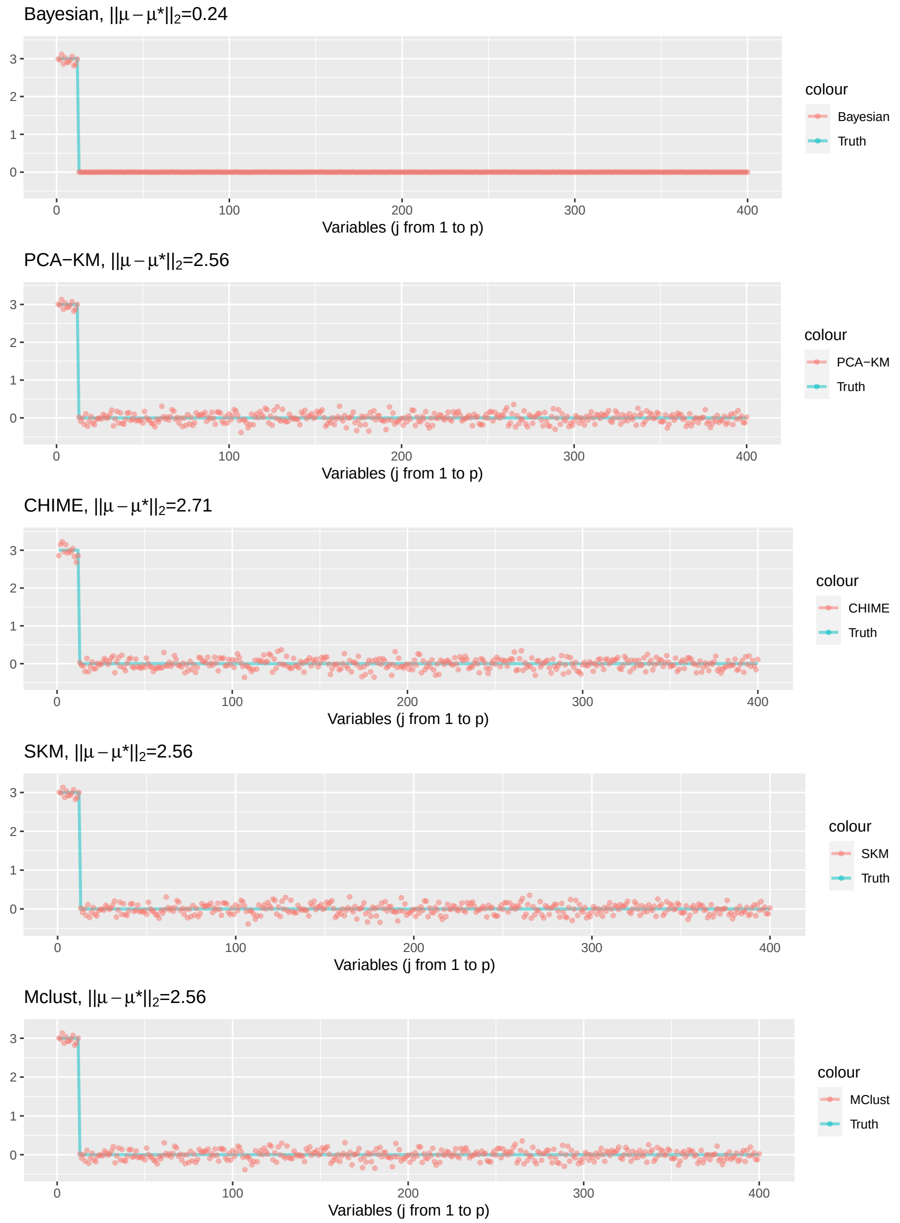

Figure A2: Estimation of mean vector $\boldsymbol{\mu}_1^*$ of different methods in Scenario I with $K^* = 3$ and $s = 12$ in a randomly selected simulation replicate.

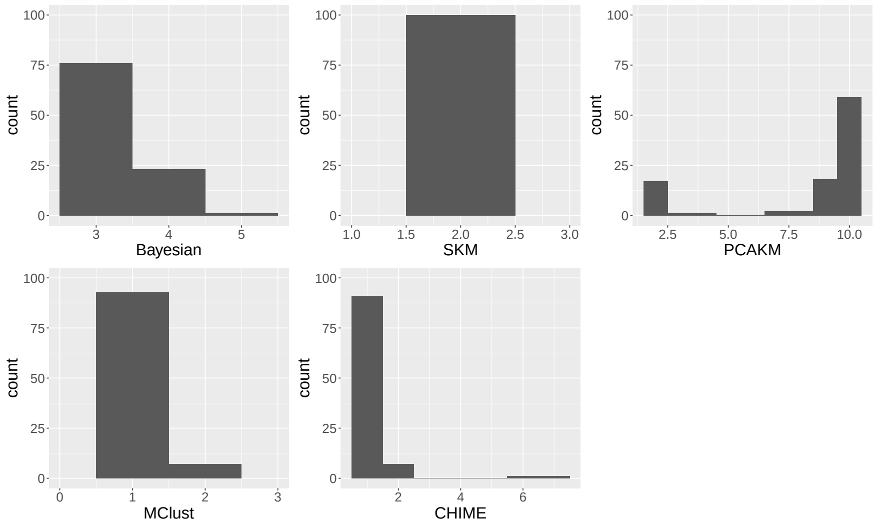

Figure A3: Histograms of estimated number of clusters under different methods in Scenario III of simulation studies.

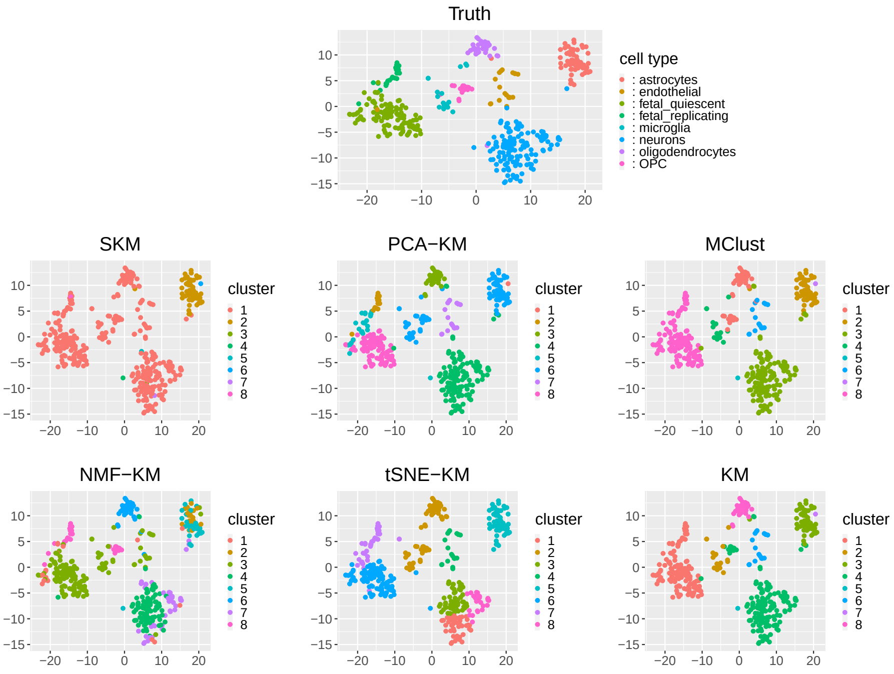

Figure A4: Clustering results of scRNA-Seq data corresponding to alternative methods. The number of clusters is set to be truth ($K = 8$) for all methods.
